# Crucial Role of the Accessory Genome in the Evolutionary Trajectory of *Acinetobacter baumannii* Global Clone 1

**DOI:** 10.3389/fmicb.2020.00342

**Published:** 2020-03-18

**Authors:** Verónica Elizabeth Álvarez, María Paula Quiroga, Angélica Viviana Galán, Elisabet Vilacoba, Cecilia Quiroga, María Soledad Ramírez, Daniela Centrón

**Affiliations:** ^1^Laboratorio de Investigaciones en Mecanismos de Resistencia a Antibióticos, Instituto de Investigaciones en Microbiología y Parasitología Médica, Facultad de Medicina, Universidad de Buenos Aires - Consejo Nacional de Investigaciones Científicas y Tecnológicas (IMPaM, UBA-CONICET), Buenos Aires, Argentina; ^2^Instituto de Investigaciones en Microbiología y Parasitología Médica, Facultad de Medicina, Universidad de Buenos Aires - Consejo Nacional de Investigaciones Científicas y Tecnológicas (IMPaM, UBA-CONICET), Buenos Aires, Argentina; ^3^Center for Applied Biotechnology Studies, Department of Biological Science, California State University Fullerton, Fullerton, CA, United States

**Keywords:** *A. baumannii*, international clone 1 (GC1), accessory genome, genomic plasticity, high-risk clone

## Abstract

*Acinetobacter baumannii* is one of the most important nosocomial pathogens able to rapidly develop extensive drug resistance. Here, we study the role of accessory genome in the success of the globally disseminated clone 1 (GC1) with functional and genomic approaches. Comparative genomics was performed with available GC1 genomes (*n* = 106) against other *A. baumannii* high-risk and sporadic clones. Genetic traits related to accessory genome were found common and conserved along time as two novel regions of genome plasticity, and a CRISPR-Cas system acquired before clonal diversification located at the same loci as “sedentary” modules. Although identified within hotspot for recombination, other block of accessory genome was also “sedentary” in lineage 1 of GC1 with signs of microevolution as the AbaR0-type genomic island (GI) identified in A144 and in A155 strains which were maintained one month in independent experiments without antimicrobial pressure. The prophage YMC/09/02/B1251_ABA_BP was found to be “mobile” since, although it was shared by all GC1 genomes, it showed high intrinsic microevolution as well as mobility to different insertion sites. Interestingly, a wide variety of Insertion Sequences (IS), probably acquired by the flow of plasmids related to Rep_3 superfamily was found. These IS showed dissimilar genomic location amongst GC1 genomes presumably associated with promptly niche adaptation. On the other hand, a type VI secretion system and three efflux pumps were subjected to deep processes of genomic loss in *A. baumannii* but not in GC1. As a whole, these findings suggest that preservation of some genetic modules of accessory genome harbored by strains from different continents in combination with great plasticity of IS and varied flow of plasmids, may be central features of the genomic structure of GC1. Competition of A144 and A155 versus A118 (ST 404/ND) without antimicrobial pressure suggested a higher ability of GC1 to grow over a clone with sporadic behavior which explains, from an ecological perspective, the global achievement of this successful pandemic clone in the hospital habitat. Together, these data suggest an essential role of still unknown properties of “mobile” and “sedentary” accessory genome that is preserved over time under different antibiotic or stress conditions.

## Introduction

The broad diversification of species among the genus *Acinetobacter* occurred mostly due to Lateral Genetic Transfer (LGT) events, and to some allelic recombination at specific hotspots (Touchon et al., 2014; Holt et al., 2016). The size of the pangenome of *A. baumannii* has larger values than the rest of species within the genus, evidencing also a high biochemical diversity (Touchon et al., 2014; Chan et al., 2015). *A. baumannii* is well known as an opportunistic pathogen mainly implicated in ventilator-associated pneumonia, catheter-related bloodstream, urinary tract, and wound infections (Falagas et al., 2006; Peleg et al., 2008; Roca et al., 2012; Park et al., 2013; Inchai et al., 2015). This species has a highly plastic genome evidenced by the amount of insertions, deletions, inversions and SNPs reported (Peleg et al., 2008; Imperi et al., 2011; Antunes et al., 2014), which may contribute to its adaptation to several niches and the evolution to extensive (XDR) and pandrug resistance (PDR) phenotypes (Adams et al., 2008; Vallenet et al., 2008; Arduino et al., 2012; Ou et al., 2015; Holt et al., 2019), harboring a pangenome of over 9000 gene families (Antunes et al., 2011; Touchon et al., 2014).

Comparative typing of European outbreak strains of *A. baumannii* demonstrated the occurrence of three successful clones identified as “International Clones I–III” (IC1-3), or also known as Global Clones (GC) (Diancourt et al., 2010). Homologous recombination near the origin of replication was the mechanism associated with the diversification of these GC (Snitkin et al., 2013).

Global Clone 1 has been showed to have a broad international distribution in more than 30 countries from all continents (Karah et al., 2012). Regarding to the evolutionary trajectory of GC1, genomic studies showed that the most recent common ancestor emerged in the 1960s and then diverged into two phylogenetically distinct lineages (Holt et al., 2016; Hamidian et al., 2019). In the 1970s, the main lineage acquired an AbaR0-type GI where the resistance mechanisms to the older antibiotics usually are found (Holt et al., 2016). This epidemic clone has diversified into multiple successful extensively antibiotic-resistant subclones that differ in their surface structures (Holt et al., 2016). Concerning the genomic topology of GC1, four hotspots of recombination non-related to the accessory genome were identified within GC1 (Holt et al., 2016). From those, two hotspots of recombination are associated with biosynthesis of exopolysaccharides via the K locus and OC locus, the third is the gene encoding the outer membrane protein CarO, and the fourth introduces resistance to third-generation cephalosporins via the insertion sequence-enhanced expression of the intrinsic AmpC β-lactamase (Holt et al., 2016). Also, two CRISPR-Cas systems have been identified in some strains of *A. baumannii* (Di Nocera et al., 2011; Hauck et al., 2012). The AYE strain, whose genome is taken in many studies as the basis for GC1 studies (Touchon et al., 2014; Karah et al., 2015), and other *A. baumannii* clones were identified to harbor the subtype I-Fb, indicating potential inter-strain horizontal transfer of this CRISPR-Cas system (Di Nocera et al., 2011; Karah et al., 2015). Based on distinct assortment of spacers harbored by each strain, a CRISPR-based sequence type (Schouls et al., 2003) identified a subclone of *A*. *baumannii* GC1 that it is likely it has been originated in Iraq and spread later to the United States and Europe (Karah et al., 2015). Holt et al. (2016) reported the existence of two lineages within GC1. Lineage 1 genomes carried an AbaR0-type island with a Tn*6019*–like element inserted within the *comM* gene (Hamidian et al., 2014; Holt et al., 2016). In turn, a deletion in *intI1* defines AbaR3-type GI (Holt et al., 2016). Successive microevolution of AbaR0 and AbaR3-types includes acquisition and loss of Antimicrobial Resistance Genes (ARG), which gave rise to the different island scaffolds (Post and Hall, 2009; Post et al., 2010, 2012; Krizova et al., 2011; Nigro et al., 2011; Holt et al., 2016). Lineage 2 genomes either lack a transposon in *comM* or had acquired the transposon Tn*6022* or its variants which may lead to the formation of AbaR4 GI (Hamidian and Hall, 2011; Holt et al., 2016).

Concerning our local epidemiology, recent studies of Carbapenem Resistant *A. baumannii* (CRAB) isolates, revealed that GC1 is the most widespread and common clone of CRAB in Argentina (Rodríguez et al., 2018).

Previously, we reported the genome of A144 (CC1/CC231) and A155 (CC1/CC231) *A. baumannii* strains from Argentina which belonged to GC1 (Vilacoba et al., 2014; Arivett et al., 2015). These strains were isolated in the 1990s when carbapenems were recently introduced in Argentina (Vilacoba et al., 2014). Since at that time clonal complex CC113 was predominant (Stietz et al., 2013), A155 (CC1/CC231) was considered among the first GC1 isolates in Argentina (Ramírez et al., 2013).

The aim of this work was to examine the role of the accessory genome of the high-risk clone GC1 across time and continents from genomic and functional approaches. Although previous comparative genomic studies evidenced genetic variability across all *A. baumannii* strains (Adams et al., 2008; Vallenet et al., 2008; Di Nocera et al., 2011; Sahl et al., 2013; Touchon et al., 2014; Holt et al., 2019; Meumann et al., 2019), no data, excluding evolution to antimicrobial resistance (Karah et al., 2017; Hamidian and Hall, 2018b; Holt et al., 2019), is focused on the features of the accessory genome of prevalent clones. Interestingly, two patterns of preservation of the accessory genome within GC1 strains regardless their site or time of isolation were found: (i) “sedentary” modules such as two novel regions of genome plasticity, the AbaR GI in lineage 1, and even a CRISPR-Cas type-If system located in the same loci; and (ii) a “mobile” module as the case of the putative prophage YMC/09/02/B1251_ABA_BP shared by all 106 GC1 genomes which showed high genomic plasticity evidenced by intrinsic microevolution as well as mobility to different insertion sites amongst GC1’s chromosomes. Because AbaR GI is widespread among *A. baumannii* clinical samples, particularly in GC1, this GI was used as a biological model for comparative genomics and experimental studies of maintenance. We found that AbaR0-type GI from A144 (CC1/CC231) as well as in A155 (CC1/CC231) was maintained at least over one month in three independent experiments without antimicrobial pressure, while *in silico* analysis revealed AbaR0-type GI were all different including AbaR0-type GI from A144 (CC1/CC231) which showed signs of microevolution events compared to A155 (CC1/CC231). On the other hand, a Type VI Secretion System (T6SS) and three efflux pumps showed to be subjected to deep processes of genomic loss in *A. baumannii* but not in GC1. As a whole, these studies highlighted that the conservation of genetic elements of the accessory genome may play a still unknown role in the success of this high-risk clone.

## Materials and Methods

### Bacterial Strains Used for Experimental Assays

The multidrug resistant GC1 *A. baumannii* A144 and A155 strains (CC1/CC231), and A118 (ST 404/ND) which belongs to an sporadic clone were isolated from the same hospital H1 from Argentina in 1997 (Vilacoba et al., 2014), 1994 (Arivett et al., 2015), and 1995 (Traglia et al., 2014), respectively. These strains that were available in our laboratory were used to perform experimental investigation concerning maintenance of accessory genome along time as well as clone competition assays (see below).

### Previous DNA Sequencing of H1 Strains

Strains from H1 Hospital from Argentina, *A. baumannii* A144 and A155 (CC1/CC231), and the sporadic clone A118 (ST 404/ND) that were the basis of this study, were previously sequenced (Traglia et al., 2014; Vilacoba et al., 2014; Arivett et al., 2015). Briefly, Whole-Genome Shotgun (WGS) sequencing was performed using Illumina MiSeq-I, using Nextera XT libraries for sample preparation. Reads were assembled with Ray assembler^1^. The draft genome sequence of A144 (CC1/CC231) consist of 92 contigs (length > 500 bp), a total sequence of 4,312,914 bp with an *N*_50_ contig size of 89,819. The GC% average was 39.2. Using RAST (Aziz et al., 2008) we identified 4,151 possible ORFs, 74 copies of 16S-23S-5S rRNA operons and 69 tRNA genes (Vilacoba et al., 2014). The WGS project has been deposited at DDBJ/EMBL/GenBank under the Accession Number (AN) JQSF00000000. The *de novo* assembly of A155 (CC1/CC231) resulted in a 3,933,455 bp genome encoding 55 tRNAs and 3,760 genes with 3,704 proposed CDSs (Arivett et al., 2015). The first version of the *de novo* whole-genome assembly of A155 (CC1/CC231) was deposited into GenBank under Bioproject ID PRJNA261239 with the accession number JXSV00000000, version JXSV01000000 with 53 contigs (Arivett et al., 2015). The draft genome of A118 (ST 404/ND) (AN: AEOW00000000) consist of 156 scaffolds with a total length of 3,730,023 bp (Ramirez et al., 2011). The genome has an average GC content of 38.4%, 88 tRNA genes and 3,520 coding sequences were identified, of which 93.64% was annotated and manually curated using blastn results (Traglia et al., 2014).

### Data Collections for Comparative Genomics

In order to perform accurate comparative genomics in combination with experimental assays, we identified four groups of genomes that were clustered to do our analysis (see below).

Global Clone 1 Group 1 (CC1/CC231) was composed by 18 genomes as follows: (i) A144 and A155 from Argentina (Vilacoba et al., 2014; Arivett et al., 2015); (ii) 14 GC1 complete genomes retrieved from GenBank which correspond to strains AYE (AN: NC_010410.1), D36 (AN: CP012952.1), A1 (AN: CP010781.1), AB307-0294 (AN: CP001172.2), AB5075-UW (AN: CP008706.1), AB0057 (AN: CP001182.1), USA15 (AN: NZ_CP020595.1), A85 (AN: NZ_CP021782.1), A388 (AN: NZ_CP024418.1), AR_0083 (AN: NZ_CP027528.1), DA33382 (AN: NZ_CP030106.1), 9102 (AN: NZ_CP023029.1), 11W359501 (AN: CP041035.1), NCTC13421 (AN: NZ_LS483472.1); and (iii) two highly quality genomes available for GC1, strains NIPH 527 and NIPH 290, isolated in 1984 and 1994, respectively (APQW00000000.1 and APRD00000000.1) (Table 1). Both GC1 strains, A144 and A155, were relevant for our country since they were isolated in the 1990s when at that time clonal complex CC113 was predominant (Stietz et al., 2013). Therefore, A155 was considered among the first GC1 isolates emerging in Argentina (Ramírez et al., 2013) which is in agreement with the introduction of carbapenems in our country (Vilacoba et al., 2014). This GC1 Group 1 of genomes include all GC1 complete genomes till July, 2019 and they were used to perform the comparative genomics for RGP, prophages, plasmids, CRISPR-Cas system, Insertion Sequences (IS), Transposons, AbaR-types GI, and genes encoding AdeABC, AdeIJK, and AdeFGH efflux pumps, among others. The site of insertion of RGP, prophages, CRISPR-Cas system, IS and AbaR-types GI, were identified for this group, since most of these are complete genomes and allow accurate identification.

**TABLE 1:** General features of GC1 Group 1 and Outgroup Group 3 genomes.

**Group**	**Strain**	**Country**	**Year**	**MLST^a^**	**GC**	**GC1 lineage**	**AbaR type**	***intI1* in AbaR**	**CRISPR**
GC1 Group 1	A144	Argentina	1997	CC1/CC231	1	1	AbaR0-type	Complete	Yes
	A155	Argentina	1994	CC1/CC231	1	1	AbaR0-type	Complete	Yes
	A1	United Kingdom	1982	CC1/CC231	1	1	AbaR24	Complete	Yes
	AB307-0294	United States	1997	CC1/CC231	1	1	−	−	Yes
	AYE	France	2001	CC1/CC231	1	1	AbaR1	Complete	Yes
	AB0057	United States	2004	CC1/CC231	1	1	AbaR3	Deleted	Yes
	AB5075-UW	United States	2008	CC1/CC231	1	1	AbaR11	−	Yes
	D36	Australia	2008	CC1/CC231	1	2	AbaR4	−	Yes
	USA15	South Korea	2013	CC1/CC231	1	1	AbaR10	−	Yes
	A85	Australia	2003	CC1/CC231	1	1	AbaR3	Complete	Yes
	A388	Greece	2002	CC1/CC231	1	1	AbaR28	Complete	Yes
	AR_0083	United States	Unknown	CC1/CC231	1	1	AbaR0-type	Complete	Yes
	DA33382	Germany	Unknown	CC1/CC231	1	1	AbaR0-type	Complete	Yes
	9102	Mexico	2010	CC1/CC231	1	1	−	−	Yes
	11W359501	United Kingdom	2015	CC1/CC231	1	1	AbaR0-type	Complete	Yes
	NCTC13421	United Kingdom	2004	CC1/CC231	1	1	AbaR0-type	Complete	Yes
	NIPH 527	Netherlands	1984	CC1/CC231	1	1	AbaR21	Complete	Yes
	NIPH 290	Czechia	1994	CC1/CC231	1	1	AbaR0-type	Complete	Yes
Outgroup Group 3	ACICU	Italy	2005	CC2/CC208	2	−	AbaR2	Complete	No
	Naval-13	United States	2006	ST3/CC928	3	−	AbaR4	−	No
	A118	Argentina	1995	ST404/*	−	−	−	−	No
	AB33405	Argentina	2013	CC79/CC113	−	−	AbaR4	−	No
	ATCC 17978	United States	1951	ST437/ST112	−	−	AbaR4	−	No

^*a*^Pasteur’s/Oxford’s MLST schemes; Clonal complexes (CCs) are numbered according to the most prevalent clone. STs are indicated when singletons. *The sequence type (ST) was not found in Oxford’s MLST database.

Global Clone 1 Group 2 (CC1/CC231) was composed by 27 genomes as scaffolds and 61 genomes as contigs from GenBank (until July, 2019) that were identified as GC1 using the program mlst^2^ which scans genome files against traditional PubMLST typing schemes (Supplementary Table 1). The 88 genomes of this GC1 Group 2 were used for searching RGP, genes encoding AdeABC, AdeIJK, and AdeFGH efflux pumps, CRISPR-Cas system as well as identification of K and OC locus in combination with GC1 Group 1.

In summary, all GC1 genomes deposited in GenBank till July, 2019 were included in our analysis.

In order to identify exclusive accessory genome of GC1, we clustered in the Outgroup Group 3 five genomes belonging to other high-risk epidemic clones such as ACICU (AN: CP000863.1) as representative of GC2, Naval-13 (AN: AMDR01000001.1) as representative of GC3, AB33405 (NZ_JPXZ00000000.1;^3^) as representative of local epidemic clone CC113 and both ATCC 17978 (AN: CP018664.1), and A118 (AN: AEOW01000000) as sporadic clones (Table 1).

The Outgroup Group 4 was composed by 2407 genomes as contigs and 549 genomes as scaffolds of *A. baumannii* that were identified as non-GC1 using the program mlst^2^ (Supplementary Table 1). The 2956 genomes of this group were downloaded from GenBank (until August, 2019). The Outgroup Group 4 was used to analyze if the CRISPR-Cas system identified in GC1 Group 1 and 2 was also present in this group.

### Antibiotic Susceptibility Assays

Disk diffusion antibiotic susceptibility and/or minimal inhibitory concentration tests were performed following the procedures recommended by the CLSI (CLSI, 2018) with antimicrobial commercial disks of ampicillin-sulbactam, sulbactam, ceftazidime, cefepime, cefotaxime, imipenem, meropenem, colistin, gentamicin, amikacin, minocycline, tetracycline, ciprofloxacin, levofloxacin, trimethoprim-sulfamethoxazole, rifampin, and chloramphenicol from Britania (Table 2). When clinical breakpoints were not available from CLSI, only the values obtained were shown. For sulbactam we used a provisional susceptibility breakpoint of ≤4 μg/ml derived from the CLSI breakpoint for ampicillin/sulbactam (≤8/4 μg/ml; Krizova et al., 2013).

**TABLE 2:** Antimicrobial susceptibility profiles of strains under study.

**Strain**	**Susceptibility profiles Disk diffusion (mm)/MIC (μg/ml)**																
	**AMS**	**SUL**	**CAZ**	**FEP**	**CTX**	**IPM**	**MEM**	**CL**	**GEN**	**AMK**	**MIN**	**TET**	**CIP**	**LEV**	**SXT**	**RIF**	**CHL**
A144	R	−/16	R/32	I	R/ ≥ 64	S	S/2	−/S < 2	R/ ≥ 16	S/16	S	R	R/32	R	R	(12)/4	−/8
A155	R	−/16	R/32	I	R/ ≥ 64	S	S/2	−/S < 2	R/ ≥ 16	S/16	S	R	R/32	R	R	(16)/4	−/8
A118	−	−/4	−	−	−	S	S/1	−	S/ ≤ 2	S	−	−	S	S	−	4	−
ATCC 17978	−	−/2	−	−	−	−	−	−	−	−	−	−	−	−	−	8	−

*AMS, ampicillin-sulbactam; SUL, sulbactam; CAZ, ceftazidime; FEP, cefepime; CTX, cefotaxime; IPM, imipenem; MEM, meropenem; CL, colistin; GEN, gentamicin; AMK, amikacin; MIN, minocycline; TET, tetracycline; CIP, ciprofloxacin; LEV, levofloxacin; SXT, trimethoprim-sulfamethoxazole; RIF, rifampicin; CHL, chloramphenicol; R, resistant; I, intermediate; S, susceptible. −, not determined. In the absence of CLSI 2018 interpretive breakpoint, the zone diameters are shown.*

### Comparison of AbaR Genomic Islands

Software ACT was used to compare AbaR islands (Carver et al., 2005).

### Phylogenetic Analysis

The core genome of the 18 genomes from GC1 Group 1 was calculated with GET_HOMOLOGUES software (Contreras-Moreira and Vinuesa, 2013). Core genome SNPs were detected using SNP-sites (Keane et al., 2016). SNP likely to have been introduced together via a homologous recombination event were detected and analyzed using Gubbins with default parameters (Croucher et al., 2015). Phylogenetic tree was built using Gubbins based on the alignment of the non-recombinant SNP obtained and using a maximum-likelihood (ML) phylogeny inferred from the alignment of these SNP. The five genomes from Outgroup Group 3 were used as outgroup. The figure of the phylogenetic tree was obtained using Evolview v3 (Subramanian et al., 2019).

### Maintenance Studies of the AbaR Genomic Islands

Strains A144 and A155 were grown each at 37°C overnight in 2 ml LB broth. Subcultures were carried out for 30 days (Moffatt et al., 2010). At 1st, 7th, and 30th day, 30 colonies were tested for presence of AbaR GI by PCR using two pairs of specific primers that detect the disruption of the gene *comM*, which target the junction with the 3′ATPase (4R: 5′-AATCGATGCGGTCGAGTAAC-3′ and 4F: 5′-TATCAGCAGCAAAACGATGG-3′) and the junction with the 5′ATPase (2R: 5′-TTGGGGATTCTGTCCGTAAG-3′ and 2F: 5′-TCCATTTTACCGCCACTTTC-3′) (Shaikh et al., 2009; Ramírez et al., 2013). The experiments were performed in triplicates.

### *In vitro* Competition and Fitness Measurements

A144 or A155 and A118 isolates were diluted to 1.6 × 10^8^ (OD_600_ 0.2) colony-forming units (CFU)/ml, equal volumes were combined, thus the initial ratio of the isolate pairs was close to 1:1, then 10 μl of the mixture was added to 20 ml LB broth and grown at 37°C with agitation at 200 rpm. At 24-h intervals, 10 μl of bacterial subcultures were transferred to fresh LB broth; meanwhile, 10 μl was inoculated on MH agar plates, and 10 μl on MH agar plates containing 16 μg/ml gentamicin. CFU of A144 (resistant to gentamicin, Table 2) and A118 (susceptible to gentamicin, Table 2) were counted, and after 96 h, adaptive difference of each pair (A144/A118) was calculated as $\text{S}=\text{ln}\,\left[{\left(\frac{\frac{{\text{r}}_{\text{t}}}{{\text{S}}_{\text{t}}}}{\frac{{\text{r}}_{\text{t}-1}}{{\text{S}}_{\text{t}-1}}}\right)}^{\left(\frac{1}{\text{y}}\right)}\right]$ relative adaptive fitness as *F* = 1 + *S*, and the fitness cost as *C* = (1−*F*)*x*100%, where *S* is the selection coefficient and show the difference in fitness between two competing strains at time *t*, *r*_*t*_ = number of drug-resistant colonies and *s_*t*_* = number of drug-susceptible colonies, *r*_*t–1*_ and *s*_*t–1*_ are the number of drug-resistant and drug-susceptible colonies at the preceding time point, respectively, and the quotient of the ratios of the cell numbers was standardized with 1/y, where “y” is the number of bacterial generations during the assay (Sander et al., 2002; Guo et al., 2012; Li et al., 2018). Here the exponent was 1/8 because cell numbers were determined every eight generations. The terms *rt*/*rt*_1 and *st*/*st*_1 give the growth rates for drug-resistant and drug-susceptible strains, respectively. Hence, *S* is the natural logarithm of the quotient of the growth rates of the competing strains. *S* is positive if resistance increases bacterial fitness compared to that of the drug-susceptible competitor strain (Sander et al., 2002).

### Statistical Analysis

Statistical analysis was performed with the software GraphPad Prism version 8 using one-way analysis of variance (ANOVA). *P* < 0.05 was considered to be statistically significant (Li et al., 2018).

### Detection of Regions of Genomic Plasticity

As defined by Mathee et al. (2008) the minimum size of a region of genomic plasticity (RGP) is defined as a block of at least four contiguous ORFs that are not conserved in all strains from a species. RGP were identified in GC1 Group 1 using RAST (Aziz et al., 2008), in combination with Prokka (Seemann, 2014), ISFinder (Siguier, 2006), PHASTER (Arndt et al., 2016), and IslandViewer4 (Bertelli et al., 2017).

### Mobilome and Resistome Analysis

Search of IS and transposons was done using ISFinder (Siguier, 2006) and blastn (Altschul et al., 1990) with a cut-off *E*-value of E^–10^; genomic islands were predicted with IslandViewer4 (Bertelli et al., 2017) and phages with PHASTER (Arndt et al., 2016). ARG were identified using RESfinder (Zankari et al., 2012) and blastn (Altschul et al., 1990) with a cut-off *E*-value of E^–10^. The ARG content previously reported was also included, when required (Holt et al., 2016). BM4587 (AN: KR297239.1) was taken as reference to compare the 7,591 bp comprising the *adeL*, *adeF*, *adeG*, and *adeH* genes of the *adeFGH* efflux pump and its regulator with a wild type expression level (Coyne et al., 2010). The *bla*OXA-51-like genes were identified by Single-Locus-Sequence-Based Typing (SBT) analyzing 825 bp (forward primer, 5′-ATGAACATTAAAGCACTCTTAC-3′; reverse primer, 5′-CTATAAAATACCTAATTGTTCT-3′) by blastn (Pournaras et al., 2014). The *ampC* alleles were assigned using the database hosted at the pubmlst platform for *A*. *baumannii*^4^ (Karah et al., 2017). The *bla*_TEM_ promoters were identified by blastn (Lartigue et al., 2002). Mutations in *rpoB* were analyzed by the comparison of the nucleotide sequences with the deduced amino acid sequence of *rpoB* from *Escherichia coli* strain ATCC 8739 (ACA79637.1) and ACICU (YP_001844962.1) using blastn (Giannouli et al., 2012). To determine the Quinolone Resistance-Determining Regions (QRDR), the wild-type *A. baumannii* GyrA (X82165) and ParC (X95819), QRDR GyrA81, ParC84, and ParC88 were compared with those of *E. coli* at positions Ser-83, Ser-84, and Glu-88, respectively (Ostrer et al., 2019).

### Pangenome Calculation

The pangenome, the soft-core genome, and the core genome were identified using the GET_HOMOLOGUES software (Contreras-Moreira and Vinuesa, 2013) based on the GC1, GC2, GC3, CC113, and sporadic clones genomes analyzed using a minimal identity value of 70% and a minimal query coverage of 80% sequence identity in blastn query/subject pairs.

### Plasmid Recognition

Genes related to plasmids were identified mapping the A144 and A155 contigs using the AYE strain genome as reference (AN: CU459141.1) with MAUVE version 2.4.0 (Darling et al., 2004). Contigs not mapping with AYE genome where blasted against the non-redundant (nr) GenBank database with a cut-off *E*-value of E^–10^ and analyzed for plasmid replicons.

### Detection of Recombination Hotspots

Recombination hotspots (HS) are regions in a genome that exhibit elevated rates of recombination relative to a neutral expectation. Hotspots in the present study are associated with both an increase in mutations in a region of the genome and incorporation of accessory DNA. HS were identified using the same methodology as in Touchon et al., 2014. The GC1 Group 1 core genome was used to identify and locate large integration/deletion (indel) regions. All regions including more than ten genes between two consecutive core genes of the genomes were considered as large indel regions. The relative positions of these regions were defined by the order of the core genes in *A. baumannii* AYE. This strain was used as a reference to assemble the GC1 Group 1 genes.

### Identification of K and OC Loci

The exopolysaccharide loci were identified by blastn search for the flanking genes (K: *fkpA*, *lldP*; OC: *ilvE*, *aspS*) as described previously (Holt et al., 2016). Each locus was matched against a set of known K loci or OC loci (Kenyon and Hall, 2013; Holt et al., 2016).

### CRISPR-Cas System Predictions

CRISPR were predicted using CRISPRfinder^5^ and CRISPRone^6^ using the default parameters.

## Results

### Genomic Analysis of GC1 Strains From H1 Hospital

Comparative genomics was carried out for A144 and A155 strains from H1 Hospital with all GC1 Group 1 which were isolated from four different continents (America, Oceania, Asia and Europe) and with other epidemic and sporadic clones of the Outgroup Group 3 genomes (Table 1). We found that A144 genome has 99% Average Nucleotide Identity (ANI) with A155 isolated from the same hospital. Both A144 and A155 have 97% ANI with GC1 Group 1 strains (Table 1). A144 and A155 exhibited 81% ANI with ACICU (IC2), 83% with Naval-13 (IC3), and 79% with AB33405 (GC113), A118 (sporadic clone), and also with ATCC 17978 (sporadic clone) of Outgroup Group 3 (Table 1).

**FIGURE 1:**
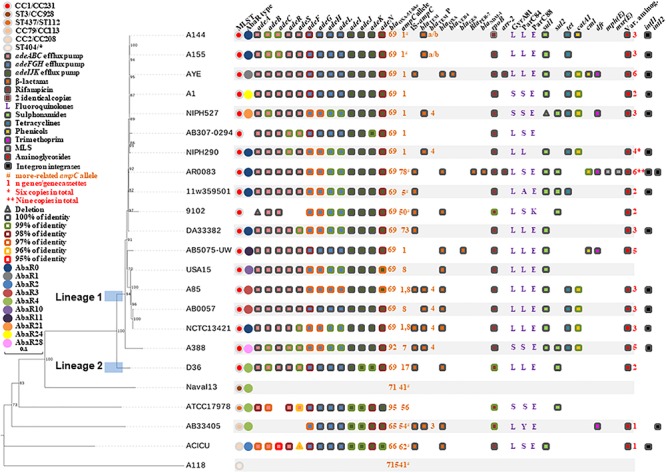
Molecular phylogenetic analysis and antimicrobial resistance determinants of GC1 Group 1 and Outgroup Group 3. The evolutionary history was inferred by using the Maximum Likelihood method based on the General Time Reversible model. The inset legend indicates the genetic determinants highlighted. When required, blastn was used with a cut-off *E*-value of E^–10^.

We found that 2,840 genes were part of the core genome comprising the 18 GC1 complete genomes under scrutiny. The phylogenetic tree obtained with the non-recombinant SNPs found in the core genes of the GC1 Group 1 genomes reflected the two lineages previously reported (Holt et al., 2016), evidencing that A144 and A155 genomes belonged to lineage 1 and they were closely related to the strain AYE (Figure 1).

A difference of one order of magnitude was found when analyzing the number of unique genes within the chromosomes of GC1 (from 15 to 74 unique genes) when compared to GC2, GC3, CC113, and sporadic clones (from 362 to 626 unique genes). A144 contained 37 unique genes when we compared its chromosome with GC1 Group 1 (Supplementary Table 2); most of them were coding sequences of unknown function. Only ten unique genes out of 37 had an assigned function, such as two phage-related proteins, a copper resistance system oxidase (*copA*), a zinc transporter (*zitB*), a cobalt transporter (*czcD*), and two transcriptional regulatory proteins (*qseB_2* and *qseB_3*) that probably belong to a novel heavy metal resistance GI (Data not shown). A155 contained sixteen unique genes not related to pathogenicity or antimicrobial resistance (Supplementary Table 2). The emergence of *aac(6*′*)-Ian* gene cassette was found unique in AYE strain [94.13% *aac(6*′*)-Ian* allele (CP023420.1)].

The GC1 Group 1 possessed 39 unique genes compared to Outgroup Group 3 that most of them were scattered in the topology of the chromosome (Supplementary Table 2). For example the array of the 6 *cas* genes (*cas1-cas3-cas8f-cas5f-cas7f-cas6f*) from the type IF-b CRISPR-Cas system that we found in all GC1 genomes (GC1 Group 1 and 2) located in the same loci in GC1 Group 1, a LysR_substrate_binding domain related to LysR family of transcriptional regulators (ABAYE2346) that have been identified that regulate a diverse set of genes, including those involved in virulence, metabolism, quorum sensing and motility (Maddocks and Oyston, 2008), three genes related to the lipid metabolic process [coenzyme A (CoA) transferase (ABAYE2345), acyl-CoA desaturase (ABAYE1343), and hydroxymethylglutaryl CoA reductase (ABAYE2344)] among other genes that coded for transferases, reductases, hydrolases and hypothetical proteins (Supplementary Table 2).

Otherwise, we found that the Outgroup Group 3 ACICU, A118, Naval-13, AB33405, and ATCC 17978 chromosomes had 358, 595, 412, 311, and 395 unique genes, respectively, when we compared them with the pangenome of the GC1 Group 1, most of them coding sequences of unknown function. The smaller number of unique genes in GC1 could be related to the need of balance among new genes putatively acquired by events of the LGT and the genes of the core genome that may be necessary to preserve synteny and/or functionality. Interestingly, in the case of ATCC 17978, the previously described Tn*6171* transposon of 49.9 kb length carrying a potential siderophore synthesis gene cluster and transposition genes related to Tn*7* (Hamidian et al., 2015) without the typical invasion of a class 2 integron (Ramírez et al., 2010) was found amongst the unique genes, evidencing its independent acquisition by LGT events.

**FIGURE 2:**
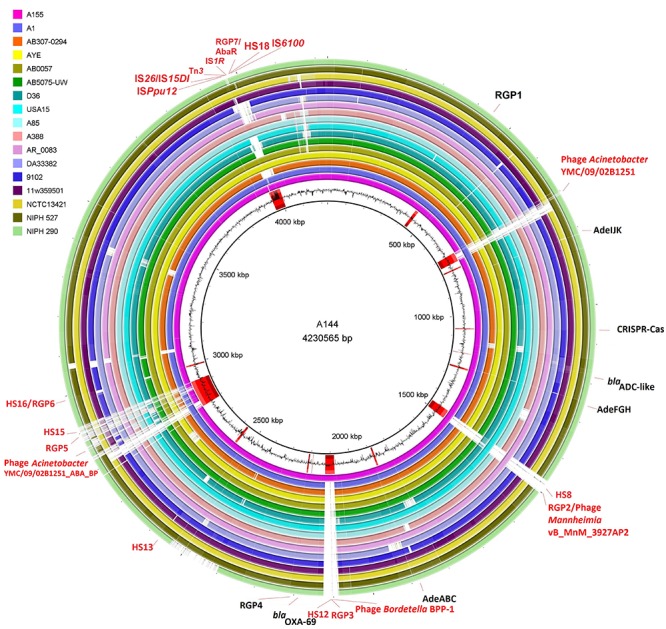
Prediction of elements of the accessory genomes identified in GC1 Group 1 genomes from this study. Genetic and physical map of GC1 chromosomes. The inner black circle belongs to A144 chromosome and the inset legend indicates the remaining GC1 Group1 chromosomes. The outer legend corresponds to RGP, *bla* genes, the three efflux pumps and CRIPSR/Cas found in the eighteen GC1 Group 1 genomes in the same loci, indicated with black letters and lines is also shown in the outer circle. The hot spots (HS), RGP, and prophages found in A144 are shown in red letters and lines. Black histogram represents CG content of A144 strain. Regions related to some hotspots (HS) disrupted synteny among GC1 chromosomes which also corresponded to RGP in A144 as follows, RGP2/HS8 (JQSF01000083.1: 1-33700 and JQSF01000082.1: 24920-27366), RGP3/HS12 (JQSF01000043.1: 1 -38248 and JQSF01000071.1: 12398-14538), RGP5/HS15 together with RGP6/HS16 (JQSF01000080.1: 153-15902, JQSF01000022.1, JQSF01000055.1, JQSF01000037.1, JQSF01000003.1, QSF01000040.1, JQSF01000042.1, JQSF01000065.1 and JQSF01000041.1: 1-20291), and RGP7/HS18 that corresponded to AbaR GI (JQSF01000053.1: 1-8092, JQSF01000058.1, JQSF01000063.1: 797-1612, JQSF01000054.1: 253-2358, JQSF01000030.1: 448-21672, and JQSF01000084.1: 23-13490).

Comparative analysis between A144 and A155 chromosomes revealed as expected that they had high degree of synteny between them (Supplementary Figure 1). Besides, a high degree of synteny among all GC1 Group 1 genomes was found including D36, which belongs to another lineage within GC1 epidemic clone (Supplementary Figure 1). Four regions related to hotspots (HS) (see below) disrupted synteny among GC1 chromosomes; at loci ABAYE1410 to ABAYE1438, ABAYE2053 to ABAYE2054, ABAYE2822 to ABAYE3048 and ABAYE3550 to ABAYE3552 in AYE genome; these regions also showed signs of microevolution identified as Regions of Genomic Plasticity (RGP) RGP2/HS8, RGP3/HS12, RGP5 and RGP6 with HS15 and HS16, and RGP7/HS18 that corresponded to AbaR genomic island, respectively (Figure 2) (see below).

### Comparative Analysis of the Resistome of GC1 Genomes

The resistome analysis was carried out in detail for A144 and A155 strains compared with GC1 Group 1 and with the epidemic and sporadic clones of the Outgroup Group 3 genomes (Figure 1, Table 1, and Supplementary Table 3). We found that A144 and A155 showed a multidrug resistant phenotype as defined by Magiorakos et al. (2012) that was in concordance with the findings of antimicrobial resistance determinants by bioinformatics tools (Table 2, and Supplementary Table 3). Both strains harbored almost the same ARG usually associated with the core genome (i.e., *bla*_OXA–__51__–like_ and *ampC*) as well as those acquired by LGT events, but A144 had two copies instead of one of the rifampicin resistance *rpoB* gene as well as for the tetracycline resistance *tet*(A) gene, and one copy of the chloramphenicol resistance *catA1* gene (Supplementary Table 3). The only phenotypic difference related to the presence of these extra genes was a reduced zone diameter for rifampicin in A144 but maintaining the same low MIC (Table 2). Neither A144 nor A155 showed acquired ARG for colistin, fluoroquinolones, trimethoprim, fosfomycin, fusidic acid, glycopeptides, nor rifampicin (Supplementary Table 3).

In regard to the β-lactams resistance genes, the naturally harbored *bla*_OXA–__51__–like_ and *ampC* alleles identified in the GC1 Group 1 genomes were different from those found in the other clones from Outgroup Group 3 (Supplementary Table 3). The *bla*_OXA–__69_ allele (AY458016) was conserved in 17/18 GC1 Group 1 genomes and the remaining strain A388 contained *bla*_OXA–__92_ (WP_059262713.1), in agreement with previous finding of Pournaras et al. (2014). In AYE, *bla*_OXA–__69_ was downstream of IS*Aba1*, which was related to increase β-lactam resistance (Chen et al., 2010). It was not the case for A144 nor A155 which could be in part related to their susceptibility to carbapemens. Analysis of *ampC* showed that A144 and A155 harbored a same novel allele with no IS upstream. A wide variety of *ampC* alleles were in the GC1 Group 1 (mostly alleles 1 and 8) and Outgroup Group 3. The IS*Aba1* and IS*Aba125* were upstream of *ampC* in both groups, with no apparent preference for any allele (Supplementary Table 3).

We found four *bla*_TEM_ alleles in 8/18 GC1 Group 1 genomes and only one *bla*_TEM–__1__–like_ allele with the P3 promoter in the Outgroup Group 3. We identified that A144 and A155 are resistant to sulbactam and harbored *bla*_TEM–__1_ with the Pa/Pb promoters (Table 2, and Supplementary Table 3), which had been shown to exhibit higher strength when compared to P3 (Lartigue et al., 2002; Krizova et al., 2013). Our results suggest that *bla*_TEM–__1_ in A144 and A155 with the Pa/Pb promoters could be involved in the increase of the sulbactam MIC (Table 2). The other three different alleles in GC1 Group 1 genomes were *bla*_TEM–__19__–like_ with P3, which is the weakest promoter and was related to sulbactam susceptibility (Lartigue et al., 2002; Krizova et al., 2013). The other β-lactams resistance genes that we detected in GC1 Group 1 genomes were *bla*_OXA–__10_ (1/18), *bla*_OXA–__23_ (7/18), *bla*_OXA–__235_ (1/18 with 5 copies), *bla*_VEB–__1_ (1/18), *bla*_GES_ (1/18), *bla*_PER–__7_ (1/18), and *bla*_NDM–__1_ (1/18). Of those, we also found *bla*_OXA–__23_ in Outgroup Group 3.

Regarding rifampicin resistance genes, we detected the acquired ARG *arr-2* in two (2/18) GC1 Group 1 genomes and it was absent in Outgroup Group 3 (Supplementary Table 3). A144 was the only strain studied that harbored two copies of the most common *rpoB* allele (Giannouli et al., 2012). Two other GC1 Group 1 genomes and two from Outgroup Group 3 had different alleles of *rpoB* (Supplementary Table 3). Concerning the fluoroquinolone resistance, we found that the Plasmid Mediated Quinolone Resistance genes (PMQR) were absent in the GC1 Group 1 genomes and the Outgroup Group 3. Noteworthy, we detected the mutations in the QRDR of *gyrA* and *parC* enough to predict fluoroquinolone resistance (Ostrer et al., 2019), specifically for ciprofloxacin resistance (GyrA81) in 15 (15/18) and for ciprofloxacin plus levofloxacin (GyrA81-ParC84) in A144 and A155 together with others (11/18) in GC1 Group 1, but only in two and one genomes of Outgroup Group 3 (Supplementary Table 3).

The acquired aminoglycoside resistance genes that we identified in A144 and A155 were the *aac(6*′*)-Ib3* gene cassette located in a class 1 integron within the AbaR GI, and Δ*aac(3)-IIa* and *aph(3*′*)-Ia* located in transposons (Vliegenthart et al., 1989; Arduino et al., 2012) (AN: X60321, X51534, X62115, respectively; Supplementary Table 3). The respective Δ*aac(3)-IIa* genes from A144 and A155 lacked the last 59 bp of the 3′ end of the gene, and they were adjacent to *bla*_TEM–__1_ surrounded by IS*26* as previously described in plasmid pAB35063_a (MK323042.1) (Supplementary Table 4). In total, 14 (14/18) GC1 Group 1 genomes and two in Outgroup Group 3 showed at least one acquired aminoglycoside resistance gene (Supplementary Table 3). A deeper analysis of *aph(3*′*)-Ia* revealed that the same variant surrounded by IS*26* and IS*15DI* was also found in AYE, AB0057, A85, A388, AR_0083, DA33382, NCTC 13421 and NIPH527 GC1 Group 1 genomes at the same locus in A144 and A155 genomes.

Regarding sulfonamide resistance, we detected *sul1* in 15 (15/18) and *sul2* in 4/18 GC1 Group 1 genomes (Supplementary Table 3). In A144 and A155, *sul1* was within a class 1 integron.

The tetracycline resistance genes *tet*(A) (11/18) and *tet*(G) (1/18) were identified only in GC1 Group 1 genomes.

The phenicol resistance gene *catA1* was found in eight GC1 Group genomes with 99.85% of identity compared to the one identified in A144 (Supplementary Table 3).

The *cmlA*, *dfr* and genes related to macrolides, lincosamides and streptogramines resistance (MLS), the *mph(E)* and *msr(E)* genes were found in some GC1 Group 1 and Outgroup Group 3 while they were not detected in A144 nor in A155 (Supplementary Table 3).

Moreover, the presence of class 1 and class 2 integrons was analyzed. The *intI1* gene was identified in 13 out of 18 GC1 Group 1 genomes and in a total of 56 from the 106 genomes of GC1 Groups 1 and 2 (Figure 1, and Supplementary Tables 3, 5). The *intI2* gene was found in seven out of the 106 genomes of GC1 Groups 1 and 2, of which only strain AR_0083 was from GC1 Group 1 (Figure 1, and Supplementary Tables 3, 5).

**FIGURE 3:**
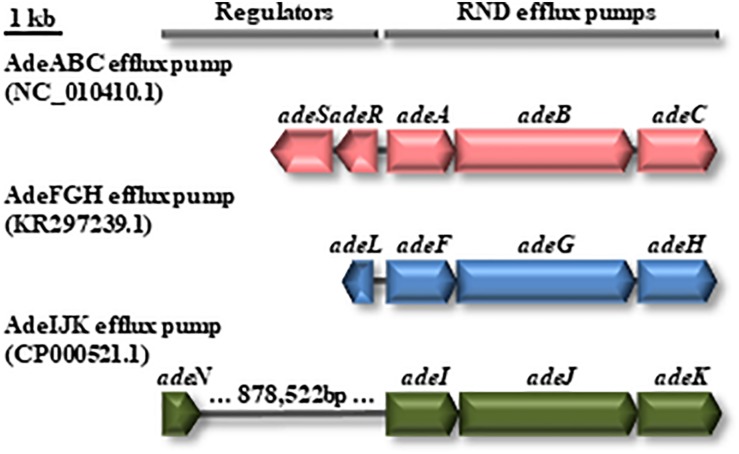
Genetic organization of the AdeFGH, AdeFGH and AdeIJK RND efflux pumps and their regulatory genes. Reference sequences: *adeABC* and *adeRS*, NC_010410.1 (coordinates 1883328-1891105); *adeFGH* and *adeL*, KR297239.1; *adeIJK* and *adeN*, CP000521.1 (coordinates 3171871-3177761 to 2292697-2293350).

The relevance of three resistance-nodulation-cell division-type efflux pumps (AdeABC, AdeIJK, and AdeFGH; Figure 3) in antimicrobial resistance in *A. baumannii* has been highlighted (Marchand et al., 2004; Fournier et al., 2006; Coyne et al., 2010, 2011; Rosenfeld et al., 2012). Here, we investigated their variability in GC1. We found that A144 and A155 shared the same alleles for the three efflux pumps and that all GC1 Group 1 and Group 2 genomes contained these three efflux pumps with different levels of identity for each gene (Figure 1, and Supplementary Tables 3, 6). The complete AdeABC efflux pump genetic structure includes the *adeA*, *adeB*, *adeC* and their regulatory genes, which encode the two-component system AdeRS (*adeR* and *adeS*) (Coyne et al., 2010). None of the *adeR* or *adeS* alleles exhibited the T153M or P116L mutations previously associated with an MDR phenotype in the 106 GC1 genomes (Marchand et al., 2004; Fournier et al., 2006). The *adeABC* and *adeRS* genes were surrounded by ABAYE1818 and ABAYE1824 which encoded hypothetical proteins in AYE. This genetic context was in 17 out of the 18 GC1 Group 1 genomes, except in strain 9102, where ABAYE1818 was also lost together with part of the efflux pump genes described above. Apart from the difference in this last strain, the other GC1 Group 1 genomes contained these genes in the same location as a module; in the case of D36 invasion of IS*Aba1* was identified between *adeR* and *adeA* suggesting a recent acquisition. Interestingly, from the Outgroup Group 4 comprising 2956 non-GC1 draft genomes, *adeC* was absent in 615 draft genomes; even 64 draft genomes lacked the complete *adeABCRS* genes (Supplementary Table 6). Concerning the *adeFGH* efflux pump genes and the *adeL* regulator (Coyne et al., 2010), we found they were within the same loci surrounded by ABAYE1169 and ABAYE1178 which encoded a putative exported protein and a histidine transport system permease protein, respectively. The four genes related to this efflux pump were present in the 106 G1 Group 1 and 2 and also in Outgroup Group 3 genomes, except for Naval-13 and A118 (Figure 1, and Supplementary Tables 3, 6). From the 2956 non-GC1 draft genomes of the GC1 Outgroup Group 4, *adeFGHL* genes were absent in 2 genomes (Supplementary Table 6). The third efflux pump analyzed in this study was the *adeIJK* efflux system which is controlled by a TetR regulator namely *adeN* (Coyne et al., 2010, 2011). GC1 Group 1 genomes showed the *adeN* gene without insertions, and they shared the same allele with 11SNP compared to CP000521.1 except both A144 and A155 which had an extra point mutation (Supplementary Table 3). It was reported previously that the IS*Aba1* disruption of *adeN* in *A. baumannii* PKAB07 eliminated the *adeN* repression of AdeIJK triggering uncontrolled expression of genes of the *adeIJK* operon (Rosenfeld et al., 2012); this event was not found in GC1 Group 1 and 2 genomes. We also observed that in GC1 Group 1 the *adeIJK* efflux pump module was located in the same genetic context flanked by ABAYE0745 and ABAYE0749, as well as the *adeN* regulator gene surrounded by ABAYE1571 and ABAYE1573. Amongst the 2956 non-GC1 draft genomes of the GC1 Outgroup Group 4, the *adeIJK* genes were not complete in 3 genomes, while its regulator gene, *adeN*, was not identified in ten genomes (Supplementary Table 6).

Noteworthy, *adeA* and *adeI* were the only genes that showed 100% identity in GC1 Group 1 and 2, with the exception for *adeA* and *adeRS* genes that were deleted or lost in strain 9102, respectively, probably due to genetic rearrangements (Figure 1, and Supplementary Tables 3, 6). Unlike the other genomes studied here from GC1 Group 1 and 2 and Outgroup Group 3, Naval-13 and A118 lacked the 3 major resistance-nodulation-cell division-type efflux pumps of *A. baumannii* as well as their regulators (Figure 1, and Supplementary Tables 3, 6). Most non-GC1 from Outgroup Group 3 suffered the loss of different *ade* features (Figure 1, and Supplementary Table 3).

The 106 GC1 genomes from Groups 1 and 2 maintained intact the three efflux pumps, except for the partial deletion of *adeABC* efflux pump in strain 9102 (Supplementary Tables 3, 6). As a whole the deep analysis of the three resistance-nodulation-cell division-type efflux pumps in *A. baumannii* revealed that the deletion and loss of some genes affected non-GC1 genomes at different extents, evidencing events of genomic loss mostly in the *adeABC* efflux pump.

### Mobile Genetic Elements Found in GC1 Strains

A wide variety of mobile genetic elements (MGE) associated with the mobilome were identified in GC1 Group 1. We found that the type and the copy number of the IS varied among all the genomes of GC1 Group 1. A144 and A155 shared IS*1R*, IS*6100*, IS*Ppu12*, IS*26* or IS*15DI* (not possible to specify with the sequence available data), and Tn*3*; in A144 we also identified ten IS*Aba125*, five IS*26*, while in A155 we detected three IS*Aba125*, and one IS*Aba125-*related (Supplementary Table 7). It is interesting to note that in A144 we identified a deletion of 1,521 bp within the AbaR GI compared to AYE and AB0057 which resulted in the loss of the 5′ end of a transposase (ABAYE3582) adjacent to an IS*26*, rendering a structure compatible to IS26/IS*15DI*-*tnpR*-Δtnp-IS*26* (AN: JQSF01000054.1). This arrangement was absent in other genomes of the GC1 Groups 1 and 2. A144, A155, AYE, A1, AB0057, A85, NIPH 527, and NIPH 290 shared four IS (IS*1R*, IS*26*, IS*6100* and IS*Ppu12*-related) with different degree of identity among the genomes (from 97 to 100%), which belong to the IS families IS*1*, IS*6*, and IS*L3*, respectively. Most IS copies were found at different loci (Supplementary Table 7).

The IS*6100* found in A144, A155, AYE, A1, AB0057, A85, A388, 11W359501, NIPH 527, and NIPH 290 genomes was located within the same loci in the AbaR GI close to a *mer* module (*merA*, *merC*, *merD*, *merE*, *merP*, *merR*, *merT* genes) between ABAYE3607 and ABAYE3610. In addition, this IS was found related to indels from one side and different insertions or inversions on the other side. IS*6100* was found inverted in A144 and A155 when compared to AYE, evidencing events of microevolution.

As expected, IS*26* and IS*Ppu12*-related that are usually find within the AbaR0-type GI were detected in 14 and 15 out of 18 of GC1 genomes, respectively, evidencing that their absence in some strains was related to genomic reduction or to the absence of the AbaR0-type GI (see below) (Supplementary Table 7).

While six IS (IS*Aba125*, IS*Aba1*, IS*Aba2*, IS*Aba12*, IS*26*, and IS*Ppu12*) were shared by GC1 Group 1 and Outgroup Group 3, 16 IS (IS*1R*, IS*aba10*, IS*Aba26*, IS*Aba14*, IS*10A*, IS*18*, IS*aba13*, IS*15DI*, IS*15DII*, IS*6100*, IS*1396*, Tn*3*, IS*1006*, IS*Vsa3*, IS*Ec29*, and IS*Ec28*) were detected solely in GC1 Group 1 isolates (Supplementary Table 7). On the contrary, three IS identified in Outgroup Group 3 (IS*Aba18*, IS*17* and IS*Aba11*) were not detected in GC1 Group 1 nor in Group 2 draft genomes, while IS*Vsa3* was the only identified in Outgroup Group 3 which was not detected in GC1 Group 1 but in Group 2 draft genomes (7/106). It was curious that AB307-0294 (GC1, Lineage 1) and A118 (sporadic clone) did not harbor any IS (Supplementary Tables 7, 8). These results suggest that although IS may be relevant in most strains, they are not essential for *A. baumannii* survival.

With our set of genomes, prophage analysis showed that the strains from GC1 Group 1 had 11 different putative prophages (Table 3, and Supplementary Figure 2). Interestingly, one of these prophages, the putative prophage YMC/09/02/B1251_ABA_BP (AN: NC_019541.1) was found complete in A144 and with variable lengths in the remaining 17 GC1 genomes assessed including A155 (Table 3). A144 contained a second deleted copy of this prophage (Table 3). We searched for this prophage in GC1 Group 2 and Outgroup Group 3. We found several complete and incomplete copies in every genome of the 88 *A. baumannii* strains from GC1 Group 2 (Data not shown). Four out of five genomes from the Outgroup Group 3 has at least one copy of this prophage (Table 3), evidencing its wide dissemination not only in GC1 but also in several clones of *A. baumannii*. When we investigated the sites of insertion of the prophage YMC/09/02/B1251_ABA_BP in GC1 Group 1, we found several locations except three patterns for some genomes. In AYE, D36, AR_0083, and 9102 the prophage was flanked by the same genes, a cell division gene *zapA* (ABAYE2682) and the 23S rRNA (ABAYE2761) gene (Supplementary Table 9). In one of the prophages found in A155, as well as in AB307-0294, the prophage was flanked by the same cell division gene *zapA* (ABAYE2682) and gdhA_2 gene (ABBFA_02560 locus from AB307-0294) (Supplementary Table 9). Thirdly, in A1, AB0057 and A85 the prophage was flanked by the same genes *aroP* (ABA1_03000) and chaperone *hsp31* gene (ABA1_03077) (Supplementary Table 9). In the case of A144, the prophage was flanked by a putative signal peptide (ABAYE2757) and the same chaperone *hsp31* gene (ABA1_03077). Interestingly both copies of the prophage in A144 and A155 have different insertion sites (Supplementary Table X9), evidencing a trade-off between maintenance of this prophage probably acquired before diversification of GC1, and great processes of microevolution, deletions and insertions. Previously, it has been described the dissemination of *Acinetobacter* ACICU prophage 3 in 151 genomes of *A. baumannii* (Chan et al., 2015). When we searched for prophage 3 in GC1 Group 1 and 3, we found that it was not detected in A144 nor in A155 but present with different lengths in D36, AB0057, A85, A388, DA33382, 11W359501, NCTC13421, and NIPH 290 genomes (Table 3).

**TABLE 3:** Prophages found in GC1 Group 1 and outgroup group 3 genomes.

**Phage**	**Strain**	**Size (bp)**
*Acinetobacter* YMC/09/02/B1251_ABA_BP (NC_019541.1)	A144	41,400; 123,300
	A155	17,800; 68,000
	AYE	56,900
	D36	60,000
	A1	42,800
	AB307-0294	22,500
	AB5075-UW	22,600
	AB0057	27,100; 52,800
	USA15	57,891
	A85	22,275; 57,891; 52,826
	A388	56,058; 23,332
	AR_0083	53,135; 56,637
	DA33382	27,147; 58,967; 49,779
	9102	56,197
	11W359501	91,575; 22,641; 50,064
	NCTC13421	22,641; 33,306
	NIPH 527	44,365
	NIPH 290	49,616
	ACICU	24,700; 53,600
	A118	21,000
	AB33405	62,500; 86,200
	ATCC 17978	64,500
*Mannheimia* vB_MhM_3927AP2 (NC_028766.1)	A144	39,100
	A155	39,100
	D36	36,200
	AR_0083	39,265
*Bordetella* BPP-1 (NC_005357.1)	A144	44,100
	A155	43,700
*Pseudomonas* phi CTX (NC_003278.1)	AYE	34,100
	AB5075-UW	36,900
	USA15	34,024
	A388	20,962
	A85	34,024
	DA33382	34,025
	NIPH 290	33,497
*Pelagibacter* HTVC010P (NC_020481.1)	AYE	35,400
*Psychrobacter* Psymv2 (NC_023734.1)	D36	36,400; 57,800
	Naval-13	54,100
	AR_0083	30,576
*Cronobacter* ENT39118 (NC_019934.1)	D36	20,500
	11W359501	22,507
*Acinetobacter* AP22 (NC_017984.1)	AB5075-UW	27,100
	AB0057	34,600
	NCTC13421	34,689
*Staphylococcus* SPbeta-like (NC_029119.1)	AB0057	43,200
*Pseudomonas* F116 (NC_006552.1)	AB0057	16,000
*Salmonella* vB_SosS_Oslo (NC_018279.1)	AB0057	22,200
	A85	22,274
	DA33382	22,274
	NCTC13421	33,306
*Acinetobacter* ACICU prophage 3 (CP000863.1: ACICU_02140-ACICU_02234)	D36	62,720
	AB0057	68,945
	A85	68,984
	A388	69,231
	DA33382	69,628
	11W359501	62,780
	NCTC13421	69,538
	NIPH 290	70,118
*Acinetobacter* vB_AbaS_TRS1 (NC_031098.1)	Naval-13	54,000
	A118	40,749
	AB33405	48,400
	ATCC_17978	34,900
*Enterobacteria* phage mEp235 (NC_019708.1)	AB33405	44,800
*Erwinia* vB_EamM_ChrisDB (NC_031126.1)	AB33405	7,300

Also, a total of 27 plasmids were found in thirteen out of the eighteen GC1 Group 1 genomes (Supplementary Table 4). AB307-0294, 11W359501, NCTC13421, NIPH 527 and NIPH 290 strains did not harbor any plasmid or replicon. The search of replication initiation proteins previously described (Carattoli et al., 2014; Hamidian and Hall, 2018a; Salto et al., 2018), revealed the predominance of the Rep_3 superfamily (17/27) in the GC1 Group 1 genomes (Supplementary Table 4). It was not possible to identify any replicases in eight replicons, suggesting that they may have other replication mechanisms as previously found in other *A. baumannii* isolates (Salto et al., 2018). The putative plasmids from A144 and A155 had 100% of query cover and 100% identity with *rep_3* from pIH18, which has been recently identified in nosocomial *A. baumannii* isolates from Argentina (Salto et al., 2018). The rep_3 replicase related genes were also spreading in GC1 Group 3 genomes. The RepAci1 that also belongs to Rep_3 Superfamily (ALJ89812.1) was identified in several GC1 plasmids around the world but not in A144 and A155 nor in a previous study from Argentina (Salto et al., 2018) evidencing a different pattern of plasmid dissemination.

Plasmids or putative extrachromosomal replicons ranged from 1,967 bp (p3AB5075, NZ_CP008709.1) to 98,301 bp (pUSA15_1, NZ_CP020594.1). Ten out of the 27 plasmids or putative extra chromosomal replicons carried ARG, whereas p1AB5075 (NZ_CP008707.1) and pD36-2 (NZ_CP012954.1) possessed class 1 integrons with different gene cassettes arrays (Supplementary Table 4). Several IS such as IS*Aba125*, IS*Aba2*, IS*Aba3*, IS*Aba5*, IS*1*, IS*6*, IS*30*, IS*L3*, Tn*4352*:IS*Aba1*, Tn*501*/Tn*1696*, and IS*Aba32* were found in eight plasmids which have related sequences to *rep_3* gene (Supplementary Table 4), evidencing the important role of this family of replicases for the acquisition of IS due to the flux of plasmids by events of LGT by GC1 strains.

### Genomic Analysis and Maintenance Along Time of the AbaR0-Type Genomic Island Identified in A144 and A155 Strains

Sixteen out of eighteen GC1 genomes harbored an AbaR GI inserted in the *comM* gene, except AB307-0294 (Holt et al., 2016) and 9102 that possessed a complete *comM* gene. A144 and A155 genomes contained an AbaR0-type backbone GI, with the typical complete *intI1* gene (Hamidian et al., 2016). The remaining GC1 Group 1 strains from lineage 1 carried AbaR0 or AbaR3-types GIs (Hamidian et al., 2014; Holt et al., 2016; Hamidian and Hall, 2018b). Many of them showed deletions in their structure caused by IS*26* (Data not shown), probably due to either recombination events between duplicate copies of *sul1* or Tn*6018*, or by gene cassette addition or replacement as previously described (Holt et al., 2016; Hamidian and Hall, 2018b).

**FIGURE 4:**
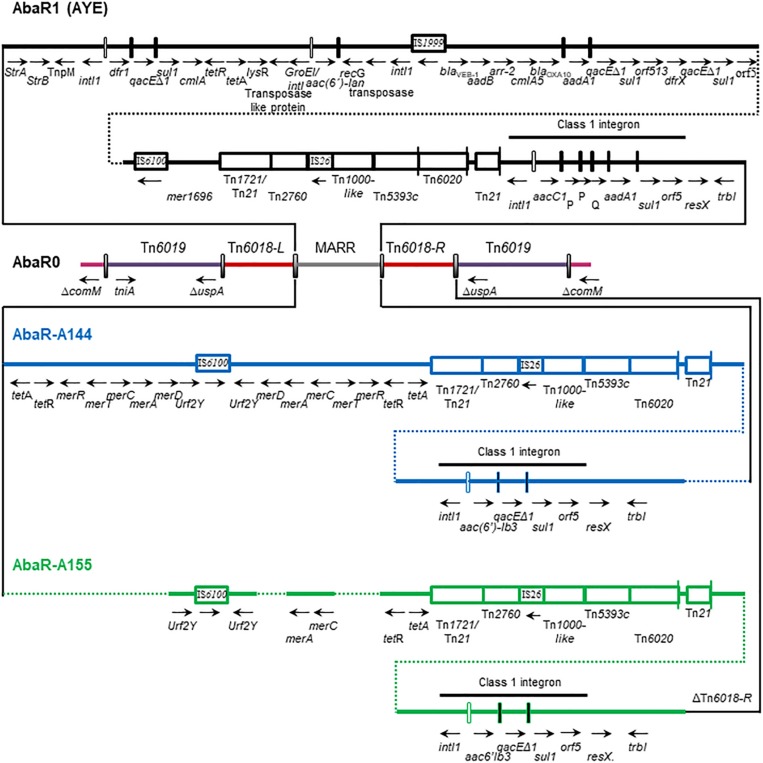
Representation of the AbaR0-like GI found in *Acinetobacter baumannii* AYE, A144 and A155 strains. The *comM* gene is interrupted by the insertion of the AbaR0-like GI. The region of multidrug resistance (MARR) is detailed, in this area we found variations in relation with the AbaR type GI previously reported.

The AbaR0-type GI from A144 contained all the core modules, including Tn*1721*/Tn*21*, Tn*1000*-like, Tn*539*3, Tn*6020* and Tn*21* (Figure 4). In addition, it harbored the transposon Tn*2760* found in AbaR3, but lacked transposon Tn*2*. We also found a variant of multidrug-resistance regions described previously with a change in the module of the Tn*1696* transposon, which presented two copies of *mer* genes flanking the IS*6100* sequence (Figure 4). The class 1 integron found in A144 and A155 had the genetic platform IS*26*-Tn*21*-*intI1*-*aac(6*′*)-Ib*-*qacE*Δ1-*sul1*-orf5. The presence of *aac(6*′*)-Ib* within the variable region of a class 1 integron in an AbaR0-type GI is unusual since it commonly contains the array *aacC1-*orfP-orfP-orfQ*-aadA1* (Kochar et al., 2012; Hamidian and Hall, 2018b). Downstream of orf5 we found the genes *resX* and *trbI*, as previously described (Kochar et al., 2012). As a difference with A144, the AbaR0-type GI from A155 lacked the module carrying both *mer* operons and Tn*6018*-R (Figure 4). The maintenance of the AbaR0-type GI from A144 and A155 strains in the absence of antibiotic pressure was evaluated in three independent experiments after serial subcultures for 30 days by PCR using specific primers for detecting an invasion at the *comM* gene. No loss of the AbaR0-type GI from A144 nor A155 genomes was observed. This experiment evidenced that this GI was stable throughout time at least over one month.

**FIGURE 5:**
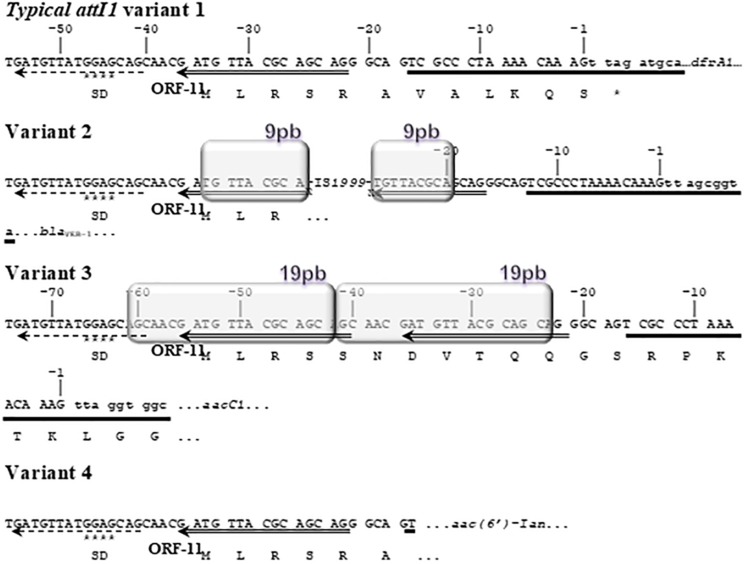
Comparison of the *attI1* recombination sites found in the GI of A144, A155 and AYE. The variant 1 is the typical *attI1* site. Variant 2 shows the insertion of the IS1*999* and a 9 bp duplication of a portion of the *attI1* site. Variant 3 found in the three genomes, shows a 19 bp duplication. Variant 4 has a deletion of the 3′ end of the *attI1* site. The characteristic regions of the *attI1* site are marked as follows: Direct Repeat 2 (DR2), broken-line arrow; Direct Repeat 1 (DR1), double-line arrow; simple site, horizontal line; Shine-Dalgarno (SD) sequence identified for the orf-11, stars. The predicted sequence of the orf-11 is shown. The duplications are depicted in boxes. The gene cassette next to each *attI1* variant is indicated and the corresponding initial nucleotides are shown in lower case. The sequences of the variants 1 to 4 of the *attI1* site found in AYE correspond to the following coordinates in CU459141.1: 3.677.401-3.677.465 bp, 3.661.663-3.663.064 bp, 3.624.336-3.624.419 bp and 3.668.061-3.68.101 bp, respectively.

We further analyzed the *attI1* sites of the class 1 integrons found in the GI of A144 (JQSF01000046.1, contig 2 coordinates 1992-2068) and A155 (JXSV01000033.1, contig 33 coordinates 1933-1857). Both recombination sites were identical to each other and surprisingly to only one *attI1* site when compared to those found in AYE. Three complete class 1 integrons in the AbaR1-type GI had been described in AYE strain (Fournier et al., 2006). When doing this study, we identified a fourth *attI1* site with a deleted *intI1* gene (see below). Interestingly, the four *attI1* sites harbored different variants (Figure 5). We found the typical *attI1* site, that we referred here as variant 1 in the class 1 integron that harbored the *dfrA1* gene cassette in the variable region. The variant 2 was found in the *attI1* site associated to the gene encoding a fusion protein *GroEL/intI1*, with the *bla*_VEB–__1_–*aadB*-*arr-2*-*cmlA5*-*bla*_OXA–__10_-*aadA1* gene cassette array. This *attI1* variant was invaded by the IS*1999*, which additionally generated a 9 bp duplication. The third class 1 integron contained the variant 3 of the *attI1* site and the *aacC1-*orfP-orfP-orfQ-*aadA1* gene cassette array; this variant showed 100% identity with that found in the GI of A144 and A155. This variant contained the insertion of 19 bp in tandem (Wohlleben et al., 1989) at positions -24 and -23 of the typical *attI1* site and it was also found in 11 out of 18 GC1 Group 1 genomes (A144, A155, AYE, A1, AB0057, A85, A388, DA33382, 11W359501, NCTC13421, and NIPH 290) and in 59 genomes from GC1 Group 2, showing that it was present in at least 66% genomes of this clone (70/106). The GCF_003325575.1_ASM332557v1 genome was the only one from GC1 with two copies of this variant. Also, this *attI1* variant was present in ACICU from Outgroup Group 3 genomes (1/5) and in 1,104 genomes (1,104/2956, 31 of which harbored up to three copies of this variant) in Outgroup Group 4 (data not shown). Finally, the fourth *attI1* site in AYE that we identified in this work was the variant 4, which showed a deletion at the 3′ end of the *attI1* site, from position-16 to the end of the site (Figure 5) linked to a second *GroEL/intI1* fusion that contains the first 301 bp of the *intI1* gene. This deleted class 1 integron possesses the *aac(6*′*)-Ian* gene cassette in the variable region that we have previously found as unique gene in AYE compared to other GC1 Group 1 and 2 genomes. It is likely that the novel gene cassette *aac(6*′*)-Ian* has been acquired by variant 4 of the *attI1* site due to an active IntI1 provided *in trans* by other complete *intI1* genes.

### Genomic Diversification by LGT of GC1 Strains

Since *A. baumannii* has a large pangenome with diverse gene traits that suggests frequent LGT events, we evaluated the presence of RGP including GI, and their potential association with hotspots of recombination (HS) as defined previously (see Materials and Methods). Seven RGP were identified in A144. Two of them, the RGP1 and RGP4 were detected in the 106 GC1 Group 1 and 2 genomes but the Outgroup Group 3 (Table 4, and Supplementary Table 6). Interestingly, six of them were found in A155 (RGP1, 2, 3, 4, 6, and 7) and four of them were found in AYE (RGP1, 4, 6, and 7).

**TABLE 4:** RGP found in A144 when compared with the CG1 Group 1 genomes.

**RGP**	**Description**	**# Genes**	**A144**	**AYE**	**A155**	**AB307-0294**	**AB0057**	**D36**	**A1**	**AB5075-UW**	**USA15**
RGP1	Mostly ribosomal genes	29	A144_00407-A144_00435	ABAYE0406-ABAYE0434	A155_00395-A155_00423	CTY05_00431-CTY05_00459	AB57_RS17820-AB57_RS17695	AN415_RS02430-AN415_RS02570	ABA1_03327-ABA1_03355	ABUW_ RS01985-ABUW_ RS02125	B7L41_RS18650-B7L41_ RS18790
RGP2	Mostly genes enconding hypotetical proteins, except for TraR and a DNA-binding protein	49	A144_01401- A144_01449	Not found	A155_01316-A155_01365	Not found	Not found	Not found	Not found	Not found	B7L41_RS13320-B7L41_ RS13560
RGP3	Mostly genes enconding hypotetical proteins, except XerC and a HTH regulator	45	A144_01998-A144_02042	Not found	A155_01909-A155_01952	Not found	Not found	Not found	Not found	Not found	Not found
RGP4	Includes HTH regulators	8	A144_02125-A144_02132	ABAYE2146-ABAYE2153	A155_02036-A155_02043	CTY05_01956-CTY05_01963	AB57_RS08870-AB57_RS08840	AN415_RS10955-AN415_RS10985	ABA1_01650-ABA1_01657	ABUW_ RS11280-ABUW_RS11310	B7L41_RS09985-B7L41_ RS10015
RGP5	All genes encodng hypoteticaproteins	11	A144_02806-A144_02816	Not found	Not found	Not found	Not found	AN415_RS13755-AN415_RS13805	Not found	Not found	B7L41_RS14855-B7L41_ RS14890
RGP6	Includes a gene encoding an ssDNA/RNA exonuclease	6	A144_02920-A144_02925	ABAYE2883-ABAYE2888	A155_02774-A155_02779	CTY05_02675-CTY05_02680	AB57_RS05065-AB57_RS05040	AN415_RS14490-AN415_RS14520	ABA1_00933-ABA1_00938	Not found	B7L41_RS06615-B7L41_ RS06640
RGP7	AbaR GI	52	A144_03814-A144_03865	ABAYE3551-ABAYE3668	A155_03417-A155_03461	Not found	AB57_RS01660-AB57_RS01230	AN415_03722-AN415_03739	ABA1_00237-ABA1_00296	ABUW_3658-ABUW_3678	B7L41_RS03100-B7L41_ RS03305

**RGP**	**Description**	**# Genes**	**A85**	**A388**	**AR_0083**	**DA33382**	**9102**	**11W359501**	**NCTC13421**	**Phage found in RGP**

RGP1	Mostly ribosomal genes	29	CBI29_RS17600-CBI29_RS17740	A388_00462-A388_00490	AM462_RS17415-AM462_RS17555	DPV67_RS00005-DPV67_RS19215	Aba9102_12825-Aba9102_12965	FIM01_02220-FIM01_02360	DQM71_RS02060-DQM71_RS02200	
RGP2	Mostly genes enconding hypotetical proteins, except for TraR and a DNA-binding protein	49	Not found	Not found	AM462_02365-AM462_RS02605	Not found	Not found	Not found	Not found	*Mannheimia* vB_MhM_3927AP2 (NC_028766.1)
RGP3	Mostly genes enconding hypotetical proteins, except XerC and a HTH regulator	45	Not found	Not found	Not found	Not found	Not found	Not found	Not found	*Bordetella* BPP-1 (NC_005357.1)
RGP4	Includes HTH regulators	8	CBI29_RS08770-CBI29_RS08800	A388_02216-A388_02224	AM462_RS06010-AM462_RS06040	DPV67_RS02970-DPV67_RS03000	Aba9102_01795-Aba9102_01825	FIM01_11750-FIM01_11780	DQM71_RS10890-DQM71_RS10920	
RGP5	All genes encodng hypoteticaproteins	11	CBI29_RS13420-CBI29_RS13455	A388_01316-A388_01322	AM462_RS09115-AM462_RS09155	DPV67_RS07705-DPV67_RS07740	Aba9102_04210-Aba9102_04225	FIM01_06560-FIM01_06625	DQM71_RS06140-DQM71_RS06175	*Acinetobacter* YMC/09/02/B1251_ABA_BP (NC_019541.1)
RGP6	Includes a gene encoding an ssDNA/RNA exonuclease	6	CBI29_RS04950-CBI29_RS04975	A388_02963-A388_02968	AM462_RS09990-AM462_RS10095	DPV67_RS18455-DPV67_RS18480	Not found	FIM01_15120-FIM01_15145	DQM71_RS14685-DQM71_RS14710	
RGP7	AbaR GI	52	CBI29_RS01230-CBI29_RS01660	A388_00226-A388_03612	AM462_RS13330-AM462_RS15430	DPV67_RS15110-DPV67_RS15295	Not found	FIM01_18920-FIM01_19280	DQM71_RS18135-DQM71_RS18565	

The RGP1 harbored a block of 29 genes related to LSU and SSU ribosome proteins (ABAYE0406-ABAYE0434) and its G + C content (43%) differed from the average content of *A. baumannii* GC1 strains (39%). GC1 Groups 1 and 2 harbored the RGP1 with 100% of query cover and most of them with more than 99.96% identity (Table 4, and Supplementary Table 6). The RGP4 contained 8 genes (ABAYE2146-ABAYE2153); one of them was related to a phage integrase gene, and another was a putative repressor related to the TetR family (TetR) which are genes that code for proteins playing an important role in the regulation of tetracycline resistance and other functions (Supplementary Table 10; Ramos et al., 2005; Saranathan et al., 2017). The 106 GC1 genomes harbored the RGP4 usually with more than 97.25% of query cover and most of them with more than 99.98% identity, evidencing as well as RGP1 a high nucleotide conservation among GC1 members (Supplementary Table 6). Twenty-seven RGP1 and 34 RGP4 were also found in other non-GC1 genomes from *A. baumannii* Outgroup Group 4 with 100% query cover and identity (Supplementary Table 6).

Besides the previously described AbaR0-type GI, which corresponded to RGP7, three out of seven RGPs were detected as GI by bioinformatics analysis (RGP2, RGP3, and RGP5) (Table 4). These three RGP also contained putative phages denoting a potential DNA mobilization to other strains. RGP2 was identified in A144, A155, USA15 and AR_0083 strains while RGP3 was identified only in A144 and A155 (Figure 2). A144 and A155 contained phage *Mannheimia* vB_MhM_3927AP2 (AN: NC_028766.1) in RGP2 and phage *Bordetella* BPP-1 (AN: NC_005357.1) was identified in RGP3. A144 and D36 also shared RGP5 (Table 4), which contained phage *Acinetobacter* YMC/09/02/B1251_ABA_BP (AN: NC_019541.1). Lastly, RGP6 in A144 contained six genes including two coding for Phage T7 exclusion proteins.

**FIGURE 6:**
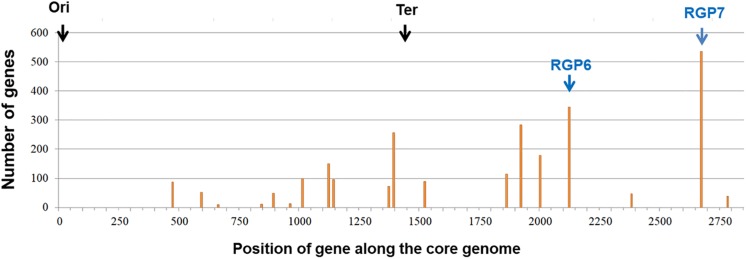
Distribution of hotspots along the core-genome of *Acinetobacter baumannii* GC1 Group 1 genomes. The gene order of *A. baumannii* AYE strain was used as a reference (see section “Materials and Methods”). The bars represent the number of different gene families in all the genomes found between two consecutive genes of the core-genome of GC1 genomes.

On the other hand, it has been previously identified 78 hotspots of recombination (HS) in *A. baumannii* genomes belonging to several lineages (Touchon et al., 2014). Here, we identified 19 HS of recombination along the core-genome of GC1 Group 1 strains using AYE as reference (Figure 6). As previously reported, a concentration of hotspots closer to the terminus of replication and symmetrically distributed around this position (Bobay et al., 2013; Touchon et al., 2014), was also observed in GC1 Group 1 genomes from our study (Figure 6). At the same time, GC1 chromosomes have regions with no signs of genome plasticity suggesting that they are less plastic (Figure 6). Interestingly, some hotspots were related to RGP such as the case of RGP6 (ABAYE2883-ABAYE2888) which contained a 3′-5′ ssDNA/RNA exonuclease gene, a queC_2 7-cyano-7-deazaguanine synthase gene and four hypothetical proteins in A144 corresponding to HS16, and RGP7 which harbors the AbaR0-type GI, corresponding to HS18, evidencing association with LGT events (Figure 6). HS1 in AYE and AB5075-UW was also associated with LGT events since phage related genes were found inserted within the two core genes. HS2 also encoded phage related proteins in D36 and was inserted within two core genes. HS3 to HS6, HS14 and HS15 possessed genes coding for hypothetical proteins; HS7 in AB5075-UW encoded a site-specific integrase, one IS*256* and also a phage-related protein; HS10 in AYE had the transposase IS*Aba1*; HS11 and HS13 had phage-related genes; and HS17 in AYE had *copC* and *copD* genes that belong to a diverse group of periplasmic copper binding proteins. Lastly, as previously detected by Holt et al. (2016), HS19 was found within genes coding for the biosynthesis of exopolysaccharides via the K locus. When we investigated deeply the variability in K locus in our set of 106 genomes from GC1 Group 1 and 2, we found ten gene clusters of K locus (KL1, KL4, KL9, KL12, KL15, KL17, KL20, KL25, KL40 and KL42) with 100% of query cover and more than 97% of identity, and five gene clusters for OC locus (OCL1, OCL2, OCL3, OCL4 and OCL5) which were already described by Holt et al. (2016, Supplementary Table 8). The KL1 locus (31/106) was prevalent amongst GC1 genomes in both lineages (Supplementary Table 11). The OC locus was absent in 9102 (NZ_CP023029.1) complete genome (Supplementary Table 11).

### Identification of Adaptive Immune Systems in GC1 Genomes

We also looked for Clustered Regularly Interspaced Short Palindromic Repeats and their associated Cas proteins (CRISPR-Cas systems). These elements can provide host immunity against bacteriophages and plasmids (Shmakov et al., 2017), which are commonly associated to LGT events (Di Nocera et al., 2011; Touchon et al., 2014). Previously it has been reported that most CRISPR-Cas systems found in the genus *Acinetobacter* corresponded to type I-Fa or Fb elements, where type I-Fb was highly spread in different clonal complexes with a significant vertical evolution including GC1 (Hauck et al., 2012; Touchon et al., 2014; Karah et al., 2015).

*In silico* prediction and analysis showed that A144 and A155 genomes had the identical type I-Fb system found in strain AYE, which consist of 6 *cas* genes (*cas1-cas3-cas8f-cas5f-cas7f-cas6f*). A144 and A155 systems contained each an identical CRISPR array consisting of 53 spacers and repeat sequences (Table 5). This finding supports the clonal relationship between both strains. All 106 GC1 genomes from GC1 Group 1 and 2 had the same *cas* operon (100% of query cover and 99.97%), and their respective CRISPR arrays contained between 45 and 81 spacers adjacent to the same repeat sequence (Table 5). We then analyzed all the spacer sequences from A144 and AYE and we found that most of them had a low degree of complementation to phage related sequences from *Staphylococcus aureus*, *S. epidermidis*, *Klebsiella* spp., *Escherichia* ssp., and other species usually found in nosocomial niches (Supplementary Table 12).

**TABLE 5:** Analysis of CRISPR-Cas system in GC1 genomes.

**Strain**	**Number of spacers**
A144	52
A155	52
A1	50
AB307-0294	45
AYE	59
AB0057	52
AB5075-UW	52
D36	81
USA15	49
A85	34
A388	52
AR_0083	47
DA33382	15
9102	50
11W359501	46
NCTC13421	46
NIPH 527	18
NIPH 290	18

*The 6 *cas1-cas3-cas8f-cas5f-cas7f-cas6f* genes were identified in the eighteen GC1 genomes of this study. All the repeats had the same sequence (5′-GTTCATGGCGGCATACGCCATTTAGAAA-3′).*

Last, we investigated the dissemination of *cas1-cas3-cas8f-cas5f-cas7f-cas6f* (8103 bp) in Outgroup Group 3, Outgroup Group 4 and other genomes from GenBank. Blastn search revealed that the locus was present in 121 out of 2956 *A. baumannii* non-GC1 genomes belonging to different sequence types from Outgroup Group 4 with a nt identity that ranged from 97.42 to 99.89% as previously described (Supplementary Table 6; Karah et al., 2015). Also, the locus was present in isolates from other *Acinetobacter* species such as *A. haemolyticus* TG19602, *A. gyllenbergii* NIPH 230 and in *A. parvus* as previously reported (Touchon et al., 2014; Karah et al., 2015).

### *In vitro* Competition of A144 and A155

To understand the success of GC1 in the nosocomial niche with experimental data, we also investigated both the clonal competition between A144 or A155 versus A118 (sporadic clone) in the absence of antimicrobial pressure.

A144 and A118 showed slight differences in the growth rate. The growth rate of A144 was *r* = 0.015 OD/min with a doubling time Dt = 46.18 min. For A118, the growth rate was *r* = 0.015 OD/min with a doubling time Dt = 46.98 min. Conversely, A155 showed a faster growth in the conditions assayed, with values of *r* = 0.017 OD/min, and a doubling time Dt = 40.98 min.

A144 showed a fitness advantage with an *S*-value *S* = 0.333 ± 0.069 in the fitness assays for the clonal competition between A144 and A118 carried out for 124 generations; with a relative adaptive fitness *F* = 1.333 (133.318%) and a fitness cost *C* = −33.318%. As expected, the clonal competition between A155 and A118 carried out for 140 generations also showed a fitness advantage (C < 15.574%) with an *S*-value *S* = −0.156 ± 0.018, *F* = 1.156 (115.574%) and *C* = −15.574%. The clonal competition assay showed a competitive advantage of the GC1 strains over the sporadic clone in the two pairs (A144/A118 and A155/A118) with the fitness cost *C* > 10% and a statistically significant difference (*P* = 0.00001, P < 0.05). Previous reports described that the greater the difference in growth rate between two strains, the greater is the bacterial burden difference over time (Guo et al., 2012); nevertheless, differences i.e., in antimicrobial resistance and virulence factors should be under consideration altogether. Here, the difference between this two pairs of strains (A144/A118 and A155/A118) showed the relevance of even small genomic modifications in the fitness of a strain (A144 and A155) when competes with another.

Regarding virulence factors, *A. baumannii* usually encodes a type VI secretion system (T6SS), which can be used to kill competitors (Weber et al., 2013). It is likely that the presence of this system may be associated to the survival in the nosocomial niche (Fitzsimons et al., 2018). Here, we searched the genes coding for the proteins critical for T6SS operation (Weber et al., 2016; Repizo et al., 2019) in Groups 1, 2, 3 and 4, as well as *tetR1* and *tetR2* repressors in Groups 1, 2 and 3 (Supplementary Table 13). The genes coding for the core proteins of this system were present in A144 and A155 and in all genomes from GC1 Group 1 and 2 but NIPH290, GCF_000369185.1, GCF_000369325.1, GCF_004347305.1_ASM434730v1 and GCF_006494215.1_ASM649421v1 where the T6SS was partially lost (101/106) (Supplementary Table 13). The *tetR1* and *tetR2* repressor genes were not detected in GC1 Group 1 and 2 but GCF_001512215.1_9179_4_6 and GCF_006492525.1_ASM649252v1 genomes that harbored *tetR2* (*tetR2* = 2/106). When we searched in the Outgroup Group 3, we identified the complete T6SS in epidemic clones and partially deleted in A118 which belongs to a sporadic clone (Supplementary Table 13). Interestingly, both repressor genes *tetR1* and *tetR2* were only present in Naval-13. In Outgroup Group 4, which includes *A. baumannii* non-GC1 strains, we found that the 17.46% of draft genomes had partially or complete lost components of the T6SS (2440/2956) (Supplementary Table 13).

Taken together, the fitness of A144 and A155 over sporadic clones, and the finding of complete T6SS in almost all GC1 strains compared to non-GC1 genomes, suggests that these features can be related to the epidemic behavior of the so-called clones that can rapidly displaced sporadic clones or strains lacking the T6SS.

## Discussion

Genomic studies in combination with biological analysis led us to identify maintenance along time of genes usually subjected to LGT that may have a crucial role during the evolutionary trajectory of the high-risk clone GC1. The genes from the accessory genome acquired by GC1 strains were mostly grouped in modules along the chromosome and preserved by two adaptation pathways over time. On the one hand, the paradigmatic AbaR GI, the CRISPR-Cas type I-Fb system, as well as two novel regions of genome plasticity (RGP1 and 4), were located within the same loci as “sedentary” modules (Figure 2). In turn, the AbaR GI showed high plasticity evidenced by several signs of microevolution including deletions, inversions and duplications as previously described (Hamidian et al., 2016). We found that even the AbaR0-type GI from our two strains A144 and A155 isolated from the same hospital H1, presented signs of microevolution since A144 isolated 3 years later than A155 had acquired a duplicated *mer* operon and a Tn*6018*-R (Figure 4). In concordance with these results, this region was identified as a hotspot for recombination, corresponding to HS18 in our study (Figure 6). This is in agreement with the fact that, to the best of our knowledge, there are no reports of AbaR GI harboring identical genetic structure in *A. baumannii* strains (Ramírez et al., 2013; Hamidian and Hall, 2018b). Otherwise, our experimental studies revealed that AbaR GI is maintained over time in A144 as well as in A155 without antimicrobial pressure for at least one month. We can assume that there is a balance between conservation and plasticity of particular regions of the accessory genome, as previously described for the core genome regions of *A. baumannii* (Touchon et al., 2014; Hamidian et al., 2016). The other remaining “sedentary” modules, the *cas* genes from the type IF-b CRISPR-Cas system, RGP1, and RGP4, did not show signs of microevolution.

We confirmed the preliminary data reported by Karah et al. (2015), since we found that the *cas* operon from AYE was conserved in all the 106 GC1 genomes, supporting that its acquisition occurred before GC1 clonal diversification (Supplementary Tables 6, 12) but after speciation of *A. baumannii*. It is particularly intriguing the complete identity found between the CRISPR-Cas systems, including the order and identity of 53 spacers of both A144 and A155 strains from the same hospital, considering that they were isolated at distant time points (Table 5). It is highly probable that each strain was exposed to different phages and plasmids; however, there is no evidence of new invasions. Since the same *cas* genes from the type IF-b CRISPR-Cas system were found in other GC1 chromosomes and also in other species of the genus *Acinetobacter* carrying different spacers (Karah et al., 2015; Supplementary Table 12), it is likely that they are functional systems. Thus, it is possible to assume that a tight regulation of these systems may have led to a stable array for A144 and A155. In this regard, it has been reported that phages can encode proteins with anti-CRISPR activity that may inhibit their function (Pawluk et al., 2014; Bondy-Denomy et al., 2015). Further studies are necessary to confirm this hypothesis. This finding and the closeness observed in the phylogenetic tree (Figure 1) suggest that A144 and A155 strains may share a common ancestor from which they adapted and evolved within the H1 nosocomial niche.

Concerning the regions of genomic plasticity, we found that RGP1 and RGP4 were highly conserved in the 106 GC1 genomes and absent in almost all other lineages of *A. baumannii*. Ortholog genes of the RGP1 harboring 29 genes related to LSU and SSU ribosome proteins were previously described as an unusual multisequence alignment block structure with important evolutionary implications (Vishwanath et al., 2004). The implication of the RGP4 that includes a putative repressor protein related to the TetR family which can act on various genes with diverse functions such as biosynthesis, metabolism, bacterial pathogenesis, and response to cell stress (Ramos et al., 2005; Saranathan et al., 2017) remains unclear. The functional role of both RGP1 and RGP4 in GC1 lineage would be an interesting challenge to further investigate.

On the other hand, another module of the accessory genome showed to be “mobile” though present in the 106 GC1 genomes (Table 3). This is the case of the YMC/09/02/B1251_ABA_BP putative prophage which showed to vary in length structure across GC1 genomes (Table 3) and it was located in diverse insertion sites as we identified in this study (Supplementary Table 9). Ten additional prophages were detected in the GC1 Group 1 genomes. The biodiversity of prophages as well as the rearrangements they promote within each genome reflects frequent events of successful phage invasion. Since phages may acquire ORFs named morons (Hendrix et al., 2000), the presence and the mobility of prophage *Acinetobacter* YMC/09/02/B1251_ABA_BP in all 106 GC1 strains denotes it may have an important role in acquisition of accessory genome by LGT in this pandemic clone. In agreement with this, we found also a process of deep microevolution for prophage 3 that is widespread in *A. baumannii* strains (Chan et al., 2015), suggesting that prophages may play an important role for genomic plasticity not only in GC1 but in all the species.

Interestingly, a particular genetic behavior was identified for the IS. They showed a great variability in terms of IS families and copy number, having each GC1 strain different amount of IS in different chromosomal locations. There was no evidence of a sequential acquisition of the IS in the distribution observed among the 18 GC1 Group 1 genomes, indicating that the presence of these genetic elements is likely to be related to the rapid adaptation of the strains to the environment they are exposed to. Hence, they may be involved in niche adaptation in GC1 strains. IS analysis also showed that A144 and A155 shared some IS as a main difference with other GC1 Group 1 strains (Figure 2), reinforcing our phylogeny data (Figure 1) that they share a common ancestor. Other IS, such as IS*26* and IS*Aba1* were frequent in GC1 Group 1 strains and they were identified in both GC1 lineages. Remarkably, the GC1 Group 1 genomes did not share a common IS (Supplementary Table 7). The fact that no IS were detected in AB307-0294 genome supports the hypothesis that IS may have been acquired after the diversification of this clone and/or that IS are easily acquired and lost by GC1 strains. Besides, a great variety of IS were identified in plasmids from GC1 Group 1 genomes suggesting that extrachromosomal replicons, especially plasmids harboring Rep_3 replicases, may contribute to the capture and flux of IS that later could invade the chromosome. The relevance of Rep_3 replicases in the acquisition of IS by the chromosome is reinforced by the fact that this family of replicases were found in different species, families and even phyla as well as in environmental or clinical strains (Salto et al., 2018), which enhances the set of IS diversity that can be captured by GC1 strains.

Concerning our genomic analysis related to antimicrobial resistance, we found that the bioinformatic results matched the multidrug resistant phenotype of A144 and A155 with only slight differences in the ARG content (Tables 2, and Supplementary Table 3). An interesting feature was found by analyzing the naturally harbored β-lactamase genes *ampC* and *bla*_OXA–__51__–like_ in GC1. Both β-lactamase genes are ubiquitous in *A. baumannii* strains (Merkier and Centrón, 2006; Karah et al., 2017). Previously, several alleles of *ampC* were identified (Karah et al., 2017). We found nine *ampC* alleles in the eighteen genomes from our GC1 Group 1. Conversely, *bla*_OXA–__51__–like_ was identified as *bla*_OXA–__69_ in 17/18 GC1 Group 1 genomes except A388 which contained *bla*_OXA–__92_ as previously described (Pournaras et al., 2014). These results suggest different degrees of genomic plasticity for each β-lactam resistance gene. This feature is also supported by the identification of a hotspot of recombination in the *ampC* flanking regions (Holt et al., 2016). It is likely that different genetic behaviors of each β-lactamase gene within the same cell may be a powerful tool to a more successful response to antimicrobial selection in the nosocomial niche. The sulbactam resistance in A144 and A155 may be explained by the presence of the *bla*_TEM–__1_ gene with the Pa/Pb promoters since our results indicated that this gene/promoter combination could be involved in the increase of the sulbactam MIC (Tables 2, and Supplementary Table 3). Additional studies to effectively quantify the level of increase in the MIC for sulbactam remain to be done, but the evidence showed here correlates perfectly with the results from other authors, which showed that that the Pa/Pb promoters were stronger than the promoter P3 (Lartigue et al., 2002; Krizova et al., 2013).

The fluoroquinolone resistance in A144 and A155 and of the GC1 Group 1 (15/18 GC1 Group 1) correlated with the results of Ostrer et al., which stated that it could be predicted based solely on target gene quinolone-resistance mutations for *A. baumannii* and that the primary mutation is followed by either of two mutations in the alternate target in this species ParC88 ← **GyrA81** → ParC84 (Ostrer et al., 2019). Even when most of the GC1 Group 1 genomes showed a predicted fluoroquinolone resistance due to mutation in QRDR, it is remarkable the absence of PMQR genes in the isolates analyzed here.

By focusing in the antimicrobial resistance adaptation of GC1 to XDR phenotypes, our data evidenced that class 1 integrons were identified in 13 out of 18 GC1 genomes suggesting they may play an essential role for acquisition of mobile antimicrobial resistance. We identified one additional deleted *attI1* site, which may have arisen from deletions and rearrangements of previous complete class 1 integrons within the AbaR GI of AYE. It is likely that this fourth deleted *attI1* was recognized as a secondary site and at the same time it was related to the acquisition of the novel allele *aac(6*′*)-Ian* which has not been found in other isolates. It is likely that ARG cassettes could be captured by a type 1 integron integrase from a complete class 1 integron and inserted in the respective *attI1* site or in secondary sites, which may act as hotspots for active acquisition of mobile antimicrobial resistance in nosocomial niches. In agreement with previous results from our laboratory (Ramírez et al., 2015), only seven class 2 integrons were identified in GC1 Group 1 and 2 strains (*n* = 106), confirming that the prevalence of *intI2* in *A. baumannii* strains from Argentina is related to the emergence of novel singletons and to the abundance of CC113/CC79, which has been the local dominant lineage for several decades (Stietz et al., 2013). On the other hand, the fact that 56 out of 106 GC1 strains harbored an *intI1* gene, suggests a wide dissemination of class 1 integrons in this pandemic clone. Taking together, these results evidence a different epidemiology of multidrug resistant integrons among *A. baumannii* lineages.

Regarding the investigation about the variability of three resistance-nodulation-cell division-type efflux pumps in GC1, we found that the 106 GC1 genomes contained all the genes encoding the AdeABC, AdeFGH and the AdeIJK efflux pumps altogether with their regulators with different levels of identity for each gene, except for the partial deletion of AdeABC in strain 9102 (Supplementary Tables 3, 6). Meanwhile, our study showed that the three efflux pumps are subjected of processes of genomic loss in non-GC1 strains, particularly those related to *adeABC* genes (Supplementary Table 6). In this regard, it has been previously suggested that *adeABC* was subjected to loss and acquisition along time, while the *adeFGH* system is intrinsic to the *A. baumannii* species (Coyne et al., 2010, 2011). Concerning the molecular epidemiology of the *adeABC* genes, it has been shown that this operon is present in ca. 80% (from 53% to 97%) of *A. baumannii* strains and it is associated mainly with clinical isolates since it has not been found in 32 environmental strains (Huys et al., 2005; Chu et al., 2006; Hujer et al., 2006; Nemec et al., 2007; Bratu et al., 2008; Chen et al., 2009; Lin et al., 2009; Srinivasan et al., 2009). Moreover, Ab421 HEIGH-2010 strain as well as other 10 clinical strains of *A. baumannii* belonging to clone ST79/ST924 lacked these genes and were found to display increased invasiveness (Rumbo et al., 2013; López et al., 2017). Taking together the evidence, the *adeABC* genes from *A. baumannii* may be suffering genomic losses resulting in its presence in less than the 90% of the total genomes of the species as also seen in our study (Supplementary Tables 3, 6), suggesting that the *adeABC* efflux pump as part of the core genome could be under consideration. We also have detected a deep process of genomic loss of the T6SS in non-GC1 strains. It is likely that the maintenance along time and continents of complete AdeABC, AdeFGH and the AdeIJK efflux pumps related to antimicrobial resistance as well as the T6SS which is associated to kill competitors (Weber et al., 2013) may contribute to the survival of GC1 in the nosocomial niche. Although the huge pangenome of *A. baumannii* (Touchon et al., 2014; Chan et al., 2015) evidences dynamic processes of loss and gain of genes, the maintenance of some blocks of accessory genome within a lineage suggests that the general idea that all genes acquired by LGT are easily lost, should be analyzed more deeply in biological models.

We identified an essential role of still unknown properties of “mobile” and “sedentary” accessory genome that is preserved over time under different antibiotic conditions and nosocomial habitats having a decisive role in the adaptive success of the pandemic GC1. In fact, it may be associated with the survival under stress conditions of GC1, which is reflected in its perpetuation along time in strains from different continents. At the same time, our data suggests that GC1 is constantly evolving and adapting to novel niches by exposure to a continuous acquisition of IS which may contribute to the instantly adaptation to the changing stresses suffered by the trajectory of GC1 strains. In these processes, plasmids harboring *rep_3* replicases might have an important role for the flux of IS and antimicrobial resistance determinants. Not only genomic plasticity in *A. baumannii* is evidenced by hotspots for recombination, gene duplications, deletions and/or insertions (Touchon et al., 2014; Holt et al., 2016), but also for the maintenance of several modules of accessory genome, such as RGP1 and RGP4, CRISPR-Cas system, AbaR GI as previously found (Holt et al., 2016), mobile prophage YMC/09/02/B1251_ABA_BP as well as the preservation of synteny of genomes belonging to GC1, being these traits pivotal for the success of this high-risk clone. Competition assay of A144 as well as A155 versus A118 without antimicrobial pressure suggested a greater ability of GC1 to thrive over the clones with sporadic behavior, which in conjunction with the presence of a complete T6SS and efflux pumps in almost all GC1 genomes can explain from an ecological perspective the success of this pandemic clone to spread and survive in hospital environments.

## Author’s Note

DC, MQ, and CQ are members of the Carrera del Investigador Científico, CONICET, Argentina. VA and AG are recipients of a CONICET postdoctoral and doctoral and fellowships, respectively. EV received a CONICET doctoral fellowship.

## Data Availability Statement

The raw data supporting the conclusions of this article will be made available by the authors, without undue reservation, to any qualified researcher.

## Author Contributions

DC and MQ contributed to the conception and design of the study. VA, MQ, AG, MR, and EV performed the experimental and/or bioinformatic assays. DC structured the work, wrote and coordinated the drafts of the manuscript and did the final edition. VA, MQ, CQ, and DC wrote sections of the result’s section. All the authors contributed to the analysis of the data, manuscript revision, read, and approved the submitted version.

## Conflict of Interest

The authors declare that the research was conducted in the absence of any commercial or financial relationships that could be construed as a potential conflict of interest.

The reviewer GR declared a shared affiliation with no collaboration, with the authors, to the handling Editor at the time of review.

## Footnotes

1 http://denovoassembler.sourceforge.net

2 https://github.com/tseemann/mlst#mlst

3 http://www.higiene.edu.uy/ddbp/Andres/gtraglia_et_al_2018b_data.html

4 http://pubmlst.org/abaumannii/

5 http://crispr.i2bc.paris-saclay.fr/Server/

6 http://omics.informatics.indiana.edu/CRISPRone/
