# Supplementary material for: Crucial Role of the Accessory Genome in the Evolutionary Trajectory of Acinetobacter baumannii Global Clone 1
Source: Front Microbiol. 2020 Mar 18;11:342. doi: 10.3389/fmicb.2020.00342 (PMC7093585; doi:10.3389/fmicb.2020.00342)
Supplement: Supplementary file 3 [file Table_1.docx]

**Supplementary Table 1. GC1 Group 2 and Outgtoup Group 4.** The Group 2 was composed by 27 genomes as scaffolds and 61 as contigs from GenBank that were identified as GC1. The Outgroup Group 4 was composed by 2407 genomes as contigs and 549 genomes as scaffolds of *A. baumannii* that were identified as non GC1 using the program mlst (https://github.com/tseemann/mlst#mlst).

| **Contigs GC1 Group 2** | **Scaffolds GC1 Group 2** | |
| --- | --- | --- |
| GCF_000241685.1_ASM24168v2 | GCF_000369185.1_Acin_baum_NIPH_527_V1 | |
| GCF_000286615.1_gacin07v1.0 | GCF_000369325.1_Acin_baum_NIPH_290_V1 | |
| GCF_000297595.1_gacin26v1.0 | GCF_000369365.1_Acin_baum_ANC_4097_V1 | |
| GCF_000301435.1_ASM30143v1 | GCF_000623035.1_Acin_baum_R1B_V1 | |
| GCF_000302035.1_ASM30203v1 | GCF_000794125.1_ASM79412v1 | |
| GCF_000304655.1_gacin18v1.0 | GCF_001373535.1_Acinetobacter_baumannii_CHI-45-1 | |
| GCF_000304695.1_gacin17v1.0 | GCF_001373575.1_A._baumannii_CHI-34 | |
| GCF_000309275.1_gacin36v1.0 | GCF_001511875.1_9179_4_76 | |
| GCF_000441955.1_gabv01 | GCF_001511895.1_9179_4_80 | |
| GCF_000453045.1_ASM45304v1 | GCF_001511915.1_7521_8_47 | |
| GCF_000453085.1_ASM45308v1 | GCF_001511935.1_7468_2_60 | |
| GCF_000453105.1_ASM45310v1 | GCF_001511975.1_7468_2_93 | |
| GCF_000453205.1_ASM45320v1 | GCF_001511995.1_7521_8_27 | |
| GCF_000453885.1_ASM45388v1 | GCF_001512055.1_7468_2_74 | |
| GCF_000453945.1_ASM45394v1 | GCF_001512075.1_7521_8_28 | |
| GCF_000454005.1_ASM45400v1 | GCF_001512135.1_7468_2_79 | |
| GCF_000586635.1_MRSN57 | GCF_001512155.1_7521_8_36 | |
| GCF_000586655.1_MRSN58 | GCF_001512175.1_7468_2_75 | |
| GCF_000770605.1_gabab5075v1.0 | GCF_001512195.1_7468_2_95 | |
| GCF_000836625.1_ASM83662v1 | GCF_001512215.1_9179_4_6 | |
| GCF_000969385.1_ASM96938v1 | GCF_001512235.1_7468_2_91 | |
| GCF_001028265.1_gdodcwi06_v01 | GCF_001512255.1_7521_8_39 | |
| GCF_001028315.1_gdodcwi05_v01 | GCF_001549575.1_ASM154957v1 | |
| GCF_001657725.1_ASM165772v1 | GCF_001612415.1_ASM161241v1 | |
| GCF_001874945.1_ASM187494v1 | GCF_001612435.1_ASM161243v1 | |
| GCF_001907145.1_ASM190714v1 | GCF_001612455.1_ASM161245v1 | |
| GCF_002143965.1_ASM214396v1 | GCF_002838005.2_ASM283800v2 | |
| GCF_003020205.1_ASM302020v1 |  | |
| GCF_003020215.1_ASM302021v1 |  | |
| GCF_003020225.1_ASM302022v1 |  | |
| GCF_003020365.1_ASM302036v1 |  | |
| GCF_003020405.1_ASM302040v1 |  | |
| GCF_003020415.1_ASM302041v1 |  | |
| GCF_003185745.1_ASM318574v1 |  | |
| GCF_003325575.1_ASM332557v1 |  | |
| GCF_003325665.1_ASM332566v1 |  | |
| GCF_003627465.1_ASM362746v1 |  | |
| GCF_003711885.1_ASM371188v1 |  | |
| GCF_003948175.1_ASM394817v1 |  | |
| GCF_003948695.1_ASM394869v1 |  | |
| GCF_003948995.1_ASM394899v1 |  | |
| GCF_003949635.1_ASM394963v1 |  | |
| GCF_004347305.1_ASM434730v1 |  | |
| GCF_006491985.1_ASM649198v1 |  | |
| GCF_006492065.1_ASM649206v1 |  | |
| GCF_006492525.1_ASM649252v1 |  | |
| GCF_006494165.1_ASM649416v1 |  | |
| GCF_006494215.1_ASM649421v1 |  | |
| GCF_900019975.1_acinetobacter_baumannii_isolate_strainE |  | |
| GCF_900029355.1_acinetobacter_baumannii_isolate_strainF |  | |
| GCF_900029375.1_acinetobacter_baumannii_isolate_strainI |  | |
| GCF_900042755.1_acinetobacter_baumannii_isolate_strainG |  | |
| GCF_900043985.1_acinetobacter_baumannii_isolate_strainJ |  | |
| GCF_900043995.1_acinetobacter_baumannii_isolate_strainK |  | |
| GCF_900240205.1_ACB5 |  | |
| GCF_900406715.1_De_novo_assembly_of_KCRI_isolate_RDK04_363 |  | |
| GCF_900406745.1_De_novo_assembly_of_KCRI_isolate_RDK06_423 |  | |
| GCF_900406755.1_De_novo_assembly_of_KCRI_isolate_RDK05_558 |  | |
| GCF_900406765.1_De_novo_assembly_of_KCRI_isolate_RDK05_518B |  | |
| GCF_901484875.1_aba_5m_assembly |  | |
| GCF_901669955.1_aba1_assembly |  | |
| **Contigs Outgroup Group 4** | **Scaffolds Outgroup Group 4** | |
| GCF_000173395.1_ASM17339v1 | GCF_000162295.1_ASM16229v1 |  |
| GCF_000177695.1_ASM17769v1 | GCF_000163355.2_ASM16335v2 |  |
| GCF_000177715.1_ASM17771v1 | GCF_000163375.2_ASM16337v2 |  |
| GCF_000184475.1_ASM18447v2 | GCF_000163395.2_ASM16339v2 |  |
| GCF_000184495.1_ASM18449v2 | GCF_000297915.1_Acin_baum_Ab11111_V1 |  |
| GCF_000184515.1_ASM18451v2 | GCF_000297935.1_Acin_baum_Ab33333_V1 |  |
| GCF_000186645.1_ASM18664v2 | GCF_000297955.1_Acin_baum_Ab44444_V1 |  |
| GCF_000189655.1_ASM18965v2 | GCF_000367885.1_Acin_baum_NIPH_24_V1 |  |
| GCF_000189675.1_ASM18967v2 | GCF_000368105.1_Acin_baum_NIPH_1669_V1 |  |
| GCF_000189695.1_ASM18969v2 | GCF_000368125.1_Acin_baum_NIPH_1362_V1 |  |
| GCF_000214965.1_gacin09v1.0 | GCF_000368185.1_Acin_baum_NIPH_146_V1 |  |
| GCF_000214985.1_gacin10v1.0 | GCF_000368205.1_Acin_baum_NIPH_615_V1 |  |
| GCF_000215005.1_AcbauOIFC032v1.0 | GCF_000368225.1_Acin_baum_NIPH_2061_V1 |  |
| GCF_000222225.1_ASM22222v2 | GCF_000368245.1_Acin_baum_NIPH_1734_V1 |  |
| GCF_000222245.1_ASM22224v2 | GCF_000368525.1_Acin_baum_NIPH_190_V1 |  |
| GCF_000222265.1_ASM22226v2 | GCF_000368545.1_Acin_baum_NIPH_60_V1 |  |
| GCF_000222285.1_ASM22228v2 | GCF_000369165.1_Acin_baum_NIPH_201_V1 |  |
| GCF_000241705.1_ASM24170v2 | GCF_000369205.1_Acin_baum_NIPH_335_V1 |  |
| GCF_000241725.1_ASM24172v2 | GCF_000369225.1_Acin_baum_NIPH_329_V1 |  |
| GCF_000248275.1_ASM24827v2 | GCF_000369245.1_Acin_baum_NIPH_601_V1 |  |
| GCF_000278605.1_gacin08v1.0 | GCF_000369265.1_Acin_baum_NIPH_67_V1 |  |
| GCF_000278625.1_AcbauOIFC137v1.0 | GCF_000369285.1_Acin_baum_NIPH_528_V1 |  |
| GCF_000278645.1_AcbauOIFC109v1.0 | GCF_000369305.1_Acin_baum_NIPH_70_V1 |  |
| GCF_000278665.1_AcbauOIFC143v1.0 | GCF_000369345.1_Acin_baum_NIPH_80_V1 |  |
| GCF_000278685.1_AcbauOIFC189v1.0 | GCF_000369385.1_Acin_baum_CIP_70_34T_V1 |  |
| GCF_000286535.1_gacin11v1.0 | GCF_000413915.1_Acin_baum_NIPH_410_V1 |  |
| GCF_000292545.1_ASM29254v1 | GCF_000417785.2_ASM41778v2 |  |
| GCF_000297515.1_gacin24v1.0 | GCF_000417805.2_ASM41780v2 |  |
| GCF_000297535.1_gacin24v1.0 | GCF_000623015.1_Acin_baum_LAC-4_V1 |  |
| GCF_000297575.1_gacin25v1.0 | GCF_000692095.1_Acin_baum_BIDMC_56_V1 |  |
| GCF_000299655.1_ASM29965v1 | GCF_000737145.1_ASM73714v1 |  |
| GCF_000299675.1_ASM29967v1 | GCF_000761465.1_ASM76146v1 |  |
| GCF_000301175.1_ASM30117v1 | GCF_000787335.1_ASM78733v1 |  |
| GCF_000301195.1_ASM30119v1 | GCF_000802885.1_ASM80288v1 |  |
| GCF_000301215.1_ASM30121v1 | GCF_000802895.1_ASM80289v1 |  |
| GCF_000301235.1_ASM30123v1 | GCF_000802915.1_ASM80291v1 |  |
| GCF_000301255.1_ASM30125v1 | GCF_000804635.1_ASM80463v1 |  |
| GCF_000301275.1_ASM30127v1 | GCF_000804645.1_ASM80464v1 |  |
| GCF_000301295.1_ASM30129v1 | GCF_000804655.1_ASM80465v1 |  |
| GCF_000301315.1_ASM30131v1 | GCF_000804665.1_ASM80466v1 |  |
| GCF_000301335.1_ASM30133v1 | GCF_000804715.1_ASM80471v1 |  |
| GCF_000301355.1_ASM30135v1 | GCF_000804725.1_ASM80472v1 |  |
| GCF_000301375.1_ASM30137v1 | GCF_000804735.1_ASM80473v1 |  |
| GCF_000301395.1_ASM30139v1 | GCF_000804745.1_ASM80474v1 |  |
| GCF_000301415.1_ASM30141v1 | GCF_000804795.1_ASM80479v1 |  |
| GCF_000301455.1_ASM30145v1 | GCF_000804805.1_ASM80480v1 |  |
| GCF_000301475.1_ASM30147v1 | GCF_000804815.1_ASM80481v1 |  |
| GCF_000301495.1_ASM30149v1 | GCF_000804825.1_ASM80482v1 |  |
| GCF_000301515.1_ASM30151v1 | GCF_000804875.1_ASM80487v1 |  |
| GCF_000301535.1_ASM30153v1 | GCF_000804885.1_ASM80488v1 |  |
| GCF_000301555.1_ASM30155v1 | GCF_000804895.1_ASM80489v1 |  |
| GCF_000301575.1_ASM30157v1 | GCF_000804905.1_ASM80490v1 |  |
| GCF_000301595.1_ASM30159v1 | GCF_000804955.1_ASM80495v1 |  |
| GCF_000301615.1_ASM30161v1 | GCF_000804965.1_ASM80496v1 |  |
| GCF_000301655.1_ASM30165v1 | GCF_000804975.1_ASM80497v1 |  |
| GCF_000301835.1_ASM30183v1 | GCF_000804985.1_ASM80498v1 |  |
| GCF_000301855.1_ASM30185v1 | GCF_000805035.1_ASM80503v1 |  |
| GCF_000301875.1_ASM30187v1 | GCF_000805045.1_ASM80504v1 |  |
| GCF_000301895.1_ASM30189v1 | GCF_000805055.1_ASM80505v1 |  |
| GCF_000301915.1_ASM30191v1 | GCF_000805065.1_ASM80506v1 |  |
| GCF_000301935.1_ASM30193v1 | GCF_000805115.1_ASM80511v1 |  |
| GCF_000301955.1_ASM30195v1 | GCF_000805125.1_ASM80512v1 |  |
| GCF_000301975.1_ASM30197v1 | GCF_000805135.1_ASM80513v1 |  |
| GCF_000301995.1_ASM30199v1 | GCF_000805145.1_ASM80514v1 |  |
| GCF_000302015.1_ASM30201v1 | GCF_000805195.1_ASM80519v1 |  |
| GCF_000302055.1_ASM30205v1 | GCF_000805205.1_ASM80520v1 |  |
| GCF_000302075.1_ASM30207v1 | GCF_000805215.1_ASM80521v1 |  |
| GCF_000302095.1_ASM30209v1 | GCF_000805225.1_ASM80522v1 |  |
| GCF_000302135.1_ASM30213v1 | GCF_000805275.1_ASM80527v1 |  |
| GCF_000302155.1_ASM30215v1 | GCF_000805295.1_ASM80529v1 |  |
| GCF_000302175.1_ASM30217v1 | GCF_000805305.1_ASM80530v1 |  |
| GCF_000302195.1_ASM30219v1 | GCF_000876445.1_ASM87644v1 |  |
| GCF_000302215.1_ASM30221v1 | GCF_000966495.1_ASM96649v1 |  |
| GCF_000302235.1_ASM30223v1 | GCF_000966525.1_ASM96652v1 |  |
| GCF_000302255.1_ASM30225v1 | GCF_000981405.1_ASM98140v1 |  |
| GCF_000304675.1_GACIN20v1.0 | GCF_001052775.1_ASM105277v1 |  |
| GCF_000305215.1_gacin13v1.0 | GCF_001055965.1_ASM105596v1 |  |
| GCF_000305235.1_gacin14v1.0 | GCF_001420505.1_ASM142050v1 |  |
| GCF_000305255.1_gacin15v1.0 | GCF_001420515.1_ASM142051v1 |  |
| GCF_000305275.1_gacin22v1.0 | GCF_001432275.1_ASM143227v1 |  |
| GCF_000305295.1_gacin16v1.0 | GCF_001512095.1_7521_8_38 |  |
| GCF_000305315.1_gacin21v1.0 | GCF_001516805.1_ASM151680v1 |  |
| GCF_000307895.1_gacin19v1.0 | GCF_001541425.1_ASM154142v1 |  |
| GCF_000308995.1_ASM30899v1 | GCF_001541455.1_ASM154145v1 |  |
| GCF_000309095.1_gacin42v1.0 | GCF_001541525.1_ASM154152v1 |  |
| GCF_000309115.1_gacin45v1.0 | GCF_001541685.1_ASM154168v1 |  |
| GCF_000309135.1_gacin43v1.0 | GCF_001541695.1_ASM154169v1 |  |
| GCF_000309155.1_gacin48v1.0 | GCF_001541705.1_ASM154170v1 |  |
| GCF_000309175.1_gacin34v1.0 | GCF_001541725.1_ASM154172v1 |  |
| GCF_000309195.1_gacin35v1.0 | GCF_001541765.1_ASM154176v1 |  |
| GCF_000309215.1_gacin37v1.0 | GCF_001541775.1_ASM154177v1 |  |
| GCF_000309235.1_gacin38v1.0 | GCF_001541795.1_ASM154179v1 |  |
| GCF_000309255.1_gacin33v1.0 | GCF_001541875.1_ASM154187v1 |  |
| GCF_000314635.1_gacin31v1.0 | GCF_001611995.1_ASM161199v1 |  |
| GCF_000314655.1_gacin32v1.0 | GCF_001612005.1_ASM161200v1 |  |
| GCF_000332855.1_AB53264 | GCF_001612015.1_ASM161201v1 |  |
| GCF_000333715.1_ASM33371v1 | GCF_001612025.1_ASM161202v1 |  |
| GCF_000333735.1_ASM33373v1 | GCF_001612075.1_ASM161207v1 |  |
| GCF_000335535.1_gacin39v1.0 | GCF_001612095.1_ASM161209v1 |  |
| GCF_000335595.1_gacin50v1.0 | GCF_001612105.1_ASM161210v1 |  |
| GCF_000335615.1_gacin44v1.0 | GCF_001612115.1_ASM161211v1 |  |
| GCF_000335635.1_gacin46v1.0 | GCF_001612155.1_ASM161215v1 |  |
| GCF_000335655.1_gacin47v1.0 | GCF_001612175.1_ASM161217v1 |  |
| GCF_000335675.1_gacin49v1.0 | GCF_001612185.1_ASM161218v1 |  |
| GCF_000338835.1_454 | GCF_001612215.1_ASM161221v1 |  |
| GCF_000341985.1_Whole_genome_assembly | GCF_001612235.1_ASM161223v1 |  |
| GCF_000342065.1_454 | GCF_001612285.1_ASM161228v1 |  |
| GCF_000342085.1_454 | GCF_001612315.1_ASM161231v1 |  |
| GCF_000353795.1_ABNIH22_1 | GCF_001612335.1_ASM161233v1 |  |
| GCF_000353815.1_ABNIH23_1 | GCF_001612355.1_ASM161235v1 |  |
| GCF_000353855.1_ABNIH13_1 | GCF_001612365.1_ASM161236v1 |  |
| GCF_000353895.1_ABNIH26_1 | GCF_001612395.1_ASM161239v1 |  |
| GCF_000353915.1_ABNIH25_1 | GCF_001617825.1_ASM161782v1 |  |
| GCF_000353935.1_ABNIH5_1 | GCF_001617835.1_ASM161783v1 |  |
| GCF_000353975.1_ABNIH7_1 | GCF_001617885.1_ASM161788v1 |  |
| GCF_000354035.1_ABNIH14_1 | GCF_001617915.1_ASM161791v1 |  |
| GCF_000354055.1_ABNIH15_1 | GCF_001617945.1_ASM161794v1 |  |
| GCF_000354075.1_ABNIH16_1 | GCF_001648035.1_ASM164803v1 |  |
| GCF_000354095.1_ABNIH17_1 | GCF_001661665.1_ASM166166v1 |  |
| GCF_000354115.1_ABNIH18_1 | GCF_001672535.1_ASM167253v1 |  |
| GCF_000354135.1_ABNIH20_1 | GCF_001680105.1_ASM168010v1 |  |
| GCF_000354155.1_ABNIH24_1 | GCF_001680115.1_ASM168011v1 |  |
| GCF_000359725.1_AB48055 | GCF_001680185.1_ASM168018v1 |  |
| GCF_000453025.1_ASM45302v1 | GCF_001680195.1_ASM168019v1 |  |
| GCF_000453065.1_ASM45306v1 | GCF_001680215.1_ASM168021v1 |  |
| GCF_000453125.1_ASM45312v1 | GCF_001680225.1_ASM168022v1 |  |
| GCF_000453145.1_ASM45314v1 | GCF_001680265.1_ASM168026v1 |  |
| GCF_000453165.1_ASM45316v1 | GCF_001680285.1_ASM168028v1 |  |
| GCF_000453185.1_ASM45318v1 | GCF_001680295.1_ASM168029v1 |  |
| GCF_000453225.1_ASM45322v1 | GCF_001680305.1_ASM168030v1 |  |
| GCF_000453245.1_ASM45324v1 | GCF_001680345.1_ASM168034v1 |  |
| GCF_000453265.1_ASM45326v1 | GCF_001680365.1_ASM168036v1 |  |
| GCF_000453285.1_ASM45328v1 | GCF_001680375.1_ASM168037v1 |  |
| GCF_000453305.1_ASM45330v1 | GCF_001680395.1_ASM168039v1 |  |
| GCF_000453325.1_ASM45332v1 | GCF_001680425.1_ASM168042v1 |  |
| GCF_000453345.1_ASM45334v1 | GCF_001680445.1_ASM168044v1 |  |
| GCF_000453365.1_ASM45336v1 | GCF_001680475.1_ASM168047v1 |  |
| GCF_000453385.1_ASM45338v1 | GCF_001693095.1_ASM169309v1 |  |
| GCF_000453405.1_ASM45340v1 | GCF_001693105.1_ASM169310v1 |  |
| GCF_000453425.1_ASM45342v1 | GCF_001693115.1_ASM169311v1 |  |
| GCF_000453445.1_ASM45344v1 | GCF_001693175.1_ASM169317v1 |  |
| GCF_000453465.1_ASM45346v1 | GCF_001700395.1_ASM170039v1 |  |
| GCF_000453485.1_ASM45348v1 | GCF_001700445.1_ASM170044v1 |  |
| GCF_000453505.1_ASM45350v1 | GCF_001704675.1_ASM170467v1 |  |
| GCF_000453525.1_ASM45352v1 | GCF_001704685.1_ASM170468v1 |  |
| GCF_000453545.1_ASM45354v1 | GCF_001704705.1_ASM170470v1 |  |
| GCF_000453565.1_ASM45356v1 | GCF_001707985.1_ASM170798v1 |  |
| GCF_000453585.1_ASM45358v1 | GCF_001721365.1_ASM172136v1 |  |
| GCF_000453605.1_ASM45360v1 | GCF_001721375.1_ASM172137v1 |  |
| GCF_000453625.1_ASM45362v1 | GCF_001721385.1_ASM172138v1 |  |
| GCF_000453645.1_ASM45364v1 | GCF_001721425.1_ASM172142v1 |  |
| GCF_000453665.1_ASM45366v1 | GCF_001721445.1_ASM172144v1 |  |
| GCF_000453685.1_ASM45368v1 | GCF_001721455.1_ASM172145v1 |  |
| GCF_000453705.1_ASM45370v1 | GCF_001721465.1_ASM172146v1 |  |
| GCF_000453725.1_ASM45372v1 | GCF_001721475.1_ASM172147v1 |  |
| GCF_000453745.1_ASM45374v1 | GCF_001861375.1_ASM186137v1 |  |
| GCF_000453765.1_ASM45376v1 | GCF_001861385.1_ASM186138v1 |  |
| GCF_000453785.1_ASM45378v1 | GCF_001861395.1_ASM186139v1 |  |
| GCF_000453805.1_ASM45380v1 | GCF_001861435.1_ASM186143v1 |  |
| GCF_000453825.1_ASM45382v1 | GCF_001861455.1_ASM186145v1 |  |
| GCF_000453845.1_ASM45384v1 | GCF_001861465.1_ASM186146v1 |  |
| GCF_000453865.1_ASM45386v1 | GCF_001861475.1_ASM186147v1 |  |
| GCF_000453905.1_ASM45390v1 | GCF_001861515.1_ASM186151v1 |  |
| GCF_000453925.1_ASM45392v1 | GCF_001861535.1_ASM186153v1 |  |
| GCF_000453965.1_ASM45396v1 | GCF_001861545.1_ASM186154v1 |  |
| GCF_000453985.1_ASM45398v1 | GCF_001861565.1_ASM186156v1 |  |
| GCF_000465635.2_GS_De_Novo_Assembly | GCF_001861585.1_ASM186158v1 |  |
| GCF_000493615.1_ABIBUN_1 | GCF_001861615.1_ASM186161v1 |  |
| GCF_000509305.1_ASM50930v1 | GCF_001861655.1_ASM186165v1 |  |
| GCF_000509325.1_ASM50932v1 | GCF_001861665.1_ASM186166v1 |  |
| GCF_000509345.1_ASM50934v1 | GCF_001861695.1_ASM186169v1 |  |
| GCF_000513795.2_gabuh0207_v01 | GCF_001861735.1_ASM186173v1 |  |
| GCF_000515435.1_gabuh0807_v01 | GCF_001861745.1_ASM186174v1 |  |
| GCF_000515455.1_gabuh1007_v01 | GCF_001861775.1_ASM186177v1 |  |
| GCF_000515475.1_gabuh10007_v01 | GCF_001861785.1_ASM186178v1 |  |
| GCF_000515495.1_gabuh0707_v01 | GCF_001861795.1_ASM186179v1 |  |
| GCF_000515515.1_gabuh10107_v01 | GCF_001861835.1_ASM186183v1 |  |
| GCF_000515535.1_gabuh10707_v01 | GCF_001861855.1_ASM186185v1 |  |
| GCF_000515555.1_gabuh11608_v01 | GCF_001861865.1_ASM186186v1 |  |
| GCF_000515575.1_gabuh12208_v01 | GCF_001861895.1_ASM186189v1 |  |
| GCF_000515595.1_gabuh12308_v01 | GCF_001861905.1_ASM186190v1 |  |
| GCF_000515615.1_gabuh12408_v01 | GCF_001861935.1_ASM186193v1 |  |
| GCF_000515635.1_gabuh12808_v01 | GCF_001861955.1_ASM186195v1 |  |
| GCF_000515655.1_gabuh13908_v01 | GCF_001861975.1_ASM186197v1 |  |
| GCF_000515675.1_gabuh14508_v01 | GCF_001861995.1_ASM186199v1 |  |
| GCF_000515695.1_gabuh15208_v01 | GCF_001862005.1_ASM186200v1 |  |
| GCF_000515715.1_gabuh16008_v01 | GCF_001862035.1_ASM186203v1 |  |
| GCF_000515735.1_gabuh16108_v01 | GCF_001862055.1_ASM186205v1 |  |
| GCF_000515755.1_gabuh16208_v01 | GCF_001862065.1_ASM186206v1 |  |
| GCF_000515775.1_gabuh18608_v01 | GCF_001862075.1_ASM186207v1 |  |
| GCF_000515795.1_gabuh19608_v01 | GCF_001862105.1_ASM186210v1 |  |
| GCF_000515815.1_gabuh19908_v01 | GCF_001862135.1_ASM186213v1 |  |
| GCF_000515835.1_gabuh20108_v01 | GCF_001862145.1_ASM186214v1 |  |
| GCF_000515855.1_gabuh22908_v01 | GCF_001862175.1_ASM186217v1 |  |
| GCF_000515875.2_gabuh8907_v01 | GCF_001862185.1_ASM186218v1 |  |
| GCF_000515895.1_gabuh2307_v01 | GCF_001862215.1_ASM186221v1 |  |
| GCF_000515915.1_gabuh8107_v01 | GCF_001862225.1_ASM186222v1 |  |
| GCF_000515935.1_gabuh8407_v01 | GCF_001862235.1_ASM186223v1 |  |
| GCF_000515955.1_gabuh8707_v01 | GCF_001862265.1_ASM186226v1 |  |
| GCF_000515975.1_gabuh8807_v01 | GCF_001862295.1_ASM186229v1 |  |
| GCF_000515995.2_gabuh9007_v01 | GCF_001862305.1_ASM186230v1 |  |
| GCF_000516015.1_gabuh9707_v01 | GCF_001862335.1_ASM186233v1 |  |
| GCF_000516035.1_gabuh9907_v01 | GCF_001862345.1_ASM186234v1 |  |
| GCF_000516055.1_gabuh2907_v01 | GCF_001862375.1_ASM186237v1 |  |
| GCF_000516075.1_gabuh2707_v01 | GCF_001862385.1_ASM186238v1 |  |
| GCF_000516095.1_gabuh99pb_v01 | GCF_001862395.1_ASM186239v1 |  |
| GCF_000516115.1_gabuh107pb_v01 | GCF_001862425.1_ASM186242v1 |  |
| GCF_000516135.1_gabuh5107_v01 | GCF_001862455.1_ASM186245v1 |  |
| GCF_000516155.1_gabuh5207_v01 | GCF_001862475.1_ASM186247v1 |  |
| GCF_000516175.1_gabuh5307_v01 | GCF_001862485.1_ASM186248v1 |  |
| GCF_000516195.2_gabuh5707_v01 | GCF_001862505.1_ASM186250v1 |  |
| GCF_000516215.2_gabuh6107_v01 | GCF_001862535.1_ASM186253v1 |  |
| GCF_000516235.1_gabuh6207_v01 | GCF_001862555.1_ASM186255v1 |  |
| GCF_000516255.1_gabuh6507_v01 | GCF_001862565.1_ASM186256v1 |  |
| GCF_000516275.1_gabuh6907_v01 | GCF_001862585.1_ASM186258v1 |  |
| GCF_000516295.1_gabuh7007_v01 | GCF_001862615.1_ASM186261v1 |  |
| GCF_000516315.1_gabuh7607_v01 | GCF_001862635.1_ASM186263v1 |  |
| GCF_000516335.2_gabuh7707_v01 | GCF_001862645.1_ASM186264v1 |  |
| GCF_000516355.2_gabuh7807_v01 | GCF_001862665.1_ASM186266v1 |  |
| GCF_000516375.1_gabuh7907_v01 | GCF_001862715.1_ASM186271v1 |  |
| GCF_000516575.2_ASM51657v2 | GCF_001862725.1_ASM186272v1 |  |
| GCF_000516595.2_ASM51659v2 | GCF_001862735.1_ASM186273v1 |  |
| GCF_000516635.2_ASM51663v2 | GCF_001862775.1_ASM186277v1 |  |
| GCF_000517485.2_gabuh2107_v01 | GCF_001862795.1_ASM186279v1 |  |
| GCF_000517505.1_gabuh3807_v01 | GCF_001862805.1_ASM186280v1 |  |
| GCF_000580135.1_ASM58013v1 | GCF_001862825.1_ASM186282v1 |  |
| GCF_000580155.1_ASM58015v1 | GCF_001862855.1_ASM186285v1 |  |
| GCF_000580175.1_ASM58017v1 | GCF_001862875.1_ASM186287v1 |  |
| GCF_000580195.1_ASM58019v1 | GCF_001862885.1_ASM186288v1 |  |
| GCF_000580215.1_ASM58021v1 | GCF_001862895.1_ASM186289v1 |  |
| GCF_000580235.1_ASM58023v1 | GCF_001862955.1_ASM186295v1 |  |
| GCF_000580255.1_ASM58025v1 | GCF_001862975.1_ASM186297v1 |  |
| GCF_000580275.1_ASM58027v1 | GCF_001862985.1_ASM186298v1 |  |
| GCF_000580295.1_ASM58029v1 | GCF_001863015.1_ASM186301v1 |  |
| GCF_000580315.1_ASM58031v1 | GCF_001863035.1_ASM186303v1 |  |
| GCF_000580335.1_ASM58033v1 | GCF_001863055.1_ASM186305v1 |  |
| GCF_000580355.1_ASM58035v2 | GCF_001863065.1_ASM186306v1 |  |
| GCF_000580375.1_ASM58037v1 | GCF_001863085.1_ASM186308v1 |  |
| GCF_000580395.1_ASM58039v1 | GCF_001863115.1_ASM186311v1 |  |
| GCF_000580415.1_ASM58041v1 | GCF_001863135.1_ASM186313v1 |  |
| GCF_000580435.1_ASM58043v2 | GCF_001863145.1_ASM186314v1 |  |
| GCF_000580455.1_ASM58045v1 | GCF_001863165.1_ASM186316v1 |  |
| GCF_000580475.1_ASM58047v1 | GCF_001863205.1_ASM186320v1 |  |
| GCF_000580495.1_ASM58049v2 | GCF_001863225.1_ASM186322v1 |  |
| GCF_000580515.1_ASM58051v1 | GCF_001863235.1_ASM186323v1 |  |
| GCF_000580535.1_ASM58053v1 | GCF_001863275.1_ASM186327v1 |  |
| GCF_000580555.1_ASM58055v1 | GCF_001863295.1_ASM186329v1 |  |
| GCF_000580575.1_ASM58057v1 | GCF_001863315.1_ASM186331v1 |  |
| GCF_000580595.1_ASM58059v1 | GCF_001863335.1_ASM186333v1 |  |
| GCF_000580615.1_ASM58061v1 | GCF_001863345.1_ASM186334v1 |  |
| GCF_000580655.1_ASM58065v1 | GCF_001863395.1_ASM186339v1 |  |
| GCF_000580695.1_ASM58069v1 | GCF_001863405.1_ASM186340v1 |  |
| GCF_000580715.1_ASM58071v1 | GCF_001863415.1_ASM186341v1 |  |
| GCF_000580735.1_ASM58073v1 | GCF_001863455.1_ASM186345v1 |  |
| GCF_000580755.1_ASM58075v1 | GCF_001863465.1_ASM186346v1 |  |
| GCF_000580775.1_ASM58077v1 | GCF_001863475.1_ASM186347v1 |  |
| GCF_000580795.1_ASM58079v1 | GCF_001863485.1_ASM186348v1 |  |
| GCF_000580815.1_ASM58081v1 | GCF_001863535.1_ASM186353v1 |  |
| GCF_000580835.1_ASM58083v1 | GCF_001863545.1_ASM186354v1 |  |
| GCF_000580875.1_ASM58087v1 | GCF_001863555.1_ASM186355v1 |  |
| GCF_000580895.1_ASM58089v1 | GCF_001863575.1_ASM186357v1 |  |
| GCF_000580915.1_ASM58091v1 | GCF_001863615.1_ASM186361v1 |  |
| GCF_000580935.1_ASM58093v1 | GCF_001863625.1_ASM186362v1 |  |
| GCF_000580975.1_ASM58097v1 | GCF_001863665.1_ASM186366v1 |  |
| GCF_000580995.1_ASM58099v1 | GCF_001863695.1_ASM186369v1 |  |
| GCF_000581015.1_ASM58101v1 | GCF_001863705.1_ASM186370v1 |  |
| GCF_000581055.1_ASM58105v1 | GCF_001863725.1_ASM186372v1 |  |
| GCF_000581075.1_ASM58107v1 | GCF_001863775.1_ASM186377v1 |  |
| GCF_000581135.1_ASM58113v1 | GCF_001863785.1_ASM186378v1 |  |
| GCF_000581155.1_ASM58115v1 | GCF_001863815.1_ASM186381v1 |  |
| GCF_000581175.1_ASM58117v1 | GCF_001863835.1_ASM186383v1 |  |
| GCF_000581235.1_ASM58123v1 | GCF_001863855.1_ASM186385v1 |  |
| GCF_000581255.1_ASM58125v1 | GCF_001863865.1_ASM186386v1 |  |
| GCF_000581295.1_ASM58129v1 | GCF_001863875.1_ASM186387v1 |  |
| GCF_000581315.1_ASM58131v1 | GCF_001863905.1_ASM186390v1 |  |
| GCF_000581335.1_ASM58133v1 | GCF_001863935.1_ASM186393v1 |  |
| GCF_000581355.1_ASM58135v1 | GCF_001863955.1_ASM186395v1 |  |
| GCF_000581375.1_ASM58137v1 | GCF_001863965.1_ASM186396v1 |  |
| GCF_000581395.1_ASM58139v1 | GCF_001863975.1_ASM186397v1 |  |
| GCF_000581415.1_ASM58141v1 | GCF_001864015.1_ASM186401v1 |  |
| GCF_000581435.1_ASM58143v1 | GCF_001864035.1_ASM186403v1 |  |
| GCF_000581455.1_ASM58145v1 | GCF_001864045.1_ASM186404v1 |  |
| GCF_000581475.1_ASM58147v1 | GCF_001864065.1_ASM186406v1 |  |
| GCF_000581495.1_ASM58149v1 | GCF_001864095.1_ASM186409v1 |  |
| GCF_000581515.1_ASM58151v1 | GCF_001864115.1_ASM186411v1 |  |
| GCF_000581535.1_ASM58153v1 | GCF_001864125.1_ASM186412v1 |  |
| GCF_000581555.1_ASM58155v1 | GCF_001864155.1_ASM186415v1 |  |
| GCF_000581575.1_ASM58157v1 | GCF_001864165.1_ASM186416v1 |  |
| GCF_000581595.1_ASM58159v1 | GCF_001864195.1_ASM186419v1 |  |
| GCF_000581615.1_ASM58161v1 | GCF_001864235.1_ASM186423v1 |  |
| GCF_000581635.1_ASM58163v1 | GCF_001864285.1_ASM186428v1 |  |
| GCF_000581655.1_ASM58165v1 | GCF_001864305.1_ASM186430v1 |  |
| GCF_000581675.1_ASM58167v1 | GCF_001864315.1_ASM186431v1 |  |
| GCF_000581695.1_ASM58169v1 | GCF_001864355.1_ASM186435v1 |  |
| GCF_000581735.1_ASM58173v1 | GCF_001864375.1_ASM186437v1 |  |
| GCF_000581755.1_ASM58175v1 | GCF_001864385.1_ASM186438v1 |  |
| GCF_000581775.1_ASM58177v1 | GCF_001864395.1_ASM186439v1 |  |
| GCF_000581795.1_ASM58179v1 | GCF_001864435.1_ASM186443v1 |  |
| GCF_000581815.1_ASM58181v1 | GCF_001864455.1_ASM186445v1 |  |
| GCF_000581855.1_ASM58185v1 | GCF_001864465.1_ASM186446v1 |  |
| GCF_000581875.1_ASM58187v1 | GCF_001864475.1_ASM186447v1 |  |
| GCF_000581895.1_ASM58189v1 | GCF_001864505.1_ASM186450v1 |  |
| GCF_000581915.1_ASM58191v1 | GCF_001864535.1_ASM186453v1 |  |
| GCF_000581935.1_ASM58193v1 | GCF_001864555.1_ASM186455v1 |  |
| GCF_000581955.1_ASM58195v1 | GCF_001864565.1_ASM186456v1 |  |
| GCF_000581975.1_ASM58197v1 | GCF_001864575.1_ASM186457v1 |  |
| GCF_000581995.1_ASM58199v1 | GCF_001864615.1_ASM186461v1 |  |
| GCF_000582015.1_ASM58201v1 | GCF_001864635.1_ASM186463v1 |  |
| GCF_000582035.1_ASM58203v1 | GCF_001864655.1_ASM186465v1 |  |
| GCF_000582115.1_ASM58211v1 | GCF_001864665.1_ASM186466v1 |  |
| GCF_000582155.1_ASM58215v1 | GCF_001864675.1_ASM186467v1 |  |
| GCF_000582175.1_ASM58217v1 | GCF_001864715.1_ASM186471v1 |  |
| GCF_000582215.1_ASM58221v1 | GCF_001864745.1_ASM186474v1 |  |
| GCF_000582275.1_ASM58227v1 | GCF_001864755.1_ASM186475v1 |  |
| GCF_000582295.1_ASM58229v1 | GCF_001864795.1_ASM186479v1 |  |
| GCF_000582335.1_ASM58233v1 | GCF_001864815.1_ASM186481v1 |  |
| GCF_000582355.1_ASM58235v1 | GCF_001909135.1_ASM190913v1 |  |
| GCF_000582945.1_ASM58294v1 | GCF_001936675.2_ASM193667v2 |  |
| GCF_000582965.1_ASM58296v2 | GCF_001949935.1_ASM194993v1 |  |
| GCF_000583235.1_ASM58323v1 | GCF_001949945.1_ASM194994v1 |  |
| GCF_000583255.1_ASM58325v1 | GCF_001950035.1_ASM195003v1 |  |
| GCF_000583275.1_ASM58327v1 | GCF_001950065.1_ASM195006v1 |  |
| GCF_000583295.1_ASM58329v1 | GCF_001950165.1_ASM195016v1 |  |
| GCF_000583315.1_ASM58331v1 | GCF_001950195.1_ASM195019v1 |  |
| GCF_000583335.1_ASM58333v1 | GCF_001950215.1_ASM195021v1 |  |
| GCF_000583355.1_ASM58335v1 | GCF_001950285.1_ASM195028v1 |  |
| GCF_000583375.1_ASM58337v1 | GCF_001951035.1_A13v1 |  |
| GCF_000583415.1_ASM58341v1 | GCF_001997285.1_ASM199728v1 |  |
| GCF_000583435.1_ASM58343v1 | GCF_002072795.1_ASM207279v1 |  |
| GCF_000583455.1_ASM58345v1 | GCF_002093815.1_ASM209381v1 |  |
| GCF_000583475.1_ASM58347v1 | GCF_002116575.1_ASM211657v1 |  |
| GCF_000583495.1_ASM58349v1 | GCF_002119265.1_ASM211926v1 |  |
| GCF_000583515.1_ASM58351v1 | GCF_002119355.1_ASM211935v1 |  |
| GCF_000583535.1_ASM58353v1 | GCF_002237955.1_ASM223795v1 |  |
| GCF_000583555.1_ASM58355v1 | GCF_002238075.1_ASM223807v1 |  |
| GCF_000583575.1_ASM58357v1 | GCF_002277145.1_ASM227714v1 |  |
| GCF_000583595.1_ASM58359v1 | GCF_002277175.1_ASM227717v1 |  |
| GCF_000584035.1_ASM58403v1 | GCF_002277185.1_ASM227718v1 |  |
| GCF_000584055.1_ASM58405v1 | GCF_002277215.1_ASM227721v1 |  |
| GCF_000584075.1_ASM58407v1 | GCF_002277225.1_ASM227722v1 |  |
| GCF_000584095.1_ASM58409v1 | GCF_002277235.1_ASM227723v1 |  |
| GCF_000584115.1_ASM58411v1 | GCF_002277275.1_ASM227727v1 |  |
| GCF_000584135.1_ASM58413v1 | GCF_002277285.1_ASM227728v1 |  |
| GCF_000584155.1_ASM58415v1 | GCF_002277315.1_ASM227731v1 |  |
| GCF_000584175.1_ASM58417v1 | GCF_002277325.1_ASM227732v1 |  |
| GCF_000584195.1_ASM58419v1 | GCF_002277355.1_ASM227735v1 |  |
| GCF_000584235.1_ASM58423v1 | GCF_002277365.1_ASM227736v1 |  |
| GCF_000584275.1_ASM58427v1 | GCF_002277385.1_ASM227738v1 |  |
| GCF_000584295.1_ASM58429v1 | GCF_002277405.1_ASM227740v1 |  |
| GCF_000584315.1_ASM58431v1 | GCF_002277435.1_ASM227743v1 |  |
| GCF_000584335.1_ASM58433v1 | GCF_002277445.1_ASM227744v1 |  |
| GCF_000584355.1_ASM58435v1 | GCF_002277455.1_ASM227745v1 |  |
| GCF_000584395.1_ASM58439v1 | GCF_002277485.1_ASM227748v1 |  |
| GCF_000584415.1_ASM58441v1 | GCF_002277505.1_ASM227750v1 |  |
| GCF_000584435.1_ASM58443v1 | GCF_002277515.1_ASM227751v1 |  |
| GCF_000584455.1_ASM58445v1 | GCF_002277535.1_ASM227753v1 |  |
| GCF_000584475.1_ASM58447v1 | GCF_002277575.1_ASM227757v1 |  |
| GCF_000584495.1_ASM58449v1 | GCF_002277585.1_ASM227758v1 |  |
| GCF_000584515.1_ASM58451v2 | GCF_002277605.1_ASM227760v1 |  |
| GCF_000584535.1_ASM58453v1 | GCF_002277635.1_ASM227763v1 |  |
| GCF_000584555.1_ASM58455v1 | GCF_002277655.1_ASM227765v1 |  |
| GCF_000584595.1_ASM58459v1 | GCF_002277675.1_ASM227767v1 |  |
| GCF_000584635.1_ASM58463v1 | GCF_002277705.1_ASM227770v1 |  |
| GCF_000584655.1_ASM58465v1 | GCF_002277715.1_ASM227771v1 |  |
| GCF_000584675.1_ASM58467v1 | GCF_002573795.1_ASM257379v1 |  |
| GCF_000584695.1_ASM58469v1 | GCF_002573815.1_ASM257381v1 |  |
| GCF_000584715.1_ASM58471v1 | GCF_002573905.1_ASM257390v1 |  |
| GCF_000584735.1_ASM58473v1 | GCF_002634325.1_ASM263432v1 |  |
| GCF_000584755.1_ASM58475v1 | GCF_002634345.1_ASM263434v1 |  |
| GCF_000584775.1_ASM58477v1 | GCF_002727055.2_ASM272705v2 |  |
| GCF_000584835.1_ASM58483v1 | GCF_002760695.1_ASM276069v1 |  |
| GCF_000584855.1_ASM58485v1 | GCF_002760705.1_ASM276070v1 |  |
| GCF_000584875.1_ASM58487v1 | GCF_002760715.1_ASM276071v1 |  |
| GCF_000584895.1_ASM58489v1 | GCF_002760725.1_ASM276072v1 |  |
| GCF_000587875.1_ASM58787v1 | GCF_002760765.1_ASM276076v1 |  |
| GCF_000587895.1_ASM58789v1 | GCF_002760785.1_ASM276078v1 |  |
| GCF_000587915.1_ASM58791v1 | GCF_002760825.1_ASM276082v1 |  |
| GCF_000587935.1_ASM58793v1 | GCF_002760835.1_ASM276083v1 |  |
| GCF_000587955.1_ASM58795v1 | GCF_002760845.1_ASM276084v1 |  |
| GCF_000587975.1_ASM58797v1 | GCF_002803655.2_ASM280365v2 |  |
| GCF_000588035.1_ASM58803v1 | GCF_002811175.1_ASM281117v1 |  |
| GCF_000588055.1_ASM58805v1 | GCF_002837885.2_ASM283788v3 |  |
| GCF_000588075.1_ASM58807v1 | GCF_002837895.2_ASM283789v2 |  |
| GCF_000588115.1_ASM58811v1 | GCF_002837905.2_ASM283790v2 |  |
| GCF_000588135.1_ASM58813v1 | GCF_002837945.2_ASM283794v2 |  |
| GCF_000588155.1_ASM58815v1 | GCF_002837985.2_ASM283798v2 |  |
| GCF_000588175.1_ASM58817v1 | GCF_002838015.2_ASM283801v2 |  |
| GCF_000588195.1_ASM58819v1 | GCF_002838025.2_ASM283802v2 |  |
| GCF_000588215.1_ASM58821v1 | GCF_002863545.1_ASM286354v1 |  |
| GCF_000588235.1_ASM58823v1 | GCF_002927775.1_ASM292777v1 |  |
| GCF_000588275.1_ASM58827v1 | GCF_002927805.1_ASM292780v1 |  |
| GCF_000588295.1_ASM58829v1 | GCF_002927855.1_ASM292785v1 |  |
| GCF_000588315.1_ASM58831v1 | GCF_002927965.1_ASM292796v1 |  |
| GCF_000588355.1_ASM58835v1 | GCF_002927995.1_ASM292799v1 |  |
| GCF_000588375.1_ASM58837v1 | GCF_002928015.1_ASM292801v1 |  |
| GCF_000588435.1_ASM58843v1 | GCF_002928035.1_ASM292803v1 |  |
| GCF_000588455.1_ASM58845v1 | GCF_002928045.1_ASM292804v1 |  |
| GCF_000588475.1_ASM58847v1 | GCF_002928135.1_ASM292813v1 |  |
| GCF_000588495.1_ASM58849v1 | GCF_002928145.1_ASM292814v1 |  |
| GCF_000588555.1_ASM58855v1 | GCF_002928155.1_ASM292815v1 |  |
| GCF_000588575.1_ASM58857v2 | GCF_002928195.1_ASM292819v1 |  |
| GCF_000588615.1_ASM58861v1 | GCF_002950515.1_ASM295051v1 |  |
| GCF_000588635.1_ASM58863v1 | GCF_002950525.1_ASM295052v1 |  |
| GCF_000588655.1_ASM58865v1 | GCF_002950545.1_ASM295054v1 |  |
| GCF_000588675.1_ASM58867v1 | GCF_002950615.1_ASM295061v1 |  |
| GCF_000588695.1_ASM58869v1 | GCF_002950655.1_ASM295065v1 |  |
| GCF_000588735.1_ASM58873v1 | GCF_002950675.1_ASM295067v1 |  |
| GCF_000588755.1_ASM58875v1 | GCF_002950755.1_ASM295075v1 |  |
| GCF_000588775.1_ASM58877v1 | GCF_002950795.1_ASM295079v1 |  |
| GCF_000588795.1_ASM58879v1 | GCF_002950915.1_ASM295091v1 |  |
| GCF_000588815.1_ASM58881v1 | GCF_002950975.1_ASM295097v1 |  |
| GCF_000588895.1_ASM58889v1 | GCF_002950995.1_ASM295099v1 |  |
| GCF_000588915.1_ASM58891v1 | GCF_002951015.1_ASM295101v1 |  |
| GCF_000588935.1_ASM58893v1 | GCF_002951135.1_ASM295113v1 |  |
| GCF_000588955.1_ASM58895v1 | GCF_002951175.1_ASM295117v1 |  |
| GCF_000588975.1_ASM58897v1 | GCF_002951235.1_ASM295123v1 |  |
| GCF_000588995.1_ASM58899v1 | GCF_002951315.1_ASM295131v1 |  |
| GCF_000589015.1_ASM58901v1 | GCF_002951335.1_ASM295133v1 |  |
| GCF_000589035.1_ASM58903v1 | GCF_002951415.1_ASM295141v1 |  |
| GCF_000589055.1_ASM58905v1 | GCF_003006835.1_ASM300683v1 |  |
| GCF_000589075.1_ASM58907v1 | GCF_003006845.1_ASM300684v1 |  |
| GCF_000589095.1_ASM58909v1 | GCF_003006875.1_ASM300687v1 |  |
| GCF_000589195.1_ASM58919v1 | GCF_003006945.1_ASM300694v1 |  |
| GCF_000589275.1_ASM58927v1 | GCF_003006975.1_ASM300697v1 |  |
| GCF_000589295.1_ASM58929v1 | GCF_003006995.1_ASM300699v1 |  |
| GCF_000589315.1_ASM58931v1 | GCF_003007015.1_ASM300701v1 |  |
| GCF_000589355.1_ASM58935v1 | GCF_003007025.1_ASM300702v1 |  |
| GCF_000589375.1_ASM58937v1 | GCF_003007055.1_ASM300705v1 |  |
| GCF_000589395.1_ASM58939v1 | GCF_003007065.1_ASM300706v1 |  |
| GCF_000589415.1_ASM58941v1 | GCF_003007095.1_ASM300709v1 |  |
| GCF_000589435.1_ASM58943v1 | GCF_003007115.1_ASM300711v1 |  |
| GCF_000589455.1_ASM58945v1 | GCF_003007135.1_ASM300713v1 |  |
| GCF_000589475.1_ASM58947v1 | GCF_003007145.1_ASM300714v1 |  |
| GCF_000589495.1_ASM58949v1 | GCF_003007215.1_ASM300721v1 |  |
| GCF_000589515.1_ASM58951v1 | GCF_003007245.1_ASM300724v1 |  |
| GCF_000589535.1_ASM58953v1 | GCF_003007275.1_ASM300727v1 |  |
| GCF_000589555.1_ASM58955v1 | GCF_003007315.1_ASM300731v1 |  |
| GCF_000589575.1_ASM58957v1 | GCF_003007335.1_ASM300733v1 |  |
| GCF_000589595.1_ASM58959v1 | GCF_003007355.1_ASM300735v1 |  |
| GCF_000589615.1_ASM58961v1 | GCF_003007375.1_ASM300737v1 |  |
| GCF_000589635.1_ASM58963v1 | GCF_003007395.1_ASM300739v1 |  |
| GCF_000589655.1_ASM58965v1 | GCF_003007405.1_ASM300740v1 |  |
| GCF_000589675.1_ASM58967v1 | GCF_003007435.1_ASM300743v1 |  |
| GCF_000589695.1_ASM58969v1 | GCF_003007455.1_ASM300745v1 |  |
| GCF_000589715.1_ASM58971v1 | GCF_003007475.1_ASM300747v1 |  |
| GCF_000589735.1_ASM58973v1 | GCF_003007485.1_ASM300748v1 |  |
| GCF_000589755.1_ASM58975v1 | GCF_003007515.1_ASM300751v1 |  |
| GCF_000589775.1_ASM58977v1 | GCF_003007535.1_ASM300753v1 |  |
| GCF_000589795.1_ASM58979v1 | GCF_003007585.1_ASM300758v1 |  |
| GCF_000589815.1_ASM58981v1 | GCF_003007615.1_ASM300761v1 |  |
| GCF_000589835.1_ASM58983v1 | GCF_003007635.1_ASM300763v1 |  |
| GCF_000591575.1_ASM59157v1 | GCF_003007655.1_ASM300765v1 |  |
| GCF_000591595.1_ASM59159v1 | GCF_003052285.1_ASM305228v1 |  |
| GCF_000591615.1_ASM59161v1 | GCF_003056465.1_ASM305646v1 |  |
| GCF_000591635.1_ASM59163v1 | GCF_003056475.1_ASM305647v1 |  |
| GCF_000591655.1_ASM59165v1 | GCF_003056505.1_ASM305650v1 |  |
| GCF_000591675.1_ASM59167v1 | GCF_003056515.1_ASM305651v1 |  |
| GCF_000591695.1_ASM59169v1 | GCF_003056525.1_ASM305652v1 |  |
| GCF_000591715.1_ASM59171v1 | GCF_003070905.1_ASM307090v1 |  |
| GCF_000591735.1_ASM59173v1 | GCF_003070915.1_ASM307091v1 |  |
| GCF_000591755.1_ASM59175v1 | GCF_003070935.1_ASM307093v1 |  |
| GCF_000591775.1_ASM59177v1 | GCF_003070945.1_ASM307094v1 |  |
| GCF_000591795.1_ASM59179v1 | GCF_003072095.1_ASM307209v1 |  |
| GCF_000591815.1_ASM59181v1 | GCF_003130905.1_ASM313090v1 |  |
| GCF_000591835.1_ASM59183v1 | GCF_003130915.1_ASM313091v1 |  |
| GCF_000591855.1_ASM59185v1 | GCF_003130925.1_ASM313092v1 |  |
| GCF_000591875.1_ASM59187v1 | GCF_003130945.1_ASM313094v1 |  |
| GCF_000591895.1_ASM59189v1 | GCF_003130985.1_ASM313098v1 |  |
| GCF_000591915.1_ASM59191v1 | GCF_003131005.1_ASM313100v1 |  |
| GCF_000591935.1_ASM59193v1 | GCF_003131025.1_ASM313102v1 |  |
| GCF_000591955.1_ASM59195v1 | GCF_003131095.1_ASM313109v1 |  |
| GCF_000591975.1_ASM59197v1 | GCF_003183965.1_ASM318396v1 |  |
| GCF_000591995.1_ASM59199v1 | GCF_003184015.1_ASM318401v1 |  |
| GCF_000592015.1_ASM59201v1 | GCF_003231135.1_ASM323113v1 |  |
| GCF_000592035.1_ASM59203v1 | GCF_003231175.1_ASM323117v1 |  |
| GCF_000592055.1_ASM59205v1 | GCF_003231185.1_ASM323118v1 |  |
| GCF_000592075.1_ASM59207v1 | GCF_003240485.1_ASM324048v1 |  |
| GCF_000592095.1_ASM59209v1 | GCF_003240535.1_ASM324053v1 |  |
| GCF_000592115.1_ASM59211v1 | GCF_003240555.1_ASM324055v1 |  |
| GCF_000592135.1_ASM59213v1 | GCF_003332235.1_ASM333223v1 |  |
| GCF_000592155.1_ASM59215v1 | GCF_003335925.1_ASM333592v1 |  |
| GCF_000592175.1_ASM59217v1 | GCF_003335945.1_ASM333594v1 |  |
| GCF_000592195.1_ASM59219v1 | GCF_003335955.1_ASM333595v1 |  |
| GCF_000592215.1_ASM59221v1 | GCF_003335965.1_ASM333596v1 |  |
| GCF_000592235.1_ASM59223v1 | GCF_003336005.1_ASM333600v1 |  |
| GCF_000592255.1_ASM59225v1 | GCF_003336035.1_ASM333603v1 |  |
| GCF_000592275.1_ASM59227v1 | GCF_003336045.1_ASM333604v1 |  |
| GCF_000592295.1_ASM59229v1 | GCF_003336075.1_ASM333607v1 |  |
| GCF_000592315.1_ASM59231v1 | GCF_003336085.1_ASM333608v1 |  |
| GCF_000592335.1_ASM59233v1 | GCF_003336135.1_ASM333613v1 |  |
| GCF_000592355.1_ASM59235v1 | GCF_003336155.1_ASM333615v1 |  |
| GCF_000592375.1_ASM59237v1 | GCF_003352485.1_ASM335248v1 |  |
| GCF_000592395.1_ASM59239v1 | GCF_003352495.1_ASM335249v1 |  |
| GCF_000592415.1_ASM59241v1 | GCF_003352525.1_ASM335252v1 |  |
| GCF_000592435.1_ASM59243v1 | GCF_003352535.1_ASM335253v1 |  |
| GCF_000592455.1_ASM59245v1 | GCF_003352595.1_ASM335259v1 |  |
| GCF_000592475.1_ASM59247v1 | GCF_003352605.1_ASM335260v1 |  |
| GCF_000592495.1_ASM59249v1 | GCF_003352615.1_ASM335261v1 |  |
| GCF_000592515.1_ASM59251v1 | GCF_003352665.1_ASM335266v1 |  |
| GCF_000592535.1_ASM59253v1 | GCF_003352675.1_ASM335267v1 |  |
| GCF_000592555.1_ASM59255v1 | GCF_003352685.1_ASM335268v1 |  |
| GCF_000592575.1_ASM59257v1 | GCF_003352725.1_ASM335272v1 |  |
| GCF_000592595.1_ASM59259v1 | GCF_003352735.1_ASM335273v1 |  |
| GCF_000592615.1_ASM59261v1 | GCF_003355585.1_ASM335558v1 |  |
| GCF_000592635.1_ASM59263v1 | GCF_003355595.1_ASM335559v1 |  |
| GCF_000592655.1_ASM59265v1 | GCF_003355605.1_ASM335560v1 |  |
| GCF_000592675.1_ASM59267v1 | GCF_003355665.1_ASM335566v1 |  |
| GCF_000592695.1_ASM59269v1 | GCF_003355675.1_ASM335567v1 |  |
| GCF_000592715.1_ASM59271v1 | GCF_003355705.1_ASM335570v1 |  |
| GCF_000592735.1_ASM59273v1 | GCF_003355725.1_ASM335572v1 |  |
| GCF_000592755.1_ASM59275v1 | GCF_003355745.1_ASM335574v1 |  |
| GCF_000600375.1_ASM60037v1 | GCF_003355755.1_ASM335575v1 |  |
| GCF_000600395.1_ASM60039v1 | GCF_003355785.1_ASM335578v1 |  |
| GCF_000600415.1_ASM60041v1 | GCF_003355795.1_ASM335579v1 |  |
| GCF_000600435.1_ASM60043v1 | GCF_003355815.1_ASM335581v1 |  |
| GCF_000600455.1_ASM60045v1 | GCF_003355845.1_ASM335584v1 |  |
| GCF_000600475.1_ASM60047v1 | GCF_003355865.1_ASM335586v1 |  |
| GCF_000600495.1_ASM60049v1 | GCF_003355875.1_ASM335587v1 |  |
| GCF_000600515.1_ASM60051v1 | GCF_003355885.1_ASM335588v1 |  |
| GCF_000600535.1_ASM60053v1 | GCF_003355915.1_ASM335591v1 |  |
| GCF_000600555.1_ASM60055v1 | GCF_003355945.1_ASM335594v1 |  |
| GCF_000600575.1_ASM60057v1 | GCF_003355965.1_ASM335596v1 |  |
| GCF_000600595.1_ASM60059v1 | GCF_003355985.1_ASM335598v1 |  |
| GCF_000600615.1_ASM60061v1 | GCF_003355995.1_ASM335599v1 |  |
| GCF_000600635.1_ASM60063v1 | GCF_003356025.1_ASM335602v1 |  |
| GCF_000600655.1_ASM60065v1 | GCF_003356045.1_ASM335604v1 |  |
| GCF_000600675.1_ASM60067v1 | GCF_003356055.1_ASM335605v1 |  |
| GCF_000600695.1_ASM60069v1 | GCF_003356085.1_ASM335608v1 |  |
| GCF_000600715.1_ASM60071v1 | GCF_003356105.1_ASM335610v1 |  |
| GCF_000600735.1_ASM60073v1 | GCF_003356125.1_ASM335612v1 |  |
| GCF_000600755.1_ASM60075v1 | GCF_003356145.1_ASM335614v1 |  |
| GCF_000600775.1_ASM60077v1 | GCF_003356155.1_ASM335615v1 |  |
| GCF_000600795.1_ASM60079v1 | GCF_003356185.1_ASM335618v1 |  |
| GCF_000600815.1_ASM60081v1 | GCF_003356205.1_ASM335620v1 |  |
| GCF_000600835.1_ASM60083v1 | GCF_003356225.1_ASM335622v1 |  |
| GCF_000600855.1_ASM60085v1 | GCF_003356235.1_ASM335623v1 |  |
| GCF_000600875.1_ASM60087v1 | GCF_003356265.1_ASM335626v1 |  |
| GCF_000600895.1_ASM60089v1 | GCF_003356285.1_ASM335628v1 |  |
| GCF_000600915.1_ASM60091v1 | GCF_003356295.1_ASM335629v1 |  |
| GCF_000600935.1_ASM60093v1 | GCF_003356315.1_ASM335631v1 |  |
| GCF_000611045.1_ASM61104v1 | GCF_003356345.1_ASM335634v1 |  |
| GCF_000611065.1_ASM61106v1 | GCF_003356365.1_ASM335636v1 |  |
| GCF_000611085.1_ASM61108v1 | GCF_003356385.1_ASM335638v1 |  |
| GCF_000611105.1_ASM61110v1 | GCF_003356405.1_ASM335640v1 |  |
| GCF_000611165.1_ASM61116v1 | GCF_003356425.1_ASM335642v1 |  |
| GCF_000611185.1_ASM61118v1 | GCF_003356445.1_ASM335644v1 |  |
| GCF_000611205.1_ASM61120v1 | GCF_003356455.1_ASM335645v1 |  |
| GCF_000611225.1_ASM61122v1 | GCF_003356485.1_ASM335648v1 |  |
| GCF_000611245.1_ASM61124v1 | GCF_003356505.1_ASM335650v1 |  |
| GCF_000611265.1_ASM61126v1 |  |  |
| GCF_000611285.1_ASM61128v1 |  |  |
| GCF_000611305.1_ASM61130v1 |  |  |
| GCF_000611325.1_ASM61132v1 |  |  |
| GCF_000611345.1_ASM61134v1 |  |  |
| GCF_000611365.1_ASM61136v1 |  |  |
| GCF_000611385.1_ASM61138v1 |  |  |
| GCF_000611405.1_ASM61140v1 |  |  |
| GCF_000611425.1_ASM61142v1 |  |  |
| GCF_000611445.1_ASM61144v1 |  |  |
| GCF_000611465.1_ASM61146v1 |  |  |
| GCF_000611485.1_ASM61148v1 |  |  |
| GCF_000611505.1_ASM61150v1 |  |  |
| GCF_000611525.1_ASM61152v1 |  |  |
| GCF_000611545.1_ASM61154v1 |  |  |
| GCF_000611565.1_ASM61156v1 |  |  |
| GCF_000627335.1_ASM62733v1 |  |  |
| GCF_000627355.1_ASM62735v1 |  |  |
| GCF_000627375.1_ASM62737v1 |  |  |
| GCF_000627395.1_ASM62739v1 |  |  |
| GCF_000627415.1_ASM62741v1 |  |  |
| GCF_000627495.1_ASM62749v1 |  |  |
| GCF_000627515.1_ASM62751v1 |  |  |
| GCF_000627535.1_ASM62753v1 |  |  |
| GCF_000627555.1_ASM62755v1 |  |  |
| GCF_000627575.1_ASM62757v1 |  |  |
| GCF_000627595.1_ASM62759v1 |  |  |
| GCF_000627615.1_ASM62761v1 |  |  |
| GCF_000627635.1_ASM62763v1 |  |  |
| GCF_000627655.1_ASM62765v1 |  |  |
| GCF_000627675.1_ASM62767v1 |  |  |
| GCF_000627695.1_ASM62769v1 |  |  |
| GCF_000627715.1_ASM62771v1 |  |  |
| GCF_000627735.1_ASM62773v1 |  |  |
| GCF_000627755.1_ASM62775v1 |  |  |
| GCF_000627775.1_ASM62777v1 |  |  |
| GCF_000671415.1_ASM67141v1 |  |  |
| GCF_000671435.1_ASM67143v1 |  |  |
| GCF_000671475.1_ASM67147v1 |  |  |
| GCF_000671495.1_ASM67149v1 |  |  |
| GCF_000671515.1_ASM67151v1 |  |  |
| GCF_000671535.1_ASM67153v1 |  |  |
| GCF_000671555.1_ASM67155v1 |  |  |
| GCF_000671575.1_ASM67157v1 |  |  |
| GCF_000671595.1_ASM67159v1 |  |  |
| GCF_000671615.1_ASM67161v1 |  |  |
| GCF_000671635.1_ASM67163v1 |  |  |
| GCF_000671655.1_ASM67165v1 |  |  |
| GCF_000671675.1_ASM67167v1 |  |  |
| GCF_000681555.1_ASM68155v1 |  |  |
| GCF_000681575.1_ASM68157v1 |  |  |
| GCF_000681595.1_ASM68159v1 |  |  |
| GCF_000681615.1_ASM68161v1 |  |  |
| GCF_000681635.1_ASM68163v1 |  |  |
| GCF_000681655.1_ASM68165v1 |  |  |
| GCF_000681675.1_ASM68167v1 |  |  |
| GCF_000681695.1_ASM68169v1 |  |  |
| GCF_000681735.1_ASM68173v1 |  |  |
| GCF_000681755.1_ASM68175v1 |  |  |
| GCF_000681775.1_ASM68177v1 |  |  |
| GCF_000681795.1_ASM68179v1 |  |  |
| GCF_000681835.1_ASM68183v1 |  |  |
| GCF_000681855.1_ASM68185v1 |  |  |
| GCF_000681875.1_ASM68187v1 |  |  |
| GCF_000681895.1_ASM68189v1 |  |  |
| GCF_000681915.1_ASM68191v1 |  |  |
| GCF_000681935.1_ASM68193v1 |  |  |
| GCF_000681955.1_ASM68195v1 |  |  |
| GCF_000681975.1_ASM68197v1 |  |  |
| GCF_000681995.1_ASM68199v1 |  |  |
| GCF_000682015.1_ASM68201v1 |  |  |
| GCF_000682035.1_ASM68203v1 |  |  |
| GCF_000682055.1_ASM68205v1 |  |  |
| GCF_000682075.1_ASM68207v1 |  |  |
| GCF_000682095.1_ASM68209v1 |  |  |
| GCF_000682115.1_ASM68211v1 |  |  |
| GCF_000682135.1_ASM68213v1 |  |  |
| GCF_000682175.1_ASM68217v1 |  |  |
| GCF_000682215.1_ASM68221v1 |  |  |
| GCF_000682235.1_ASM68223v1 |  |  |
| GCF_000682255.1_ASM68225v1 |  |  |
| GCF_000682275.1_ASM68227v1 |  |  |
| GCF_000682295.1_ASM68229v1 |  |  |
| GCF_000682335.1_ASM68233v1 |  |  |
| GCF_000682375.1_ASM68237v1 |  |  |
| GCF_000682395.1_ASM68239v1 |  |  |
| GCF_000682415.1_ASM68241v1 |  |  |
| GCF_000682435.1_ASM68243v1 |  |  |
| GCF_000682455.1_ASM68245v1 |  |  |
| GCF_000682475.1_ASM68247v1 |  |  |
| GCF_000682495.1_ASM68249v1 |  |  |
| GCF_000682515.1_ASM68251v1 |  |  |
| GCF_000682535.1_ASM68253v1 |  |  |
| GCF_000682555.1_ASM68255v1 |  |  |
| GCF_000682575.1_ASM68257v1 |  |  |
| GCF_000682595.1_ASM68259v1 |  |  |
| GCF_000682655.1_ASM68265v1 |  |  |
| GCF_000708775.1_ASM70877v1 |  |  |
| GCF_000708795.1_ABU310 |  |  |
| GCF_000722965.1_Acinetobacter_baumannii_Genome_sequencing |  |  |
| GCF_000722975.1_Acinetobacter_baumannii_Genome_sequencing |  |  |
| GCF_000722985.1_Acinetobacter_baumannii_Genome_sequencing |  |  |
| GCF_000723045.1_Acinetobacter_baumannii_Genome_sequencing |  |  |
| GCF_000723055.1_Acinetobacter_baumannii_Genome_sequencing |  |  |
| GCF_000723065.1_Acinetobacter_baumannii_Genome_sequencing |  |  |
| GCF_000723075.1_Acinetobacter_baumannii_Genome_sequencing |  |  |
| GCF_000723125.1_Acinetobacter_baumannii_Genome_sequencing |  |  |
| GCF_000723145.1_Acinetobacter_baumannii_Genome_sequencing |  |  |
| GCF_000731965.1_ASM73196v1 |  |  |
| GCF_000731975.1_ASM73197v1 |  |  |
| GCF_000734775.1_ASM73477v1 |  |  |
| GCF_000738845.1_ASM73884v1 |  |  |
| GCF_000750855.1_600_CLC |  |  |
| GCF_000757665.1_ASM75766v1 |  |  |
| GCF_000759395.1_ASM75939v1 |  |  |
| GCF_000760395.1_ASM76039v1 |  |  |
| GCF_000760415.1_ASM76041v1 |  |  |
| GCF_000760435.1_ASM76043v1 |  |  |
| GCF_000760455.1_ASM76045v1 |  |  |
| GCF_000760475.1_ASM76047v1 |  |  |
| GCF_000763365.1_ASM76336v1 |  |  |
| GCF_000766105.1_ASM76610v1 |  |  |
| GCF_000770785.1_ASM77078v1 |  |  |
| GCF_000770805.1_ASM77080v1 |  |  |
| GCF_000770815.1_ASM77081v1 |  |  |
| GCF_000770865.1_ASM77086v1 |  |  |
| GCF_000770885.1_ASM77088v1 |  |  |
| GCF_000770905.1_ASM77090v1 |  |  |
| GCF_000775835.1_ASM77583v1 |  |  |
| GCF_000775855.1_ASM77585v1 |  |  |
| GCF_000775875.1_ASM77587v1 |  |  |
| GCF_000787015.1_ASM78701v1 |  |  |
| GCF_000787215.1_ASM78721v1 |  |  |
| GCF_000787225.1_ASM78722v1 |  |  |
| GCF_000787235.1_ASM78723v1 |  |  |
| GCF_000787275.1_ASM78727v1 |  |  |
| GCF_000787295.1_ASM78729v1 |  |  |
| GCF_000787315.1_ASM78731v1 |  |  |
| GCF_000787355.1_ASM78735v1 |  |  |
| GCF_000805365.1_ASM80536v1 |  |  |
| GCF_000805515.1_ASM80551v1 |  |  |
| GCF_000805525.1_ASM80552v1 |  |  |
| GCF_000805535.1_ASM80553v1 |  |  |
| GCF_000805545.1_ASM80554v1 |  |  |
| GCF_000805595.1_ASM80559v1 |  |  |
| GCF_000805615.1_ASM80561v1 |  |  |
| GCF_000805635.1_ASM80563v1 |  |  |
| GCF_000808765.2_Velvet_assembly_of_Acinetobacter_baumannii_ABUH42783 | |  |
| GCF_000808775.3_Velvet_assembly_of_Acinetobacter_baumannii_ABUH504227 | |  |
| GCF_000808785.3_Velvet_assembly_of_Acinetobacter_baumannii_ABUH4837 | |  |
| GCF_000808815.3_Velvet_assembly_of_Acinetobacter_baumannii_ABUH60486 | |  |
| GCF_000808845.3_Velvet_assembly_of_Acinetobacter_baumannii_ABUH319118 | |  |
| GCF_000808885.3_Velvet_assembly_of_Acinetobacter_baumannii_ABUH497144 | |  |
| GCF_000808905.3_Velvet_assembly_of_Acinetobacter_baumannii_ABUH504238 | |  |
| GCF_000808925.3_Velvet_assembly_of_Acinetobacter_baumannii_ABUH80286 | |  |
| GCF_000808945.3_Velvet_assembly_of_Acinetobacter_baumannii_ABUH518307 | |  |
| GCF_000808985.3_Velvet_assembly_of_Acinetobacter_baumannii_ABUH463346 | |  |
| GCF_000809005.3_Velvet_assembly_of_Acinetobacter_baumannii_ABUH304350 | |  |
| GCF_000809045.3_Velvet_assembly_of_Acinetobacter_baumannii_ABUH304352 | |  |
| GCF_000809065.3_Velvet_assembly_of_Acinetobacter_baumannii_ABUH524354 | |  |
| GCF_000809085.3_Velvet_assembly_of_Acinetobacter_baumannii_ABUH404571 | |  |
| GCF_000809205.3_ASM80920v3 |  |  |
| GCF_000809215.3_ASM80921v3 |  |  |
| GCF_000809225.3_ASM80922v3 |  |  |
| GCF_000809235.3_ASM80923v3 |  |  |
| GCF_000809285.3_ASM80928v3 |  |  |
| GCF_000809305.3_ASM80930v3 |  |  |
| GCF_000809325.3_ASM80932v3 |  |  |
| GCF_000809345.3_ASM80934v3 |  |  |
| GCF_000809365.3_ASM80936v3 |  |  |
| GCF_000809385.3_ASM80938v3 |  |  |
| GCF_000809405.3_ASM80940v3 |  |  |
| GCF_000809425.3_ASM80942v3 |  |  |
| GCF_000809445.3_ASM80944v3 |  |  |
| GCF_000809465.3_ASM80946v3 |  |  |
| GCF_000809485.3_ASM80948v3 |  |  |
| GCF_000809505.3_ASM80950v3 |  |  |
| GCF_000809525.3_ASM80952v3 |  |  |
| GCF_000809545.3_ASM80954v3 |  |  |
| GCF_000809565.3_ASM80956v3 |  |  |
| GCF_000809585.3_ASM80958v3 |  |  |
| GCF_000809605.3_ASM80960v3 |  |  |
| GCF_000809625.3_ASM80962v3 |  |  |
| GCF_000809645.3_ASM80964v3 |  |  |
| GCF_000809665.3_ASM80966v3 |  |  |
| GCF_000809685.3_ASM80968v3 |  |  |
| GCF_000809705.3_ASM80970v3 |  |  |
| GCF_000809725.3_ASM80972v3 |  |  |
| GCF_000809745.3_ASM80974v3 |  |  |
| GCF_000809765.3_ASM80976v3 |  |  |
| GCF_000809785.3_ASM80978v3 |  |  |
| GCF_000809805.3_ASM80980v3 |  |  |
| GCF_000809825.3_ASM80982v3 |  |  |
| GCF_000809845.3_ASM80984v3 |  |  |
| GCF_000809865.3_ASM80986v3 |  |  |
| GCF_000809885.2_ASM80988v3 |  |  |
| GCF_000809905.3_ASM80990v3 |  |  |
| GCF_000809925.3_ASM80992v3 |  |  |
| GCF_000809945.1_ASM80994v3 |  |  |
| GCF_000809965.3_ASM80996v3 |  |  |
| GCF_000809985.3_ASM80998v3 |  |  |
| GCF_000810005.3_ASM81000v3 |  |  |
| GCF_000810025.3_ASM81002v3 |  |  |
| GCF_000810045.3_ASM81004v3 |  |  |
| GCF_000810065.3_ASM81006v3 |  |  |
| GCF_000810085.3_ASM81008v3 |  |  |
| GCF_000810125.3_ASM81012v3 |  |  |
| GCF_000810145.3_ASM81014v3 |  |  |
| GCF_000810165.3_ASM81016v3 |  |  |
| GCF_000810185.3_ASM81018v3 |  |  |
| GCF_000810205.3_Velvet_assembly_of_Acinetobacter_baumannii_UH514_287 | |  |
| GCF_000810245.3_ASM81024v3 |  |  |
| GCF_000810265.3_ASM81026v3 |  |  |
| GCF_000810285.3_ASM81028v3 |  |  |
| GCF_000810305.3_ASM81030v3 |  |  |
| GCF_000810325.3_ASM81032v3 |  |  |
| GCF_000810345.3_ASM81034v3 |  |  |
| GCF_000810365.3_ASM81036v3 |  |  |
| GCF_000810385.3_ASM81038v3 |  |  |
| GCF_000810405.3_ASM81040v3 |  |  |
| GCF_000810425.3_ASM81042v3 |  |  |
| GCF_000810445.3_ASM81044v3 |  |  |
| GCF_000810465.3_ASM81046v3 |  |  |
| GCF_000810485.3_ASM81048v3 |  |  |
| GCF_000810505.3_ASM81050v3 |  |  |
| GCF_000810525.3_ASM81052v3 |  |  |
| GCF_000810545.3_ASM81054v3 |  |  |
| GCF_000810565.3_ASM81056v3 |  |  |
| GCF_000810585.3_ASM81058v3 |  |  |
| GCF_000810605.3_ASM81060v3 |  |  |
| GCF_000810625.2_ASM81062v3 |  |  |
| GCF_000810645.3_ASM81064v3 |  |  |
| GCF_000810665.3_ASM81066v3 |  |  |
| GCF_000810685.3_ASM81068v3 |  |  |
| GCF_000810725.3_ASM81072v3 |  |  |
| GCF_000810745.3_ASM81074v3 |  |  |
| GCF_000810765.3_ASM81076v3 |  |  |
| GCF_000810825.3_ASM81082v3 |  |  |
| GCF_000810835.3_ASM81083v3 |  |  |
| GCF_000810865.3_ASM81086v3 |  |  |
| GCF_000810875.3_ASM81087v3 |  |  |
| GCF_000810905.3_ASM81090v3 |  |  |
| GCF_000810915.3_ASM81091v3 |  |  |
| GCF_000810935.3_ASM81093v3 |  |  |
| GCF_000810945.3_ASM81094v3 |  |  |
| GCF_000810985.3_ASM81098v3 |  |  |
| GCF_000810995.3_ASM81099v3 |  |  |
| GCF_000811005.3_ASM81100v3 |  |  |
| GCF_000811015.3_ASM81101v3 |  |  |
| GCF_000811065.3_ASM81106v3 |  |  |
| GCF_000811085.3_ASM81108v3 |  |  |
| GCF_000811105.3_ASM81110v3 |  |  |
| GCF_000811145.3_ASM81114v3 |  |  |
| GCF_000811165.3_ASM81116v3 |  |  |
| GCF_000811185.3_ASM81118v3 |  |  |
| GCF_000811205.3_ASM81120v3 |  |  |
| GCF_000811225.3_ASM81122v3 |  |  |
| GCF_000811245.3_ASM81124v3 |  |  |
| GCF_000811265.3_ASM81126v3 |  |  |
| GCF_000811285.3_ASM81128v3 |  |  |
| GCF_000811305.3_ASM81130v3 |  |  |
| GCF_000811325.3_ASM81132v3 |  |  |
| GCF_000811345.3_ASM81134v3 |  |  |
| GCF_000811365.3_ASM81136v3 |  |  |
| GCF_000811405.3_ASM81140v3 |  |  |
| GCF_000811485.3_ASM81148v3 |  |  |
| GCF_000811505.3_ASM81150v3 |  |  |
| GCF_000811525.3_ASM81152v3 |  |  |
| GCF_000811545.3_ASM81154v3 |  |  |
| GCF_000811565.3_ASM81156v3 |  |  |
| GCF_000811585.3_ASM81158v3 |  |  |
| GCF_000811605.3_ASM81160v3 |  |  |
| GCF_000811625.3_ASM81162v3 |  |  |
| GCF_000811645.3_ASM81164v3 |  |  |
| GCF_000811665.3_ASM81166v3 |  |  |
| GCF_000811685.3_ASM81168v3 |  |  |
| GCF_000811705.3_ASM81170v3 |  |  |
| GCF_000811725.3_ASM81172v3 |  |  |
| GCF_000811745.3_ASM81174v3 |  |  |
| GCF_000811765.3_ASM81176v3 |  |  |
| GCF_000811785.3_ASM81178v3 |  |  |
| GCF_000811805.3_ASM81180v3 |  |  |
| GCF_000811825.3_ASM81182v3 |  |  |
| GCF_000811865.3_ASM81186v3 |  |  |
| GCF_000811885.3_ASM81188v3 |  |  |
| GCF_000811905.3_ASM81190v3 |  |  |
| GCF_000811925.3_ASM81192v3 |  |  |
| GCF_000811945.3_ASM81194v3 |  |  |
| GCF_000835995.1_ASM83599v1 |  |  |
| GCF_000836075.1_ASM83607v1 |  |  |
| GCF_000876055.1_ASM87605v1 |  |  |
| GCF_000876075.1_ASM87607v1 |  |  |
| GCF_000876095.1_ASM87609v1 |  |  |
| GCF_000931755.1_ASM93175v1 |  |  |
| GCF_000939395.2_ASM93939v2 |  |  |
| GCF_000939405.2_ASM93940v2 |  |  |
| GCF_000939425.2_ASM93942v2 |  |  |
| GCF_000939475.2_ASM93947v2 |  |  |
| GCF_000939485.2_ASM93948v2 |  |  |
| GCF_000939495.2_ASM93949v2 |  |  |
| GCF_000939535.2_ASM93953v2 |  |  |
| GCF_000939555.2_ASM93955v2 |  |  |
| GCF_000939565.2_ASM93956v2 |  |  |
| GCF_000939575.2_ASM93957v2 |  |  |
| GCF_000939615.2_ASM93961v2 |  |  |
| GCF_000939635.2_ASM93963v2 |  |  |
| GCF_000949035.2_ASM94903v2 |  |  |
| GCF_000949045.2_ASM94904v2 |  |  |
| GCF_000949055.2_ASM94905v2 |  |  |
| GCF_000949065.2_ASM94906v2 |  |  |
| GCF_000949115.2_ASM94911v2 |  |  |
| GCF_000949165.2_ASM94916v2 |  |  |
| GCF_000949175.2_ASM94917v2 |  |  |
| GCF_000949185.2_ASM94918v2 |  |  |
| GCF_000949195.2_ASM94919v2 |  |  |
| GCF_000951165.1_ASM95116v1 |  |  |
| GCF_000966305.1_ASM96630v1 |  |  |
| GCF_000966505.1_ASM96650v1 |  |  |
| GCF_000966515.1_ASM96651v1 |  |  |
| GCF_000969285.1_ASM96928v1 |  |  |
| GCF_000969295.1_ASM96929v1 |  |  |
| GCF_000969365.1_ASM96936v1 |  |  |
| GCF_000969465.1_ASM96946v1 |  |  |
| GCF_000969485.1_ASM96948v1 |  |  |
| GCF_000972525.1_ASM97252v1 |  |  |
| GCF_000972625.1_ASM97262v1 |  |  |
| GCF_000988155.1_ASM98815v1 |  |  |
| GCF_001007685.1_ASM100768v1 |  |  |
| GCF_001007705.1_ASM100770v1 |  |  |
| GCF_001007725.1_ASM100772v1 |  |  |
| GCF_001007745.1_ASM100774v1 |  |  |
| GCF_001007765.1_ASM100776v1 |  |  |
| GCF_001007775.1_ASM100777v1 |  |  |
| GCF_001007815.1_ASM100781v1 |  |  |
| GCF_001008125.1_ASM100812v1 |  |  |
| GCF_001027885.1_gdodcwi07_v01 |  |  |
| GCF_001028305.1_gdodcwi04_v01 |  |  |
| GCF_001028325.1_gdodcwi02_v01 |  |  |
| GCF_001028335.1_gdodcwi03_v01 |  |  |
| GCF_001028385.1_gdodcwi01_v01 |  |  |
| GCF_001052015.1_ASM105201v1 |  |  |
| GCF_001052325.1_ASM105232v1 |  |  |
| GCF_001052675.1_ASM105267v1 |  |  |
| GCF_001053695.1_ASM105369v1 |  |  |
| GCF_001053755.1_ASM105375v1 |  |  |
| GCF_001059495.1_ASM105949v1 |  |  |
| GCF_001077575.1_ASM107757v1 |  |  |
| GCF_001276055.1_ASM127605v1 |  |  |
| GCF_001276085.1_ASM127608v1 |  |  |
| GCF_001292715.1_ASM129271v1 |  |  |
| GCF_001400975.1_ASM140097v1 |  |  |
| GCF_001414565.1_ASM141456v1 |  |  |
| GCF_001414575.1_ASM141457v1 |  |  |
| GCF_001414645.1_ASM141464v1 |  |  |
| GCF_001414655.1_ASM141465v1 |  |  |
| GCF_001414685.1_ASM141468v1 |  |  |
| GCF_001414735.1_ASM141473v1 |  |  |
| GCF_001414765.1_ASM141476v1 |  |  |
| GCF_001414785.1_ASM141478v1 |  |  |
| GCF_001414805.1_ASM141480v1 |  |  |
| GCF_001414815.1_ASM141481v1 |  |  |
| GCF_001414865.1_ASM141486v1 |  |  |
| GCF_001414885.1_ASM141488v1 |  |  |
| GCF_001414905.1_ASM141490v1 |  |  |
| GCF_001414915.1_ASM141491v1 |  |  |
| GCF_001414925.1_ASM141492v1 |  |  |
| GCF_001414935.1_ASM141493v1 |  |  |
| GCF_001414985.1_ASM141498v1 |  |  |
| GCF_001414995.1_ASM141499v1 |  |  |
| GCF_001415015.1_ASM141501v1 |  |  |
| GCF_001415025.1_ASM141502v1 |  |  |
| GCF_001415065.1_ASM141506v1 |  |  |
| GCF_001415085.1_ASM141508v1 |  |  |
| GCF_001415095.1_ASM141509v1 |  |  |
| GCF_001415165.1_ASM141516v1 |  |  |
| GCF_001415215.1_ASM141521v1 |  |  |
| GCF_001415255.1_ASM141525v1 |  |  |
| GCF_001415275.1_ASM141527v1 |  |  |
| GCF_001415295.1_ASM141529v1 |  |  |
| GCF_001415325.1_ASM141532v1 |  |  |
| GCF_001415345.1_ASM141534v1 |  |  |
| GCF_001415355.1_ASM141535v1 |  |  |
| GCF_001415385.1_ASM141538v1 |  |  |
| GCF_001415425.1_ASM141542v1 |  |  |
| GCF_001415435.1_ASM141543v1 |  |  |
| GCF_001415465.1_ASM141546v1 |  |  |
| GCF_001415505.1_ASM141550v1 |  |  |
| GCF_001415515.1_ASM141551v1 |  |  |
| GCF_001415535.1_ASM141553v1 |  |  |
| GCF_001415595.1_ASM141559v1 |  |  |
| GCF_001415615.1_ASM141561v1 |  |  |
| GCF_001415645.1_ASM141564v1 |  |  |
| GCF_001415665.1_ASM141566v1 |  |  |
| GCF_001415695.1_ASM141569v1 |  |  |
| GCF_001415715.1_ASM141571v1 |  |  |
| GCF_001415735.1_ASM141573v1 |  |  |
| GCF_001415765.1_ASM141576v1 |  |  |
| GCF_001415785.1_ASM141578v1 |  |  |
| GCF_001415805.1_ASM141580v1 |  |  |
| GCF_001415815.1_ASM141581v1 |  |  |
| GCF_001415835.1_ASM141583v1 |  |  |
| GCF_001415875.1_ASM141587v1 |  |  |
| GCF_001415915.1_ASM141591v1 |  |  |
| GCF_001415965.1_ASM141596v1 |  |  |
| GCF_001415985.1_ASM141598v1 |  |  |
| GCF_001416005.1_ASM141600v1 |  |  |
| GCF_001416015.1_ASM141601v1 |  |  |
| GCF_001416035.1_ASM141603v1 |  |  |
| GCF_001416075.1_ASM141607v1 |  |  |
| GCF_001416095.1_ASM141609v1 |  |  |
| GCF_001416115.1_ASM141611v1 |  |  |
| GCF_001416145.1_ASM141614v1 |  |  |
| GCF_001416155.1_ASM141615v1 |  |  |
| GCF_001416165.1_ASM141616v1 |  |  |
| GCF_001416225.1_ASM141622v1 |  |  |
| GCF_001416235.1_ASM141623v1 |  |  |
| GCF_001416255.1_ASM141625v1 |  |  |
| GCF_001416275.1_ASM141627v1 |  |  |
| GCF_001416305.1_ASM141630v1 |  |  |
| GCF_001416315.1_ASM141631v1 |  |  |
| GCF_001416355.1_ASM141635v1 |  |  |
| GCF_001416385.1_ASM141638v1 |  |  |
| GCF_001416395.1_ASM141639v1 |  |  |
| GCF_001416415.1_ASM141641v1 |  |  |
| GCF_001416425.1_ASM141642v1 |  |  |
| GCF_001416465.1_ASM141646v1 |  |  |
| GCF_001416475.1_ASM141647v1 |  |  |
| GCF_001416495.1_ASM141649v1 |  |  |
| GCF_001416515.1_ASM141651v1 |  |  |
| GCF_001416545.1_ASM141654v1 |  |  |
| GCF_001416555.1_ASM141655v1 |  |  |
| GCF_001416565.1_ASM141656v1 |  |  |
| GCF_001416585.1_ASM141658v1 |  |  |
| GCF_001416625.1_ASM141662v1 |  |  |
| GCF_001416635.1_ASM141663v1 |  |  |
| GCF_001416655.1_ASM141665v1 |  |  |
| GCF_001416665.1_ASM141666v1 |  |  |
| GCF_001416705.1_ASM141670v1 |  |  |
| GCF_001416715.1_ASM141671v1 |  |  |
| GCF_001417485.1_ASM141748v1 |  |  |
| GCF_001417495.1_ASM141749v1 |  |  |
| GCF_001417515.1_ASM141751v1 |  |  |
| GCF_001417545.1_ASM141754v1 |  |  |
| GCF_001432265.1_ASM143226v1 |  |  |
| GCF_001432375.1_ASM143237v1 |  |  |
| GCF_001432385.1_ASM143238v1 |  |  |
| GCF_001432425.1_ASM143242v1 |  |  |
| GCF_001432445.1_ASM143244v1 |  |  |
| GCF_001432455.1_ASM143245v1 |  |  |
| GCF_001432525.1_ASM143252v1 |  |  |
| GCF_001432535.1_ASM143253v1 |  |  |
| GCF_001432545.1_ASM143254v1 |  |  |
| GCF_001432585.1_ASM143258v1 |  |  |
| GCF_001432595.1_ASM143259v1 |  |  |
| GCF_001432625.1_ASM143262v1 |  |  |
| GCF_001432665.1_ASM143266v1 |  |  |
| GCF_001432685.1_ASM143268v1 |  |  |
| GCF_001432705.1_ASM143270v1 |  |  |
| GCF_001432745.1_ASM143274v1 |  |  |
| GCF_001432755.1_ASM143275v1 |  |  |
| GCF_001432785.1_ASM143278v1 |  |  |
| GCF_001432825.1_ASM143282v1 |  |  |
| GCF_001432845.1_ASM143284v1 |  |  |
| GCF_001432895.1_ASM143289v1 |  |  |
| GCF_001432925.1_ASM143292v1 |  |  |
| GCF_001432965.1_ASM143296v1 |  |  |
| GCF_001432975.1_ASM143297v1 |  |  |
| GCF_001433005.1_ASM143300v1 |  |  |
| GCF_001433015.1_ASM143301v1 |  |  |
| GCF_001433045.1_ASM143304v1 |  |  |
| GCF_001433055.1_ASM143305v1 |  |  |
| GCF_001433075.1_ASM143307v1 |  |  |
| GCF_001433145.1_ASM143314v1 |  |  |
| GCF_001433155.1_ASM143315v1 |  |  |
| GCF_001433205.1_ASM143320v1 |  |  |
| GCF_001433245.1_ASM143324v1 |  |  |
| GCF_001433265.1_ASM143326v1 |  |  |
| GCF_001433275.1_ASM143327v1 |  |  |
| GCF_001433335.1_ASM143333v1 |  |  |
| GCF_001433355.1_ASM143335v1 |  |  |
| GCF_001433655.1_ASM143365v1 |  |  |
| GCF_001433675.1_ASM143367v1 |  |  |
| GCF_001433695.1_ASM143369v1 |  |  |
| GCF_001441355.1_ASM144135v1 |  |  |
| GCF_001441405.1_ASM144140v1 |  |  |
| GCF_001441415.1_ASM144141v1 |  |  |
| GCF_001441455.1_ASM144145v1 |  |  |
| GCF_001441465.1_ASM144146v1 |  |  |
| GCF_001441475.1_ASM144147v1 |  |  |
| GCF_001441485.1_ASM144148v1 |  |  |
| GCF_001441545.1_ASM144154v1 |  |  |
| GCF_001441555.1_ASM144155v1 |  |  |
| GCF_001441565.1_ASM144156v1 |  |  |
| GCF_001441625.1_ASM144162v1 |  |  |
| GCF_001441645.1_ASM144164v1 |  |  |
| GCF_001442575.1_ASM144257v1 |  |  |
| GCF_001443265.1_ASM144326v1 |  |  |
| GCF_001444215.1_ASM144421v1 |  |  |
| GCF_001444225.1_ASM144422v1 |  |  |
| GCF_001444255.1_ASM144425v1 |  |  |
| GCF_001444265.1_ASM144426v1 |  |  |
| GCF_001446715.1_ASM144671v1 |  |  |
| GCF_001471855.2_ASM147185v2 |  |  |
| GCF_001500225.1_ASM150022v1 |  |  |
| GCF_001518715.1_ASM151871v1 |  |  |
| GCF_001531135.1_ASM153113v1 |  |  |
| GCF_001531205.2_ASM153120v2 |  |  |
| GCF_001531265.2_ASM153126v2 |  |  |
| GCF_001531455.2_ASM153145v2 |  |  |
| GCF_001541025.2_ASM154102v2 |  |  |
| GCF_001541035.2_ASM154103v2 |  |  |
| GCF_001541045.2_ASM154104v2 |  |  |
| GCF_001544395.1_P630 |  |  |
| GCF_001576615.1_ASM157661v1 |  |  |
| GCF_001617895.1_ASM161789v1 |  |  |
| GCF_001618125.1_ASM161812v1 |  |  |
| GCF_001624975.1_ASM162497v1 |  |  |
| GCF_001640065.1_ASM164006v1 |  |  |
| GCF_001640075.1_ASM164007v1 |  |  |
| GCF_001649595.1_ASM164959v1 |  |  |
| GCF_001649615.1_ASM164961v1 |  |  |
| GCF_001649625.1_ASM164962v1 |  |  |
| GCF_001649675.1_ASM164967v1 |  |  |
| GCF_001649705.1_ASM164970v1 |  |  |
| GCF_001649765.1_ASM164976v1 |  |  |
| GCF_001649795.1_ASM164979v1 |  |  |
| GCF_001649805.1_ASM164980v1 |  |  |
| GCF_001649815.1_ASM164981v1 |  |  |
| GCF_001649835.1_ASM164983v1 |  |  |
| GCF_001649875.1_ASM164987v1 |  |  |
| GCF_001649895.1_ASM164989v1 |  |  |
| GCF_001649955.1_ASM164995v1 |  |  |
| GCF_001649975.1_ASM164997v1 |  |  |
| GCF_001664565.1_ASM166456v1 |  |  |
| GCF_001664575.1_ASM166457v1 |  |  |
| GCF_001664585.1_ASM166458v1 |  |  |
| GCF_001664595.1_ASM166459v1 |  |  |
| GCF_001664645.1_ASM166464v1 |  |  |
| GCF_001664655.1_ASM166465v1 |  |  |
| GCF_001664665.1_ASM166466v1 |  |  |
| GCF_001664685.1_ASM166468v1 |  |  |
| GCF_001664725.1_ASM166472v1 |  |  |
| GCF_001664735.1_ASM166473v1 |  |  |
| GCF_001664755.1_ASM166475v1 |  |  |
| GCF_001664765.1_ASM166476v1 |  |  |
| GCF_001664805.1_ASM166480v1 |  |  |
| GCF_001664815.1_ASM166481v1 |  |  |
| GCF_001664835.1_ASM166483v1 |  |  |
| GCF_001664855.1_ASM166485v1 |  |  |
| GCF_001664885.1_ASM166488v1 |  |  |
| GCF_001664895.1_ASM166489v1 |  |  |
| GCF_001664905.1_ASM166490v1 |  |  |
| GCF_001664915.1_ASM166491v1 |  |  |
| GCF_001664965.1_ASM166496v1 |  |  |
| GCF_001664975.1_ASM166497v1 |  |  |
| GCF_001664985.1_ASM166498v1 |  |  |
| GCF_001665005.1_ASM166500v1 |  |  |
| GCF_001665045.1_ASM166504v1 |  |  |
| GCF_001665055.1_ASM166505v1 |  |  |
| GCF_001665065.1_ASM166506v1 |  |  |
| GCF_001665095.1_ASM166509v1 |  |  |
| GCF_001665125.1_ASM166512v1 |  |  |
| GCF_001665145.1_ASM166514v1 |  |  |
| GCF_001665155.1_ASM166515v1 |  |  |
| GCF_001665175.1_ASM166517v1 |  |  |
| GCF_001665205.1_ASM166520v1 |  |  |
| GCF_001665215.1_ASM166521v1 |  |  |
| GCF_001665285.1_ASM166528v1 |  |  |
| GCF_001665985.1_ASM166598v1 |  |  |
| GCF_001666065.1_ASM166606v1 |  |  |
| GCF_001666075.1_ASM166607v1 |  |  |
| GCF_001666085.1_ASM166608v1 |  |  |
| GCF_001666105.1_ASM166610v1 |  |  |
| GCF_001666145.1_ASM166614v1 |  |  |
| GCF_001666155.1_ASM166615v1 |  |  |
| GCF_001666175.1_ASM166617v1 |  |  |
| GCF_001666195.1_ASM166619v1 |  |  |
| GCF_001666225.1_ASM166622v1 |  |  |
| GCF_001666235.1_ASM166623v1 |  |  |
| GCF_001666255.1_ASM166625v1 |  |  |
| GCF_001666275.1_ASM166627v1 |  |  |
| GCF_001666305.1_ASM166630v1 |  |  |
| GCF_001666315.1_ASM166631v1 |  |  |
| GCF_001666325.1_ASM166632v1 |  |  |
| GCF_001666365.1_ASM166636v1 |  |  |
| GCF_001666385.1_ASM166638v1 |  |  |
| GCF_001666395.1_ASM166639v1 |  |  |
| GCF_001666405.1_ASM166640v1 |  |  |
| GCF_001666425.1_ASM166642v1 |  |  |
| GCF_001666465.1_ASM166646v1 |  |  |
| GCF_001666475.1_ASM166647v1 |  |  |
| GCF_001666485.1_ASM166648v1 |  |  |
| GCF_001666515.1_ASM166651v1 |  |  |
| GCF_001666545.1_ASM166654v1 |  |  |
| GCF_001666555.1_ASM166655v1 |  |  |
| GCF_001666565.1_ASM166656v1 |  |  |
| GCF_001666575.1_ASM166657v1 |  |  |
| GCF_001666625.1_ASM166662v1 |  |  |
| GCF_001666635.1_ASM166663v1 |  |  |
| GCF_001666645.1_ASM166664v1 |  |  |
| GCF_001666665.1_ASM166666v1 |  |  |
| GCF_001666705.1_ASM166670v1 |  |  |
| GCF_001666715.1_ASM166671v1 |  |  |
| GCF_001668445.1_ASM166844v1 |  |  |
| GCF_001668475.1_ASM166847v1 |  |  |
| GCF_001668515.1_ASM166851v1 |  |  |
| GCF_001668535.1_ASM166853v1 |  |  |
| GCF_001668555.1_ASM166855v1 |  |  |
| GCF_001668585.1_ASM166858v1 |  |  |
| GCF_001668645.1_ASM166864v1 |  |  |
| GCF_001668755.1_ASM166875v1 |  |  |
| GCF_001668765.1_ASM166876v1 |  |  |
| GCF_001668775.1_ASM166877v1 |  |  |
| GCF_001668815.1_ASM166881v1 |  |  |
| GCF_001668835.1_ASM166883v1 |  |  |
| GCF_001668855.1_ASM166885v1 |  |  |
| GCF_001668865.1_ASM166886v1 |  |  |
| GCF_001668875.1_ASM166887v1 |  |  |
| GCF_001668915.1_ASM166891v1 |  |  |
| GCF_001668935.1_ASM166893v1 |  |  |
| GCF_001668945.1_ASM166894v1 |  |  |
| GCF_001668955.1_ASM166895v1 |  |  |
| GCF_001668995.1_ASM166899v1 |  |  |
| GCF_001669005.1_ASM166900v1 |  |  |
| GCF_001669025.1_ASM166902v1 |  |  |
| GCF_001669035.1_ASM166903v1 |  |  |
| GCF_001669075.1_ASM166907v1 |  |  |
| GCF_001669095.1_ASM166909v1 |  |  |
| GCF_001669105.1_ASM166910v1 |  |  |
| GCF_001669115.1_ASM166911v1 |  |  |
| GCF_001669175.1_ASM166917v1 |  |  |
| GCF_001669185.1_ASM166918v1 |  |  |
| GCF_001669195.1_ASM166919v1 |  |  |
| GCF_001669225.1_ASM166922v1 |  |  |
| GCF_001669255.1_ASM166925v1 |  |  |
| GCF_001669265.1_ASM166926v1 |  |  |
| GCF_001669295.1_ASM166929v1 |  |  |
| GCF_001669305.1_ASM166930v1 |  |  |
| GCF_001669355.1_ASM166935v1 |  |  |
| GCF_001669365.1_ASM166936v1 |  |  |
| GCF_001669415.1_ASM166941v1 |  |  |
| GCF_001669425.1_ASM166942v1 |  |  |
| GCF_001669435.1_ASM166943v1 |  |  |
| GCF_001669455.1_ASM166945v1 |  |  |
| GCF_001669495.1_ASM166949v1 |  |  |
| GCF_001669505.1_ASM166950v1 |  |  |
| GCF_001669525.1_ASM166952v1 |  |  |
| GCF_001669535.1_ASM166953v1 |  |  |
| GCF_001669575.1_ASM166957v1 |  |  |
| GCF_001669595.1_ASM166959v1 |  |  |
| GCF_001669605.1_ASM166960v1 |  |  |
| GCF_001669635.1_ASM166963v1 |  |  |
| GCF_001669645.1_ASM166964v1 |  |  |
| GCF_001669675.1_ASM166967v1 |  |  |
| GCF_001669695.1_ASM166969v1 |  |  |
| GCF_001669705.1_ASM166970v1 |  |  |
| GCF_001669735.1_ASM166973v1 |  |  |
| GCF_001673795.1_ASM167379v1 |  |  |
| GCF_001673805.1_ASM167380v1 |  |  |
| GCF_001673815.1_ASM167381v1 |  |  |
| GCF_001673825.1_ASM167382v1 |  |  |
| GCF_001673875.1_ASM167387v1 |  |  |
| GCF_001673895.1_ASM167389v1 |  |  |
| GCF_001673905.1_ASM167390v1 |  |  |
| GCF_001673915.1_ASM167391v1 |  |  |
| GCF_001673955.1_ASM167395v1 |  |  |
| GCF_001673975.1_ASM167397v1 |  |  |
| GCF_001673985.1_ASM167398v1 |  |  |
| GCF_001674015.1_ASM167401v1 |  |  |
| GCF_001674035.1_ASM167403v1 |  |  |
| GCF_001674045.1_ASM167404v1 |  |  |
| GCF_001674075.1_ASM167407v1 |  |  |
| GCF_001674085.1_ASM167408v1 |  |  |
| GCF_001674115.1_ASM167411v1 |  |  |
| GCF_001674135.1_ASM167413v1 |  |  |
| GCF_001674155.1_ASM167415v1 |  |  |
| GCF_001674165.1_ASM167416v1 |  |  |
| GCF_001674185.1_ASM167418v1 |  |  |
| GCF_001674215.1_ASM167421v1 |  |  |
| GCF_001674235.1_ASM167423v1 |  |  |
| GCF_001674245.1_ASM167424v1 |  |  |
| GCF_001674255.1_ASM167425v1 |  |  |
| GCF_001674295.1_ASM167429v1 |  |  |
| GCF_001674315.1_ASM167431v1 |  |  |
| GCF_001674325.1_ASM167432v1 |  |  |
| GCF_001674345.1_ASM167434v1 |  |  |
| GCF_001674365.1_ASM167436v1 |  |  |
| GCF_001674395.1_ASM167439v1 |  |  |
| GCF_001674415.1_ASM167441v1 |  |  |
| GCF_001674425.1_ASM167442v1 |  |  |
| GCF_001674435.1_ASM167443v1 |  |  |
| GCF_001674485.1_ASM167448v1 |  |  |
| GCF_001674515.1_ASM167451v1 |  |  |
| GCF_001674555.1_ASM167455v1 |  |  |
| GCF_001674575.1_ASM167457v1 |  |  |
| GCF_001674585.1_ASM167458v1 |  |  |
| GCF_001674605.1_ASM167460v1 |  |  |
| GCF_001674635.1_ASM167463v1 |  |  |
| GCF_001674655.1_ASM167465v1 |  |  |
| GCF_001674665.1_ASM167466v1 |  |  |
| GCF_001674695.1_ASM167469v1 |  |  |
| GCF_001674705.1_ASM167470v1 |  |  |
| GCF_001674735.1_ASM167473v1 |  |  |
| GCF_001674755.1_ASM167475v1 |  |  |
| GCF_001674765.1_ASM167476v1 |  |  |
| GCF_001674785.1_ASM167478v1 |  |  |
| GCF_001674805.1_ASM167480v1 |  |  |
| GCF_001674835.1_ASM167483v1 |  |  |
| GCF_001674845.1_ASM167484v1 |  |  |
| GCF_001674865.1_ASM167486v1 |  |  |
| GCF_001674885.1_ASM167488v1 |  |  |
| GCF_001674915.1_ASM167491v1 |  |  |
| GCF_001675265.1_ASM167526v1 |  |  |
| GCF_001693125.1_ASM169312v1 |  |  |
| GCF_001693185.1_ASM169318v1 |  |  |
| GCF_001693195.1_ASM169319v1 |  |  |
| GCF_001693205.1_ASM169320v1 |  |  |
| GCF_001708025.1_ASM170802v1 |  |  |
| GCF_001756815.1_ASM175681v1 |  |  |
| GCF_001758065.1_ASM175806v1 |  |  |
| GCF_001758085.1_ASM175808v1 |  |  |
| GCF_001758125.1_ASM175812v1 |  |  |
| GCF_001758135.1_ASM175813v1 |  |  |
| GCF_001758165.1_ASM175816v1 |  |  |
| GCF_001855175.1_ASM185517v1 |  |  |
| GCF_001863745.1_ASM186374v1 |  |  |
| GCF_001874685.1_ASM187468v1 |  |  |
| GCF_001874765.1_ASM187476v1 |  |  |
| GCF_001874885.1_ASM187488v1 |  |  |
| GCF_001874915.1_ASM187491v1 |  |  |
| GCF_001891205.1_ASM189120v1 |  |  |
| GCF_001895125.1_ASM189512v1 |  |  |
| GCF_001907125.1_ASM190712v1 |  |  |
| GCF_001910585.1_ASM191058v1 |  |  |
| GCF_001910595.1_ASM191059v1 |  |  |
| GCF_001910605.1_ASM191060v1 |  |  |
| GCF_001910615.1_ASM191061v1 |  |  |
| GCF_001910665.1_ASM191066v1 |  |  |
| GCF_001910675.1_ASM191067v1 |  |  |
| GCF_001910685.1_ASM191068v1 |  |  |
| GCF_001922635.1_ASM192263v1 |  |  |
| GCF_001922685.1_ASM192268v1 |  |  |
| GCF_001922695.1_ASM192269v1 |  |  |
| GCF_001922705.1_ASM192270v1 |  |  |
| GCF_001922745.1_ASM192274v1 |  |  |
| GCF_001922755.1_ASM192275v1 |  |  |
| GCF_001923005.1_ASM192300v1 |  |  |
| GCF_001939595.1_ASM193959v1 |  |  |
| GCF_001939605.1_ASM193960v1 |  |  |
| GCF_001939665.1_ASM193966v1 |  |  |
| GCF_001949955.1_ASM194995v1 |  |  |
| GCF_001949965.1_ASM194996v1 |  |  |
| GCF_001950085.1_ASM195008v1 |  |  |
| GCF_001950095.1_ASM195009v1 |  |  |
| GCF_001950155.1_ASM195015v1 |  |  |
| GCF_001950265.1_ASM195026v1 |  |  |
| GCF_001950295.1_ASM195029v1 |  |  |
| GCF_001950315.1_ASM195031v1 |  |  |
| GCF_002006635.1_ASM200663v1 |  |  |
| GCF_002006655.1_ASM200665v1 |  |  |
| GCF_002006665.1_ASM200666v1 |  |  |
| GCF_002014105.1_ASM201410v1 |  |  |
| GCF_002014155.1_ASM201415v1 |  |  |
| GCF_002014175.1_ASM201417v1 |  |  |
| GCF_002014195.1_ASM201419v1 |  |  |
| GCF_002014235.1_ASM201423v1 |  |  |
| GCF_002014245.1_ASM201424v1 |  |  |
| GCF_002014265.1_ASM201426v1 |  |  |
| GCF_002014275.1_ASM201427v1 |  |  |
| GCF_002014315.1_ASM201431v1 |  |  |
| GCF_002014325.1_ASM201432v1 |  |  |
| GCF_002014345.1_ASM201434v1 |  |  |
| GCF_002014355.1_ASM201435v1 |  |  |
| GCF_002014395.1_ASM201439v1 |  |  |
| GCF_002014405.1_ASM201440v1 |  |  |
| GCF_002014435.1_ASM201443v1 |  |  |
| GCF_002014445.1_ASM201444v1 |  |  |
| GCF_002014475.1_ASM201447v1 |  |  |
| GCF_002016655.1_ASM201665v1 |  |  |
| GCF_002016735.1_ASM201673v1 |  |  |
| GCF_002016745.1_ASM201674v1 |  |  |
| GCF_002016775.1_ASM201677v1 |  |  |
| GCF_002016785.1_ASM201678v1 |  |  |
| GCF_002016815.1_ASM201681v1 |  |  |
| GCF_002016825.1_ASM201682v1 |  |  |
| GCF_002016855.1_ASM201685v1 |  |  |
| GCF_002016865.1_ASM201686v1 |  |  |
| GCF_002016915.1_ASM201691v1 |  |  |
| GCF_002016935.1_ASM201693v1 |  |  |
| GCF_002016955.1_ASM201695v1 |  |  |
| GCF_002016975.1_ASM201697v1 |  |  |
| GCF_002016985.1_ASM201698v1 |  |  |
| GCF_002016995.1_ASM201699v1 |  |  |
| GCF_002017035.1_ASM201703v1 |  |  |
| GCF_002017055.1_ASM201705v1 |  |  |
| GCF_002017065.1_ASM201706v1 |  |  |
| GCF_002017095.1_ASM201709v1 |  |  |
| GCF_002017105.1_ASM201710v1 |  |  |
| GCF_002017115.1_ASM201711v1 |  |  |
| GCF_002017125.1_ASM201712v1 |  |  |
| GCF_002017175.1_ASM201717v1 |  |  |
| GCF_002017185.1_ASM201718v1 |  |  |
| GCF_002017195.1_ASM201719v1 |  |  |
| GCF_002017205.1_ASM201720v1 |  |  |
| GCF_002017255.1_ASM201725v1 |  |  |
| GCF_002017265.1_ASM201726v1 |  |  |
| GCF_002017285.1_ASM201728v1 |  |  |
| GCF_002017315.1_ASM201731v1 |  |  |
| GCF_002017335.1_ASM201733v1 |  |  |
| GCF_002017345.1_ASM201734v1 |  |  |
| GCF_002017355.1_ASM201735v1 |  |  |
| GCF_002017395.1_ASM201739v1 |  |  |
| GCF_002017405.1_ASM201740v1 |  |  |
| GCF_002017415.1_ASM201741v1 |  |  |
| GCF_002017425.1_ASM201742v1 |  |  |
| GCF_002017475.1_ASM201747v1 |  |  |
| GCF_002017485.1_ASM201748v1 |  |  |
| GCF_002017515.1_ASM201751v1 |  |  |
| GCF_002017525.1_ASM201752v1 |  |  |
| GCF_002017555.1_ASM201755v1 |  |  |
| GCF_002017565.1_ASM201756v1 |  |  |
| GCF_002017595.1_ASM201759v1 |  |  |
| GCF_002017605.1_ASM201760v1 |  |  |
| GCF_002017615.1_ASM201761v1 |  |  |
| GCF_002017625.1_ASM201762v1 |  |  |
| GCF_002017675.1_ASM201767v1 |  |  |
| GCF_002017685.1_ASM201768v1 |  |  |
| GCF_002017705.1_ASM201770v1 |  |  |
| GCF_002017735.1_ASM201773v1 |  |  |
| GCF_002017745.1_ASM201774v1 |  |  |
| GCF_002017765.1_ASM201776v1 |  |  |
| GCF_002017795.1_ASM201779v1 |  |  |
| GCF_002017805.1_ASM201780v1 |  |  |
| GCF_002018935.1_ASM201893v1 |  |  |
| GCF_002018965.1_ASM201896v1 |  |  |
| GCF_002018995.1_ASM201899v1 |  |  |
| GCF_002019005.1_ASM201900v1 |  |  |
| GCF_002019025.1_ASM201902v1 |  |  |
| GCF_002019035.1_ASM201903v1 |  |  |
| GCF_002028445.1_ASM202844v1 |  |  |
| GCF_002028475.1_ASM202847v1 |  |  |
| GCF_002028485.1_ASM202848v1 |  |  |
| GCF_002028535.1_ASM202853v1 |  |  |
| GCF_002085505.1_ASM208550v1 |  |  |
| GCF_002119275.1_ASM211927v1 |  |  |
| GCF_002119285.1_ASM211928v1 |  |  |
| GCF_002119295.1_ASM211929v1 |  |  |
| GCF_002136595.1_ASM213659v1 |  |  |
| GCF_002136605.1_ASM213660v1 |  |  |
| GCF_002136615.1_ASM213661v1 |  |  |
| GCF_002136655.1_ASM213665v1 |  |  |
| GCF_002136675.1_ASM213667v1 |  |  |
| GCF_002136685.1_ASM213668v1 |  |  |
| GCF_002136705.1_ASM213670v1 |  |  |
| GCF_002136735.1_ASM213673v1 |  |  |
| GCF_002136745.1_ASM213674v1 |  |  |
| GCF_002136775.1_ASM213677v1 |  |  |
| GCF_002136815.1_ASM213681v1 |  |  |
| GCF_002136825.1_ASM213682v1 |  |  |
| GCF_002136855.1_ASM213685v1 |  |  |
| GCF_002136885.1_ASM213688v1 |  |  |
| GCF_002136895.1_ASM213689v1 |  |  |
| GCF_002136935.1_ASM213693v1 |  |  |
| GCF_002136955.1_ASM213695v1 |  |  |
| GCF_002136965.1_ASM213696v1 |  |  |
| GCF_002136995.1_ASM213699v1 |  |  |
| GCF_002137035.1_ASM213703v1 |  |  |
| GCF_002137045.1_ASM213704v1 |  |  |
| GCF_002137185.1_ASM213718v1 |  |  |
| GCF_002137215.1_ASM213721v1 |  |  |
| GCF_002137225.1_ASM213722v1 |  |  |
| GCF_002137255.1_ASM213725v1 |  |  |
| GCF_002137265.1_ASM213726v1 |  |  |
| GCF_002137285.1_ASM213728v1 |  |  |
| GCF_002137295.1_ASM213729v1 |  |  |
| GCF_002137335.1_ASM213733v1 |  |  |
| GCF_002137345.1_ASM213734v1 |  |  |
| GCF_002137395.1_ASM213739v1 |  |  |
| GCF_002137425.1_ASM213742v1 |  |  |
| GCF_002137445.1_ASM213744v1 |  |  |
| GCF_002137475.1_ASM213747v1 |  |  |
| GCF_002137495.1_ASM213749v1 |  |  |
| GCF_002137525.1_ASM213752v1 |  |  |
| GCF_002137555.1_ASM213755v1 |  |  |
| GCF_002137565.1_ASM213756v1 |  |  |
| GCF_002137575.1_ASM213757v1 |  |  |
| GCF_002137645.1_ASM213764v1 |  |  |
| GCF_002137655.1_ASM213765v1 |  |  |
| GCF_002137695.1_ASM213769v1 |  |  |
| GCF_002137715.1_ASM213771v1 |  |  |
| GCF_002137725.1_ASM213772v1 |  |  |
| GCF_002137755.1_ASM213775v1 |  |  |
| GCF_002137775.1_ASM213777v1 |  |  |
| GCF_002137795.1_ASM213779v1 |  |  |
| GCF_002137815.1_ASM213781v1 |  |  |
| GCF_002137875.1_ASM213787v1 |  |  |
| GCF_002137895.1_ASM213789v1 |  |  |
| GCF_002137945.1_ASM213794v1 |  |  |
| GCF_002137955.1_ASM213795v1 |  |  |
| GCF_002137995.1_ASM213799v1 |  |  |
| GCF_002138005.1_ASM213800v1 |  |  |
| GCF_002138035.1_ASM213803v1 |  |  |
| GCF_002138055.1_ASM213805v1 |  |  |
| GCF_002138065.1_ASM213806v1 |  |  |
| GCF_002138105.1_ASM213810v1 |  |  |
| GCF_002138115.1_ASM213811v1 |  |  |
| GCF_002138155.1_ASM213815v1 |  |  |
| GCF_002138165.1_ASM213816v1 |  |  |
| GCF_002138175.1_ASM213817v1 |  |  |
| GCF_002138215.1_ASM213821v1 |  |  |
| GCF_002138245.1_ASM213824v1 |  |  |
| GCF_002138295.1_ASM213829v1 |  |  |
| GCF_002138305.1_ASM213830v1 |  |  |
| GCF_002138315.1_ASM213831v1 |  |  |
| GCF_002138345.1_ASM213834v1 |  |  |
| GCF_002138375.1_ASM213837v1 |  |  |
| GCF_002142735.1_ASM214273v1 |  |  |
| GCF_002142935.1_ASM214293v1 |  |  |
| GCF_002142945.1_ASM214294v1 |  |  |
| GCF_002142955.1_ASM214295v1 |  |  |
| GCF_002142995.1_ASM214299v1 |  |  |
| GCF_002143015.1_ASM214301v1 |  |  |
| GCF_002143035.1_ASM214303v1 |  |  |
| GCF_002143045.1_ASM214304v1 |  |  |
| GCF_002143095.1_ASM214309v1 |  |  |
| GCF_002143105.1_ASM214310v1 |  |  |
| GCF_002143145.1_ASM214314v1 |  |  |
| GCF_002143185.1_ASM214318v1 |  |  |
| GCF_002143195.1_ASM214319v1 |  |  |
| GCF_002143235.1_ASM214323v1 |  |  |
| GCF_002143265.1_ASM214326v1 |  |  |
| GCF_002143275.1_ASM214327v1 |  |  |
| GCF_002143315.1_ASM214331v1 |  |  |
| GCF_002143335.1_ASM214333v1 |  |  |
| GCF_002143345.1_ASM214334v1 |  |  |
| GCF_002143375.1_ASM214337v1 |  |  |
| GCF_002143395.1_ASM214339v1 |  |  |
| GCF_002143405.1_ASM214340v1 |  |  |
| GCF_002143425.1_ASM214342v1 |  |  |
| GCF_002143475.1_ASM214347v1 |  |  |
| GCF_002143495.1_ASM214349v1 |  |  |
| GCF_002143555.1_ASM214355v1 |  |  |
| GCF_002143575.1_ASM214357v1 |  |  |
| GCF_002143605.1_ASM214360v1 |  |  |
| GCF_002143625.1_ASM214362v1 |  |  |
| GCF_002143655.1_ASM214365v1 |  |  |
| GCF_002143675.1_ASM214367v1 |  |  |
| GCF_002143685.1_ASM214368v1 |  |  |
| GCF_002143715.1_ASM214371v1 |  |  |
| GCF_002143725.1_ASM214372v1 |  |  |
| GCF_002143755.1_ASM214375v1 |  |  |
| GCF_002143775.1_ASM214377v1 |  |  |
| GCF_002143785.1_ASM214378v1 |  |  |
| GCF_002143795.1_ASM214379v1 |  |  |
| GCF_002143835.1_ASM214383v1 |  |  |
| GCF_002143855.1_ASM214385v1 |  |  |
| GCF_002143875.1_ASM214387v1 |  |  |
| GCF_002143885.1_ASM214388v1 |  |  |
| GCF_002143935.1_ASM214393v1 |  |  |
| GCF_002143955.1_ASM214395v1 |  |  |
| GCF_002143975.1_ASM214397v1 |  |  |
| GCF_002144685.1_ASM214468v1 |  |  |
| GCF_002144695.1_ASM214469v1 |  |  |
| GCF_002144725.1_ASM214472v1 |  |  |
| GCF_002144755.1_ASM214475v1 |  |  |
| GCF_002144765.1_ASM214476v1 |  |  |
| GCF_002144795.1_ASM214479v1 |  |  |
| GCF_002144805.1_ASM214480v1 |  |  |
| GCF_002144835.1_ASM214483v1 |  |  |
| GCF_002144925.1_ASM214492v1 |  |  |
| GCF_002144955.1_ASM214495v1 |  |  |
| GCF_002144965.1_ASM214496v1 |  |  |
| GCF_002144975.1_ASM214497v1 |  |  |
| GCF_002144995.1_ASM214499v1 |  |  |
| GCF_002145035.1_ASM214503v1 |  |  |
| GCF_002145055.1_ASM214505v1 |  |  |
| GCF_002145065.1_ASM214506v1 |  |  |
| GCF_002145095.1_ASM214509v1 |  |  |
| GCF_002145115.1_ASM214511v1 |  |  |
| GCF_002145125.1_ASM214512v1 |  |  |
| GCF_002145155.1_ASM214515v1 |  |  |
| GCF_002145175.1_ASM214517v1 |  |  |
| GCF_002145195.1_ASM214519v1 |  |  |
| GCF_002145255.1_ASM214525v1 |  |  |
| GCF_002145295.1_ASM214529v1 |  |  |
| GCF_002145375.1_ASM214537v1 |  |  |
| GCF_002145395.1_ASM214539v1 |  |  |
| GCF_002145435.1_ASM214543v1 |  |  |
| GCF_002148725.1_ASM214872v1 |  |  |
| GCF_002148735.1_ASM214873v1 |  |  |
| GCF_002148745.1_ASM214874v1 |  |  |
| GCF_002150405.1_ASM215040v1 |  |  |
| GCF_002154155.1_ASM215415v1 |  |  |
| GCF_002154215.1_ASM215421v1 |  |  |
| GCF_002182345.1_ASM218234v1 |  |  |
| GCF_002182365.1_ASM218236v1 |  |  |
| GCF_002182385.1_ASM218238v1 |  |  |
| GCF_002182405.1_ASM218240v1 |  |  |
| GCF_002182425.1_ASM218242v1 |  |  |
| GCF_002182445.1_ASM218244v1 |  |  |
| GCF_002182465.1_ASM218246v1 |  |  |
| GCF_002182485.1_ASM218248v1 |  |  |
| GCF_002182505.1_ASM218250v1 |  |  |
| GCF_002182545.1_ASM218254v1 |  |  |
| GCF_002182605.1_ASM218260v1 |  |  |
| GCF_002182615.1_ASM218261v1 |  |  |
| GCF_002182625.1_ASM218262v1 |  |  |
| GCF_002182645.1_ASM218264v1 |  |  |
| GCF_002182685.1_ASM218268v1 |  |  |
| GCF_002182695.1_ASM218269v1 |  |  |
| GCF_002182705.1_ASM218270v1 |  |  |
| GCF_002182725.1_ASM218272v1 |  |  |
| GCF_002182765.1_ASM218276v1 |  |  |
| GCF_002182785.1_ASM218278v1 |  |  |
| GCF_002182795.1_ASM218279v1 |  |  |
| GCF_002182805.1_ASM218280v1 |  |  |
| GCF_002182845.1_ASM218284v1 |  |  |
| GCF_002182865.1_ASM218286v1 |  |  |
| GCF_002182875.1_ASM218287v1 |  |  |
| GCF_002182895.1_ASM218289v1 |  |  |
| GCF_002182925.1_ASM218292v1 |  |  |
| GCF_002182945.1_ASM218294v1 |  |  |
| GCF_002182955.1_ASM218295v1 |  |  |
| GCF_002182965.1_ASM218296v1 |  |  |
| GCF_002183005.1_ASM218300v1 |  |  |
| GCF_002183025.1_ASM218302v1 |  |  |
| GCF_002183035.1_ASM218303v1 |  |  |
| GCF_002183045.1_ASM218304v1 |  |  |
| GCF_002183085.1_ASM218308v1 |  |  |
| GCF_002183105.1_ASM218310v1 |  |  |
| GCF_002183115.1_ASM218311v1 |  |  |
| GCF_002183125.1_ASM218312v1 |  |  |
| GCF_002183165.1_ASM218316v1 |  |  |
| GCF_002183185.1_ASM218318v1 |  |  |
| GCF_002183195.1_ASM218319v1 |  |  |
| GCF_002183215.1_ASM218321v1 |  |  |
| GCF_002183245.1_ASM218324v1 |  |  |
| GCF_002183265.1_ASM218326v1 |  |  |
| GCF_002183275.1_ASM218327v1 |  |  |
| GCF_002183285.1_ASM218328v1 |  |  |
| GCF_002183325.1_ASM218332v1 |  |  |
| GCF_002183345.1_ASM218334v1 |  |  |
| GCF_002183355.1_ASM218335v1 |  |  |
| GCF_002183365.1_ASM218336v1 |  |  |
| GCF_002183405.1_ASM218340v1 |  |  |
| GCF_002183425.1_ASM218342v1 |  |  |
| GCF_002183435.1_ASM218343v1 |  |  |
| GCF_002183445.1_ASM218344v1 |  |  |
| GCF_002183485.1_ASM218348v1 |  |  |
| GCF_002183505.1_ASM218350v1 |  |  |
| GCF_002183515.1_ASM218351v1 |  |  |
| GCF_002183545.1_ASM218354v1 |  |  |
| GCF_002183555.1_ASM218355v1 |  |  |
| GCF_002183585.1_ASM218358v1 |  |  |
| GCF_002183595.1_ASM218359v1 |  |  |
| GCF_002183625.1_ASM218362v1 |  |  |
| GCF_002183635.1_ASM218363v1 |  |  |
| GCF_002183665.1_ASM218366v1 |  |  |
| GCF_002183675.1_ASM218367v1 |  |  |
| GCF_002183705.1_ASM218370v1 |  |  |
| GCF_002183725.1_ASM218372v1 |  |  |
| GCF_002183735.1_ASM218373v1 |  |  |
| GCF_002183765.1_ASM218376v1 |  |  |
| GCF_002183785.1_ASM218378v1 |  |  |
| GCF_002183805.1_ASM218380v1 |  |  |
| GCF_002183815.1_ASM218381v1 |  |  |
| GCF_002183845.1_ASM218384v1 |  |  |
| GCF_002183855.1_ASM218385v1 |  |  |
| GCF_002183875.1_ASM218387v1 |  |  |
| GCF_002183905.1_ASM218390v1 |  |  |
| GCF_002183925.1_ASM218392v1 |  |  |
| GCF_002183945.1_ASM218394v1 |  |  |
| GCF_002183955.1_ASM218395v1 |  |  |
| GCF_002183975.1_ASM218397v1 |  |  |
| GCF_002184005.1_ASM218400v1 |  |  |
| GCF_002184025.1_ASM218402v1 |  |  |
| GCF_002184035.1_ASM218403v1 |  |  |
| GCF_002184055.1_ASM218405v1 |  |  |
| GCF_002184085.1_ASM218408v1 |  |  |
| GCF_002184105.1_ASM218410v1 |  |  |
| GCF_002184265.1_ASM218426v1 |  |  |
| GCF_002184275.1_ASM218427v1 |  |  |
| GCF_002184285.1_ASM218428v1 |  |  |
| GCF_002184325.1_ASM218432v1 |  |  |
| GCF_002184335.1_ASM218433v1 |  |  |
| GCF_002184365.1_ASM218436v1 |  |  |
| GCF_002184385.1_ASM218438v1 |  |  |
| GCF_002184565.1_ASM218456v1 |  |  |
| GCF_002184605.1_ASM218460v1 |  |  |
| GCF_002197745.1_ASM219774v1 |  |  |
| GCF_002212105.1_ASM221210v1 |  |  |
| GCF_002212115.1_ASM221211v1 |  |  |
| GCF_002212145.1_ASM221214v1 |  |  |
| GCF_002212155.1_ASM221215v1 |  |  |
| GCF_002212165.1_ASM221216v1 |  |  |
| GCF_002212225.1_ASM221222v1 |  |  |
| GCF_002212235.1_ASM221223v1 |  |  |
| GCF_002212245.1_ASM221224v1 |  |  |
| GCF_002212285.1_ASM221228v1 |  |  |
| GCF_002212305.1_ASM221230v1 |  |  |
| GCF_002212315.1_ASM221231v1 |  |  |
| GCF_002212325.1_ASM221232v1 |  |  |
| GCF_002212365.1_ASM221236v1 |  |  |
| GCF_002212385.1_ASM221238v1 |  |  |
| GCF_002212405.1_ASM221240v1 |  |  |
| GCF_002212445.1_ASM221244v1 |  |  |
| GCF_002212465.1_ASM221246v1 |  |  |
| GCF_002212475.1_ASM221247v1 |  |  |
| GCF_002212485.1_ASM221248v1 |  |  |
| GCF_002212525.1_ASM221252v1 |  |  |
| GCF_002212535.1_ASM221253v1 |  |  |
| GCF_002212565.1_ASM221256v1 |  |  |
| GCF_002212605.1_ASM221260v1 |  |  |
| GCF_002212625.1_ASM221262v1 |  |  |
| GCF_002212635.1_ASM221263v1 |  |  |
| GCF_002212645.1_ASM221264v1 |  |  |
| GCF_002212685.1_ASM221268v1 |  |  |
| GCF_002212705.1_ASM221270v1 |  |  |
| GCF_002212715.1_ASM221271v1 |  |  |
| GCF_002239725.1_ASM223972v1 |  |  |
| GCF_002239735.1_ASM223973v1 |  |  |
| GCF_002239865.1_ASM223986v1 |  |  |
| GCF_002239885.1_ASM223988v1 |  |  |
| GCF_002241095.1_ASM224109v1 |  |  |
| GCF_002241105.1_ASM224110v1 |  |  |
| GCF_002249135.1_ASM224913v1 |  |  |
| GCF_002249185.1_ASM224918v1 |  |  |
| GCF_002249225.1_ASM224922v1 |  |  |
| GCF_002249245.1_ASM224924v1 |  |  |
| GCF_002249255.1_ASM224925v1 |  |  |
| GCF_002249265.1_ASM224926v1 |  |  |
| GCF_002249305.1_ASM224930v1 |  |  |
| GCF_002249325.1_ASM224932v1 |  |  |
| GCF_002249335.1_ASM224933v1 |  |  |
| GCF_002249365.1_ASM224936v1 |  |  |
| GCF_002249385.1_ASM224938v1 |  |  |
| GCF_002249405.1_ASM224940v1 |  |  |
| GCF_002249425.1_ASM224942v1 |  |  |
| GCF_002249435.1_ASM224943v1 |  |  |
| GCF_002249465.1_ASM224946v1 |  |  |
| GCF_002249475.1_ASM224947v1 |  |  |
| GCF_002249505.1_ASM224950v1 |  |  |
| GCF_002249525.1_ASM224952v1 |  |  |
| GCF_002249545.1_ASM224954v1 |  |  |
| GCF_002249585.1_ASM224958v1 |  |  |
| GCF_002249605.1_ASM224960v1 |  |  |
| GCF_002249655.1_ASM224965v1 |  |  |
| GCF_002250465.1_ASM225046v1 |  |  |
| GCF_002261505.1_ASM226150v1 |  |  |
| GCF_002261535.1_ASM226153v1 |  |  |
| GCF_002265555.1_ASM226555v1 |  |  |
| GCF_002265675.1_ASM226567v1 |  |  |
| GCF_002367895.1_ASM236789v1 |  |  |
| GCF_002411775.1_ASM241177v1 |  |  |
| GCF_002416325.1_ASM241632v1 |  |  |
| GCF_002416345.1_ASM241634v1 |  |  |
| GCF_002416365.1_ASM241636v1 |  |  |
| GCF_002416375.1_ASM241637v1 |  |  |
| GCF_002416395.1_ASM241639v1 |  |  |
| GCF_002416425.1_ASM241642v1 |  |  |
| GCF_002416435.1_ASM241643v1 |  |  |
| GCF_002416475.1_ASM241647v1 |  |  |
| GCF_002573725.1_ASM257372v1 |  |  |
| GCF_002573735.1_ASM257373v1 |  |  |
| GCF_002573805.1_ASM257380v1 |  |  |
| GCF_002573825.1_ASM257382v1 |  |  |
| GCF_002573875.1_ASM257387v1 |  |  |
| GCF_002573895.1_ASM257389v1 |  |  |
| GCF_002573915.1_ASM257391v1 |  |  |
| GCF_002724085.1_ASM272408v1 |  |  |
| GCF_002760795.1_ASM276079v1 |  |  |
| GCF_002760895.1_ASM276089v1 |  |  |
| GCF_002762495.1_ASM276249v1 |  |  |
| GCF_002762505.1_ASM276250v1 |  |  |
| GCF_002762535.1_ASM276253v1 |  |  |
| GCF_002837005.1_ASM283700v1 |  |  |
| GCF_002837035.1_ASM283703v1 |  |  |
| GCF_002885855.1_ASM288585v1 |  |  |
| GCF_002922935.1_ASM292293v1 |  |  |
| GCF_002927915.1_ASM292791v1 |  |  |
| GCF_002928075.1_ASM292807v1 |  |  |
| GCF_002928095.1_ASM292809v1 |  |  |
| GCF_002950415.1_ASM295041v1 |  |  |
| GCF_002950425.1_ASM295042v1 |  |  |
| GCF_002950435.1_ASM295043v1 |  |  |
| GCF_002950455.1_ASM295045v1 |  |  |
| GCF_002950595.1_ASM295059v1 |  |  |
| GCF_002950625.1_ASM295062v1 |  |  |
| GCF_002950715.1_ASM295071v1 |  |  |
| GCF_002950725.1_ASM295072v1 |  |  |
| GCF_002950815.1_ASM295081v1 |  |  |
| GCF_002950825.1_ASM295082v1 |  |  |
| GCF_002950855.1_ASM295085v1 |  |  |
| GCF_002950895.1_ASM295089v1 |  |  |
| GCF_002950925.1_ASM295092v1 |  |  |
| GCF_002951055.1_ASM295105v1 |  |  |
| GCF_002951075.1_ASM295107v1 |  |  |
| GCF_002951095.1_ASM295109v1 |  |  |
| GCF_002951155.1_ASM295115v1 |  |  |
| GCF_002951215.1_ASM295121v1 |  |  |
| GCF_002951255.1_ASM295125v1 |  |  |
| GCF_002951275.1_ASM295127v1 |  |  |
| GCF_002951345.1_ASM295134v1 |  |  |
| GCF_002951375.1_ASM295137v1 |  |  |
| GCF_002951435.1_ASM295143v1 |  |  |
| GCF_002992205.1_ASM299220v1 |  |  |
| GCF_002992225.1_ASM299222v1 |  |  |
| GCF_002992235.1_ASM299223v1 |  |  |
| GCF_002992255.1_ASM299225v1 |  |  |
| GCF_002992285.1_ASM299228v1 |  |  |
| GCF_002992305.1_ASM299230v1 |  |  |
| GCF_002992325.1_ASM299232v1 |  |  |
| GCF_002992345.1_ASM299234v1 |  |  |
| GCF_002992365.1_ASM299236v1 |  |  |
| GCF_002992385.1_ASM299238v1 |  |  |
| GCF_002992405.1_ASM299240v1 |  |  |
| GCF_002992425.1_ASM299242v1 |  |  |
| GCF_002992445.1_ASM299244v1 |  |  |
| GCF_002992465.1_ASM299246v1 |  |  |
| GCF_002992485.1_ASM299248v1 |  |  |
| GCF_002992505.1_ASM299250v1 |  |  |
| GCF_002992525.1_ASM299252v1 |  |  |
| GCF_002992545.1_ASM299254v1 |  |  |
| GCF_002992565.1_ASM299256v1 |  |  |
| GCF_002992585.1_ASM299258v1 |  |  |
| GCF_002992605.1_ASM299260v1 |  |  |
| GCF_002992625.1_ASM299262v1 |  |  |
| GCF_002992645.1_ASM299264v1 |  |  |
| GCF_002992665.1_ASM299266v1 |  |  |
| GCF_002992685.1_ASM299268v1 |  |  |
| GCF_002992705.1_ASM299270v1 |  |  |
| GCF_002992715.1_ASM299271v1 |  |  |
| GCF_002992745.1_ASM299274v1 |  |  |
| GCF_002992765.1_ASM299276v1 |  |  |
| GCF_002992775.1_ASM299277v1 |  |  |
| GCF_002992805.1_ASM299280v1 |  |  |
| GCF_002992825.1_ASM299282v1 |  |  |
| GCF_002992845.1_ASM299284v1 |  |  |
| GCF_002992865.1_ASM299286v1 |  |  |
| GCF_002992875.1_ASM299287v1 |  |  |
| GCF_002992905.1_ASM299290v1 |  |  |
| GCF_003006885.1_ASM300688v1 |  |  |
| GCF_003012695.1_ASM301269v1 |  |  |
| GCF_003020265.1_ASM302026v1 |  |  |
| GCF_003020275.1_ASM302027v1 |  |  |
| GCF_003020285.1_ASM302028v1 |  |  |
| GCF_003020325.1_ASM302032v1 |  |  |
| GCF_003020345.1_ASM302034v1 |  |  |
| GCF_003020385.1_ASM302038v1 |  |  |
| GCF_003020445.1_ASM302044v1 |  |  |
| GCF_003020455.1_ASM302045v1 |  |  |
| GCF_003020485.1_ASM302048v1 |  |  |
| GCF_003020505.1_ASM302050v1 |  |  |
| GCF_003020525.1_ASM302052v1 |  |  |
| GCF_003020545.1_ASM302054v1 |  |  |
| GCF_003020565.1_ASM302056v1 |  |  |
| GCF_003020575.1_ASM302057v1 |  |  |
| GCF_003038935.1_ASM303893v1 |  |  |
| GCF_003038965.1_ASM303896v1 |  |  |
| GCF_003039005.1_ASM303900v1 |  |  |
| GCF_003039035.1_ASM303903v1 |  |  |
| GCF_003039055.1_ASM303905v1 |  |  |
| GCF_003039075.1_ASM303907v1 |  |  |
| GCF_003039095.1_ASM303909v1 |  |  |
| GCF_003039115.1_ASM303911v1 |  |  |
| GCF_003039135.1_ASM303913v1 |  |  |
| GCF_003039155.1_ASM303915v1 |  |  |
| GCF_003039175.1_ASM303917v1 |  |  |
| GCF_003039195.1_ASM303919v1 |  |  |
| GCF_003039215.1_ASM303921v1 |  |  |
| GCF_003039235.1_ASM303923v1 |  |  |
| GCF_003039255.1_ASM303925v1 |  |  |
| GCF_003039275.1_ASM303927v1 |  |  |
| GCF_003039295.1_ASM303929v1 |  |  |
| GCF_003039315.1_ASM303931v1 |  |  |
| GCF_003039335.1_ASM303933v1 |  |  |
| GCF_003039345.1_ASM303934v1 |  |  |
| GCF_003039375.1_ASM303937v1 |  |  |
| GCF_003039395.1_ASM303939v1 |  |  |
| GCF_003039415.1_ASM303941v1 |  |  |
| GCF_003039475.1_ASM303947v1 |  |  |
| GCF_003057525.1_ASM305752v1 |  |  |
| GCF_003130565.1_ASM313056v1 |  |  |
| GCF_003194535.1_ASM319453v1 |  |  |
| GCF_003325635.1_ASM332563v1 |  |  |
| GCF_003336125.1_ASM333612v1 |  |  |
| GCF_003345795.1_ASM334579v1 |  |  |
| GCF_003345805.1_ASM334580v1 |  |  |
| GCF_003345835.1_ASM334583v1 |  |  |
| GCF_003345855.1_ASM334585v1 |  |  |
| GCF_003345865.1_ASM334586v1 |  |  |
| GCF_003345915.1_ASM334591v1 |  |  |
| GCF_003345925.1_ASM334592v1 |  |  |
| GCF_003345955.1_ASM334595v1 |  |  |
| GCF_003345995.1_ASM334599v1 |  |  |
| GCF_003346005.1_ASM334600v1 |  |  |
| GCF_003347295.1_ASM334729v1 |  |  |
| GCF_003347305.1_ASM334730v1 |  |  |
| GCF_003347325.1_ASM334732v1 |  |  |
| GCF_003347355.1_ASM334735v1 |  |  |
| GCF_003347375.1_ASM334737v1 |  |  |
| GCF_003347385.1_ASM334738v1 |  |  |
| GCF_003347395.1_ASM334739v1 |  |  |
| GCF_003347435.1_ASM334743v1 |  |  |
| GCF_003382235.1_ASM338223v1 |  |  |
| GCF_003583525.1_ASM358352v1 |  |  |
| GCF_003583535.1_ASM358353v1 |  |  |
| GCF_003583565.1_ASM358356v1 |  |  |
| GCF_003583575.1_ASM358357v1 |  |  |
| GCF_003583615.1_ASM358361v1 |  |  |
| GCF_003583625.1_ASM358362v1 |  |  |
| GCF_003584255.1_ASM358425v1 |  |  |
| GCF_003584275.1_ASM358427v1 |  |  |
| GCF_003584315.1_ASM358431v1 |  |  |
| GCF_003584385.1_ASM358438v1 |  |  |
| GCF_003584485.1_ASM358448v1 |  |  |
| GCF_003584505.1_ASM358450v1 |  |  |
| GCF_003584515.1_ASM358451v1 |  |  |
| GCF_003584535.1_ASM358453v1 |  |  |
| GCF_003595745.1_ASM359574v1 |  |  |
| GCF_003595985.1_ASM359598v1 |  |  |
| GCF_003596015.1_ASM359601v1 |  |  |
| GCF_003596025.1_ASM359602v1 |  |  |
| GCF_003596065.1_ASM359606v1 |  |  |
| GCF_003596075.1_ASM359607v1 |  |  |
| GCF_003596135.1_ASM359613v1 |  |  |
| GCF_003596165.1_ASM359616v1 |  |  |
| GCF_003596175.1_ASM359617v1 |  |  |
| GCF_003596235.1_ASM359623v1 |  |  |
| GCF_003627405.1_ASM362740v1 |  |  |
| GCF_003627455.1_ASM362745v1 |  |  |
| GCF_003627515.1_ASM362751v1 |  |  |
| GCF_003697885.1_ASM369788v1 |  |  |
| GCF_003711855.1_ASM371185v1 |  |  |
| GCF_003711895.1_ASM371189v1 |  |  |
| GCF_003711905.1_ASM371190v1 |  |  |
| GCF_003711945.1_ASM371194v1 |  |  |
| GCF_003711985.1_ASM371198v1 |  |  |
| GCF_003843665.1_ASM384366v1 |  |  |
| GCF_003843745.1_ASM384374v1 |  |  |
| GCF_003863935.1_ASM386393v1 |  |  |
| GCF_003939395.1_ASM393939v1 |  |  |
| GCF_003939565.1_ASM393956v1 |  |  |
| GCF_003939925.1_ASM393992v1 |  |  |
| GCF_003940365.1_ASM394036v1 |  |  |
| GCF_003947815.1_ASM394781v1 |  |  |
| GCF_003947855.1_ASM394785v1 |  |  |
| GCF_003947865.1_ASM394786v1 |  |  |
| GCF_003948095.1_ASM394809v1 |  |  |
| GCF_003948125.1_ASM394812v1 |  |  |
| GCF_003948145.1_ASM394814v1 |  |  |
| GCF_003948185.1_ASM394818v1 |  |  |
| GCF_003948195.1_ASM394819v1 |  |  |
| GCF_003948225.1_ASM394822v1 |  |  |
| GCF_003948235.1_ASM394823v1 |  |  |
| GCF_003948295.1_ASM394829v1 |  |  |
| GCF_003948305.2_ASM394830v2 |  |  |
| GCF_003948345.1_ASM394834v1 |  |  |
| GCF_003948375.1_ASM394837v1 |  |  |
| GCF_003948385.1_ASM394838v1 |  |  |
| GCF_003948415.1_ASM394841v1 |  |  |
| GCF_003948425.1_ASM394842v1 |  |  |
| GCF_003948465.1_ASM394846v1 |  |  |
| GCF_003948495.1_ASM394849v1 |  |  |
| GCF_003948505.1_ASM394850v1 |  |  |
| GCF_003948545.1_ASM394854v1 |  |  |
| GCF_003948575.1_ASM394857v1 |  |  |
| GCF_003948585.1_ASM394858v1 |  |  |
| GCF_003948595.1_ASM394859v1 |  |  |
| GCF_003948605.1_ASM394860v1 |  |  |
| GCF_003948645.1_ASM394864v1 |  |  |
| GCF_003948675.1_ASM394867v1 |  |  |
| GCF_003948685.1_ASM394868v1 |  |  |
| GCF_003948725.1_ASM394872v1 |  |  |
| GCF_003948745.1_ASM394874v1 |  |  |
| GCF_003948775.1_ASM394877v1 |  |  |
| GCF_003948785.1_ASM394878v1 |  |  |
| GCF_003948795.1_ASM394879v1 |  |  |
| GCF_003948805.1_ASM394880v1 |  |  |
| GCF_003948835.1_ASM394883v1 |  |  |
| GCF_003948875.1_ASM394887v1 |  |  |
| GCF_003948885.1_ASM394888v1 |  |  |
| GCF_003948895.1_ASM394889v1 |  |  |
| GCF_003948905.1_ASM394890v1 |  |  |
| GCF_003948915.1_ASM394891v1 |  |  |
| GCF_003948975.1_ASM394897v1 |  |  |
| GCF_003948985.1_ASM394898v1 |  |  |
| GCF_003949015.1_ASM394901v1 |  |  |
| GCF_003949025.1_ASM394902v1 |  |  |
| GCF_003949075.1_ASM394907v1 |  |  |
| GCF_003949095.1_ASM394909v1 |  |  |
| GCF_003949105.1_ASM394910v1 |  |  |
| GCF_003949125.1_ASM394912v1 |  |  |
| GCF_003949175.1_ASM394917v1 |  |  |
| GCF_003949195.1_ASM394919v1 |  |  |
| GCF_003949205.1_ASM394920v1 |  |  |
| GCF_003949225.1_ASM394922v1 |  |  |
| GCF_003949235.1_ASM394923v1 |  |  |
| GCF_003949275.1_ASM394927v1 |  |  |
| GCF_003949305.1_ASM394930v1 |  |  |
| GCF_003949325.1_ASM394932v1 |  |  |
| GCF_003949335.1_ASM394933v1 |  |  |
| GCF_003949365.1_ASM394936v1 |  |  |
| GCF_003949395.1_ASM394939v1 |  |  |
| GCF_003949405.1_ASM394940v1 |  |  |
| GCF_003949425.1_ASM394942v1 |  |  |
| GCF_003949435.1_ASM394943v1 |  |  |
| GCF_003949485.1_ASM394948v1 |  |  |
| GCF_003949555.1_ASM394955v1 |  |  |
| GCF_003949575.1_ASM394957v1 |  |  |
| GCF_003949615.1_ASM394961v1 |  |  |
| GCF_003949655.1_ASM394965v1 |  |  |
| GCF_003949755.1_ASM394975v1 |  |  |
| GCF_003949765.1_ASM394976v1 |  |  |
| GCF_003949935.1_ASM394993v1 |  |  |
| GCF_003953975.1_ASM395397v1 |  |  |
| GCF_003953985.1_ASM395398v1 |  |  |
| GCF_003954005.1_ASM395400v1 |  |  |
| GCF_003954085.1_ASM395408v1 |  |  |
| GCF_003954095.1_ASM395409v1 |  |  |
| GCF_003954105.1_ASM395410v1 |  |  |
| GCF_003954115.1_ASM395411v1 |  |  |
| GCF_003954175.1_ASM395417v1 |  |  |
| GCF_003954215.1_ASM395421v1 |  |  |
| GCF_003954235.1_ASM395423v1 |  |  |
| GCF_003954275.1_ASM395427v1 |  |  |
| GCF_003954315.1_ASM395431v1 |  |  |
| GCF_003954365.1_ASM395436v1 |  |  |
| GCF_003954415.1_ASM395441v1 |  |  |
| GCF_003954595.1_ASM395459v1 |  |  |
| GCF_003954795.1_ASM395479v1 |  |  |
| GCF_003954845.1_ASM395484v1 |  |  |
| GCF_003954905.1_ASM395490v1 |  |  |
| GCF_003955235.1_ASM395523v1 |  |  |
| GCF_003955305.1_ASM395530v1 |  |  |
| GCF_003955355.1_ASM395535v1 |  |  |
| GCF_003955385.1_ASM395538v1 |  |  |
| GCF_003955435.1_ASM395543v1 |  |  |
| GCF_003955485.1_ASM395548v1 |  |  |
| GCF_003965825.1_ASM396582v1 |  |  |
| GCF_003966015.1_ASM396601v1 |  |  |
| GCF_003970215.1_ASM397021v1 |  |  |
| GCF_003976955.1_ASM397695v1 |  |  |
| GCF_003976985.1_ASM397698v1 |  |  |
| GCF_003977015.1_ASM397701v1 |  |  |
| GCF_003977045.1_ASM397704v1 |  |  |
| GCF_003978795.1_ASM397879v1 |  |  |
| GCF_003979005.1_ASM397900v1 |  |  |
| GCF_003984795.1_ASM398479v1 |  |  |
| GCF_003991295.1_ASM399129v1 |  |  |
| GCF_003994455.1_ASM399445v1 |  |  |
| GCF_004101965.1_ASM410196v1 |  |  |
| GCF_004101985.1_ASM410198v1 |  |  |
| GCF_004152685.1_ASM415268v1 |  |  |
| GCF_004152715.1_ASM415271v1 |  |  |
| GCF_004152785.1_ASM415278v1 |  |  |
| GCF_004152945.1_ASM415294v1 |  |  |
| GCF_004153295.1_ASM415329v1 |  |  |
| GCF_004168615.1_ASM416861v1 |  |  |
| GCF_004208995.1_ASM420899v1 |  |  |
| GCF_004209005.1_ASM420900v1 |  |  |
| GCF_004209025.1_ASM420902v1 |  |  |
| GCF_004209035.1_ASM420903v1 |  |  |
| GCF_004209075.1_ASM420907v1 |  |  |
| GCF_004209195.1_ASM420919v1 |  |  |
| GCF_004209205.1_ASM420920v1 |  |  |
| GCF_004209225.1_ASM420922v1 |  |  |
| GCF_004209675.1_ASM420967v1 |  |  |
| GCF_004209715.1_ASM420971v1 |  |  |
| GCF_004282845.1_ASM428284v1 |  |  |
| GCF_004296275.1_ASM429627v1 |  |  |
| GCF_004299615.1_ASM429961v1 |  |  |
| GCF_004321575.1_ASM432157v1 |  |  |
| GCF_004321595.1_ASM432159v1 |  |  |
| GCF_004347325.1_ASM434732v1 |  |  |
| GCF_004357075.1_ASM435707v1 |  |  |
| GCF_004357095.1_ASM435709v1 |  |  |
| GCF_004357175.1_ASM435717v1 |  |  |
| GCF_004357185.1_ASM435718v1 |  |  |
| GCF_004357205.1_ASM435720v1 |  |  |
| GCF_004357225.1_ASM435722v1 |  |  |
| GCF_004357275.1_ASM435727v1 |  |  |
| GCF_004357285.1_ASM435728v1 |  |  |
| GCF_004357295.1_ASM435729v1 |  |  |
| GCF_004359905.1_ASM435990v1 |  |  |
| GCF_004377785.1_ASM437778v1 |  |  |
| GCF_004378285.1_ASM437828v1 |  |  |
| GCF_004378295.1_ASM437829v1 |  |  |
| GCF_004378395.1_ASM437839v1 |  |  |
| GCF_004378435.1_ASM437843v1 |  |  |
| GCF_004378505.1_ASM437850v1 |  |  |
| GCF_004378525.1_ASM437852v1 |  |  |
| GCF_004378535.1_ASM437853v1 |  |  |
| GCF_004378545.1_ASM437854v1 |  |  |
| GCF_004378625.1_ASM437862v1 |  |  |
| GCF_004790725.1_ASM479072v1 |  |  |
| GCF_004799205.1_ASM479920v1 |  |  |
| GCF_004799225.1_ASM479922v1 |  |  |
| GCF_004912235.1_ASM491223v1 |  |  |
| GCF_004912245.1_ASM491224v1 |  |  |
| GCF_005048905.1_ASM504890v1 |  |  |
| GCF_005298065.1_ASM529806v1 |  |  |
| GCF_005298085.1_ASM529808v1 |  |  |
| GCF_005819005.1_ASM581900v1 |  |  |
| GCF_005819035.1_ASM581903v1 |  |  |
| GCF_005819065.1_ASM581906v1 |  |  |
| GCF_005819085.1_ASM581908v1 |  |  |
| GCF_005819095.1_ASM581909v1 |  |  |
| GCF_005819115.1_ASM581911v1 |  |  |
| GCF_005819135.1_ASM581913v1 |  |  |
| GCF_005819165.1_ASM581916v1 |  |  |
| GCF_005819175.1_ASM581917v1 |  |  |
| GCF_005819205.1_ASM581920v1 |  |  |
| GCF_005819215.1_ASM581921v1 |  |  |
| GCF_005819225.1_ASM581922v1 |  |  |
| GCF_005819265.1_ASM581926v1 |  |  |
| GCF_005819275.1_ASM581927v1 |  |  |
| GCF_005819305.1_ASM581930v1 |  |  |
| GCF_005819315.1_ASM581931v1 |  |  |
| GCF_005819325.1_ASM581932v1 |  |  |
| GCF_005819365.1_ASM581936v1 |  |  |
| GCF_005819375.1_ASM581937v1 |  |  |
| GCF_005819405.1_ASM581940v1 |  |  |
| GCF_005819415.1_ASM581941v1 |  |  |
| GCF_005819425.1_ASM581942v1 |  |  |
| GCF_005819445.1_ASM581944v1 |  |  |
| GCF_005819475.1_ASM581947v1 |  |  |
| GCF_005819505.1_ASM581950v1 |  |  |
| GCF_005819515.1_ASM581951v1 |  |  |
| GCF_005819535.1_ASM581953v1 |  |  |
| GCF_005819545.1_ASM581954v1 |  |  |
| GCF_005819585.1_ASM581958v1 |  |  |
| GCF_005819595.1_ASM581959v1 |  |  |
| GCF_005819625.1_ASM581962v1 |  |  |
| GCF_005819635.1_ASM581963v1 |  |  |
| GCF_005819645.1_ASM581964v1 |  |  |
| GCF_005819685.1_ASM581968v1 |  |  |
| GCF_005819705.1_ASM581970v1 |  |  |
| GCF_005819725.1_ASM581972v1 |  |  |
| GCF_005819735.1_ASM581973v1 |  |  |
| GCF_005819755.1_ASM581975v1 |  |  |
| GCF_005819765.1_ASM581976v1 |  |  |
| GCF_005863285.1_ASM586328v1 |  |  |
| GCF_005863305.1_ASM586330v1 |  |  |
| GCF_005863345.1_ASM586334v1 |  |  |
| GCF_005863355.1_ASM586335v1 |  |  |
| GCF_005863385.1_ASM586338v1 |  |  |
| GCF_005863395.1_ASM586339v1 |  |  |
| GCF_005863405.1_ASM586340v1 |  |  |
| GCF_005863415.1_ASM586341v1 |  |  |
| GCF_005863455.1_ASM586345v1 |  |  |
| GCF_005863535.1_ASM586353v1 |  |  |
| GCF_006365355.1_ASM636535v1 |  |  |
| GCF_006365365.1_ASM636536v1 |  |  |
| GCF_006365375.1_ASM636537v1 |  |  |
| GCF_006365385.1_ASM636538v1 |  |  |
| GCF_006365405.1_ASM636540v1 |  |  |
| GCF_006365455.1_ASM636545v1 |  |  |
| GCF_006365465.1_ASM636546v1 |  |  |
| GCF_006365495.1_ASM636549v1 |  |  |
| GCF_006365515.1_ASM636551v1 |  |  |
| GCF_006365525.1_ASM636552v1 |  |  |
| GCF_006365555.1_ASM636555v1 |  |  |
| GCF_006365565.1_ASM636556v1 |  |  |
| GCF_006365575.1_ASM636557v1 |  |  |
| GCF_006365585.1_ASM636558v1 |  |  |
| GCF_006365635.1_ASM636563v1 |  |  |
| GCF_006365655.1_ASM636565v1 |  |  |
| GCF_006365665.1_ASM636566v1 |  |  |
| GCF_006365675.1_ASM636567v1 |  |  |
| GCF_006365685.1_ASM636568v1 |  |  |
| GCF_006365735.1_ASM636573v1 |  |  |
| GCF_006365745.1_ASM636574v1 |  |  |
| GCF_006365775.1_ASM636577v1 |  |  |
| GCF_006365795.1_ASM636579v1 |  |  |
| GCF_006365845.1_ASM636584v1 |  |  |
| GCF_006365865.1_ASM636586v1 |  |  |
| GCF_006365885.1_ASM636588v1 |  |  |
| GCF_006365895.1_ASM636589v1 |  |  |
| GCF_006365935.1_ASM636593v1 |  |  |
| GCF_006365945.1_ASM636594v1 |  |  |
| GCF_006365955.1_ASM636595v1 |  |  |
| GCF_006365965.1_ASM636596v1 |  |  |
| GCF_006365975.1_ASM636597v1 |  |  |
| GCF_006366035.1_ASM636603v1 |  |  |
| GCF_006366055.1_ASM636605v1 |  |  |
| GCF_006366065.1_ASM636606v1 |  |  |
| GCF_006366115.1_ASM636611v1 |  |  |
| GCF_006366135.1_ASM636613v1 |  |  |
| GCF_006366155.1_ASM636615v1 |  |  |
| GCF_006366195.1_ASM636619v1 |  |  |
| GCF_006366215.1_ASM636621v1 |  |  |
| GCF_006366235.1_ASM636623v1 |  |  |
| GCF_006366245.1_ASM636624v1 |  |  |
| GCF_006366275.1_ASM636627v1 |  |  |
| GCF_006366285.1_ASM636628v1 |  |  |
| GCF_006366315.1_ASM636631v1 |  |  |
| GCF_006366335.1_ASM636633v1 |  |  |
| GCF_006366355.1_ASM636635v1 |  |  |
| GCF_006366365.1_ASM636636v1 |  |  |
| GCF_006366385.1_ASM636638v1 |  |  |
| GCF_006366405.1_ASM636640v1 |  |  |
| GCF_006366435.1_ASM636643v1 |  |  |
| GCF_006366445.1_ASM636644v1 |  |  |
| GCF_006366465.1_ASM636646v1 |  |  |
| GCF_006366475.1_ASM636647v1 |  |  |
| GCF_006366535.1_ASM636653v1 |  |  |
| GCF_006366545.1_ASM636654v1 |  |  |
| GCF_006366575.1_ASM636657v1 |  |  |
| GCF_006366585.1_ASM636658v1 |  |  |
| GCF_006366635.1_ASM636663v1 |  |  |
| GCF_006366645.1_ASM636664v1 |  |  |
| GCF_006366665.1_ASM636666v1 |  |  |
| GCF_006366685.1_ASM636668v1 |  |  |
| GCF_006366715.1_ASM636671v1 |  |  |
| GCF_006366735.1_ASM636673v1 |  |  |
| GCF_006366745.1_ASM636674v1 |  |  |
| GCF_006366755.1_ASM636675v1 |  |  |
| GCF_006366785.1_ASM636678v1 |  |  |
| GCF_006366805.1_ASM636680v1 |  |  |
| GCF_006366825.1_ASM636682v1 |  |  |
| GCF_006366845.1_ASM636684v1 |  |  |
| GCF_006366875.1_ASM636687v1 |  |  |
| GCF_006366895.1_ASM636689v1 |  |  |
| GCF_006366905.1_ASM636690v1 |  |  |
| GCF_006366925.1_ASM636692v1 |  |  |
| GCF_006366935.1_ASM636693v1 |  |  |
| GCF_006366975.1_ASM636697v1 |  |  |
| GCF_006366985.1_ASM636698v1 |  |  |
| GCF_006367015.1_ASM636701v1 |  |  |
| GCF_006367025.1_ASM636702v1 |  |  |
| GCF_006367045.1_ASM636704v1 |  |  |
| GCF_006367055.1_ASM636705v1 |  |  |
| GCF_006367085.1_ASM636708v1 |  |  |
| GCF_006367115.1_ASM636711v1 |  |  |
| GCF_006367125.1_ASM636712v1 |  |  |
| GCF_006367135.1_ASM636713v1 |  |  |
| GCF_006367175.1_ASM636717v1 |  |  |
| GCF_006367195.1_ASM636719v1 |  |  |
| GCF_006367205.1_ASM636720v1 |  |  |
| GCF_006367225.1_ASM636722v1 |  |  |
| GCF_006367245.1_ASM636724v1 |  |  |
| GCF_006367275.1_ASM636727v1 |  |  |
| GCF_006367285.1_ASM636728v1 |  |  |
| GCF_006367295.1_ASM636729v1 |  |  |
| GCF_006367335.1_ASM636733v1 |  |  |
| GCF_006367345.1_ASM636734v1 |  |  |
| GCF_006367375.1_ASM636737v1 |  |  |
| GCF_006367395.1_ASM636739v1 |  |  |
| GCF_006367405.1_ASM636740v1 |  |  |
| GCF_006367435.1_ASM636743v1 |  |  |
| GCF_006367475.1_ASM636747v1 |  |  |
| GCF_006367485.1_ASM636748v1 |  |  |
| GCF_006367505.1_ASM636750v1 |  |  |
| GCF_006367515.1_ASM636751v1 |  |  |
| GCF_006367535.1_ASM636753v1 |  |  |
| GCF_006367595.1_ASM636759v1 |  |  |
| GCF_006367605.1_ASM636760v1 |  |  |
| GCF_006367615.1_ASM636761v1 |  |  |
| GCF_006367625.1_ASM636762v1 |  |  |
| GCF_006367675.1_ASM636767v1 |  |  |
| GCF_006367685.1_ASM636768v1 |  |  |
| GCF_006367695.1_ASM636769v1 |  |  |
| GCF_006367735.1_ASM636773v1 |  |  |
| GCF_006367745.1_ASM636774v1 |  |  |
| GCF_006367765.1_ASM636776v1 |  |  |
| GCF_006367795.1_ASM636779v1 |  |  |
| GCF_006367805.1_ASM636780v1 |  |  |
| GCF_006367835.1_ASM636783v1 |  |  |
| GCF_006367845.1_ASM636784v1 |  |  |
| GCF_006367855.1_ASM636785v1 |  |  |
| GCF_006367895.1_ASM636789v1 |  |  |
| GCF_006367905.1_ASM636790v1 |  |  |
| GCF_006367915.1_ASM636791v1 |  |  |
| GCF_006367945.1_ASM636794v1 |  |  |
| GCF_006367965.1_ASM636796v1 |  |  |
| GCF_006367995.1_ASM636799v1 |  |  |
| GCF_006368005.1_ASM636800v1 |  |  |
| GCF_006368025.1_ASM636802v1 |  |  |
| GCF_006368045.1_ASM636804v1 |  |  |
| GCF_006368055.1_ASM636805v1 |  |  |
| GCF_006368095.1_ASM636809v1 |  |  |
| GCF_006368105.1_ASM636810v1 |  |  |
| GCF_006368125.1_ASM636812v1 |  |  |
| GCF_006368155.1_ASM636815v1 |  |  |
| GCF_006368165.1_ASM636816v1 |  |  |
| GCF_006369695.1_ASM636969v1 |  |  |
| GCF_006491855.1_ASM649185v1 |  |  |
| GCF_006491865.1_ASM649186v1 |  |  |
| GCF_006491875.1_ASM649187v1 |  |  |
| GCF_006491885.1_ASM649188v1 |  |  |
| GCF_006491955.1_ASM649195v1 |  |  |
| GCF_006491975.1_ASM649197v1 |  |  |
| GCF_006492055.1_ASM649205v1 |  |  |
| GCF_006492075.1_ASM649207v1 |  |  |
| GCF_006492085.1_ASM649208v1 |  |  |
| GCF_006492125.1_ASM649212v1 |  |  |
| GCF_006492155.1_ASM649215v1 |  |  |
| GCF_006492165.1_ASM649216v1 |  |  |
| GCF_006492175.1_ASM649217v1 |  |  |
| GCF_006492215.1_ASM649221v1 |  |  |
| GCF_006492245.1_ASM649224v1 |  |  |
| GCF_006492265.1_ASM649226v1 |  |  |
| GCF_006492295.1_ASM649229v1 |  |  |
| GCF_006492305.1_ASM649230v1 |  |  |
| GCF_006492315.1_ASM649231v1 |  |  |
| GCF_006492335.1_ASM649233v1 |  |  |
| GCF_006492375.1_ASM649237v1 |  |  |
| GCF_006492385.1_ASM649238v1 |  |  |
| GCF_006492395.1_ASM649239v1 |  |  |
| GCF_006492405.1_ASM649240v1 |  |  |
| GCF_006492425.1_ASM649242v1 |  |  |
| GCF_006492475.1_ASM649247v1 |  |  |
| GCF_006492495.1_ASM649249v1 |  |  |
| GCF_006492505.1_ASM649250v1 |  |  |
| GCF_006492515.1_ASM649251v1 |  |  |
| GCF_006492575.1_ASM649257v1 |  |  |
| GCF_006492585.1_ASM649258v1 |  |  |
| GCF_006492615.1_ASM649261v1 |  |  |
| GCF_006492625.1_ASM649262v1 |  |  |
| GCF_006492635.1_ASM649263v1 |  |  |
| GCF_006492675.1_ASM649267v1 |  |  |
| GCF_006492685.1_ASM649268v1 |  |  |
| GCF_006492705.1_ASM649270v1 |  |  |
| GCF_006492715.1_ASM649271v1 |  |  |
| GCF_006492735.1_ASM649273v1 |  |  |
| GCF_006492775.1_ASM649277v1 |  |  |
| GCF_006492785.1_ASM649278v1 |  |  |
| GCF_006492795.1_ASM649279v1 |  |  |
| GCF_006492805.1_ASM649280v1 |  |  |
| GCF_006492855.1_ASM649285v1 |  |  |
| GCF_006492865.1_ASM649286v1 |  |  |
| GCF_006492885.1_ASM649288v1 |  |  |
| GCF_006492905.1_ASM649290v1 |  |  |
| GCF_006492925.1_ASM649292v1 |  |  |
| GCF_006492945.1_ASM649294v1 |  |  |
| GCF_006492965.1_ASM649296v1 |  |  |
| GCF_006492985.1_ASM649298v1 |  |  |
| GCF_006493005.1_ASM649300v1 |  |  |
| GCF_006493035.1_ASM649303v1 |  |  |
| GCF_006493045.1_ASM649304v1 |  |  |
| GCF_006493645.1_ASM649364v1 |  |  |
| GCF_006493655.1_ASM649365v1 |  |  |
| GCF_006493685.1_ASM649368v1 |  |  |
| GCF_006493705.1_ASM649370v1 |  |  |
| GCF_006493735.1_ASM649373v1 |  |  |
| GCF_006493745.1_ASM649374v1 |  |  |
| GCF_006493755.1_ASM649375v1 |  |  |
| GCF_006493765.1_ASM649376v1 |  |  |
| GCF_006493815.1_ASM649381v1 |  |  |
| GCF_006493835.1_ASM649383v1 |  |  |
| GCF_006493845.1_ASM649384v1 |  |  |
| GCF_006493855.1_ASM649385v1 |  |  |
| GCF_006493875.1_ASM649387v1 |  |  |
| GCF_006493915.1_ASM649391v1 |  |  |
| GCF_006493925.1_ASM649392v1 |  |  |
| GCF_006493945.1_ASM649394v1 |  |  |
| GCF_006493955.1_ASM649395v1 |  |  |
| GCF_006493975.1_ASM649397v1 |  |  |
| GCF_006494005.1_ASM649400v1 |  |  |
| GCF_006494015.1_ASM649401v1 |  |  |
| GCF_006494055.1_ASM649405v1 |  |  |
| GCF_006494065.1_ASM649406v1 |  |  |
| GCF_006494075.1_ASM649407v1 |  |  |
| GCF_006494085.1_ASM649408v1 |  |  |
| GCF_006494115.1_ASM649411v1 |  |  |
| GCF_006494175.1_ASM649417v1 |  |  |
| GCF_006494225.1_ASM649422v1 |  |  |
| GCF_006494255.1_ASM649425v1 |  |  |
| GCF_006494265.1_ASM649426v1 |  |  |
| GCF_006494285.1_ASM649428v1 |  |  |
| GCF_006494305.1_ASM649430v1 |  |  |
| GCF_006494565.1_ASM649456v1 |  |  |
| GCF_006494575.1_ASM649457v1 |  |  |
| GCF_006494585.1_ASM649458v1 |  |  |
| GCF_006494615.1_ASM649461v1 |  |  |
| GCF_006494665.1_ASM649466v1 |  |  |
| GCF_900010325.1_BAL_242_assembly |  |  |
| GCF_900010465.1_BAL_062_Assembly |  |  |
| GCF_900011275.1_BAL_255_assembly |  |  |
| GCF_900011285.1_BAL_266_Assembly |  |  |
| GCF_900011295.1_ATCC19606_assembly |  |  |
| GCF_900011305.1_BAL_062_assembly |  |  |
| GCF_900117405.1_ABE12_M |  |  |
| GCF_900119395.1_ASM90011939v1 |  |  |
| GCF_900157395.1_H31506 |  |  |
| GCF_900157405.1_H31499 |  |  |
| GCF_900161875.1_68SM01 |  |  |
| GCF_900161885.1_103SM |  |  |
| GCF_900161895.1_14336 |  |  |
| GCF_900161905.1_2MG |  |  |
| GCF_900161915.1_5MO |  |  |
| GCF_900161925.1_96SM |  |  |
| GCF_900161935.1_MGTN |  |  |
| GCF_900161945.1_61SM01 |  |  |
| GCF_900161955.1_20C15 |  |  |
| GCF_900161965.1_74SM01 |  |  |
| GCF_900161975.1_65SM01 |  |  |
| GCF_900161985.1_25C30 |  |  |
| GCF_900161995.1_2RED09 |  |  |
| GCF_900162005.1_MONUR |  |  |
| GCF_900162015.1_72SM01 |  |  |
| GCF_900174435.1_Acinetobacter_baumannii |  |  |
| GCF_900175175.1_AE27M |  |  |
| GCF_900175185.1_AE21M |  |  |
| GCF_900176685.1_AC40M |  |  |
| GCF_900176715.1_AC15M |  |  |
| GCF_900243925.1_K50 |  |  |
| GCF_900406645.1_De_novo_assembly_of_KCRI_isolate_RDK02_116 |  |  |
| GCF_900406655.1_De_novo_assembly_of_KCRI_isolate_RDK36_33 |  |  |
| GCF_900406665.1_De_novo_assembly_of_KCRI_isolate_RDK03_186 |  |  |
| GCF_900406675.1_De_novo_assembly_of_KCRI_isolate_RDK01_164C |  |  |
| GCF_900406685.1_De_novo_assembly_of_KCRI_isolate_RDK36_28 |  |  |
| GCF_900406695.1_De_novo_assembly_of_KCRI_isolate_R0004_123C |  |  |
| GCF_900406705.1_De_novo_assembly_of_KCRI_isolate_RDK01_309C |  |  |
| GCF_900406725.1_De_novo_assembly_of_KCRI_isolate_RDK06_432B |  |  |
| GCF_900406735.1_De_novo_assembly_of_KCRI_isolate_RDK37_43 |  |  |
| GCF_900406775.1_De_novo_assembly_of_KCRI_isolate_RDK39_49 |  |  |
| GCF_900444715.1_53990_B01 |  |  |
| GCF_900444725.1_34576_B02 |  |  |
| GCF_900444735.1_54669_D01 |  |  |
| GCF_900444745.1_34555_A01 |  |  |
| GCF_900444755.1_59123_D01 |  |  |
| GCF_900444765.1_34555_E01 |  |  |
| GCF_900444775.1_34555_B01 |  |  |
| GCF_900444785.1_34555_C01 |  |  |
| GCF_900444795.1_34576_A01 |  |  |
| GCF_900476545.1_7468_2_58 |  |  |
| GCF_900476555.1_7468_2_57 |  |  |
| GCF_900476595.1_7468_2_49 |  |  |
| GCF_900476615.1_7468_2_66 |  |  |
| GCF_900476635.1_7468_2_63 |  |  |
| GCF_900476645.1_7468_2_65 |  |  |
| GCF_900476655.1_7468_2_61 |  |  |
| GCF_900476725.1_7468_2_69 |  |  |
| GCF_900476745.1_7468_2_67 |  |  |
| GCF_900476765.1_7468_2_62 |  |  |
| GCF_900490075.1_7468_2_64 |  |  |
| GCF_900494775.1_24276_2_163 |  |  |
| GCF_900494815.1_24276_2_168 |  |  |
| GCF_900494885.1_24276_2_184 |  |  |
| GCF_900495005.1_24276_2_170 |  |  |
| GCF_900495015.1_24276_2_185 |  |  |
| GCF_900495405.1_24276_2_217 |  |  |
| GCF_900495415.1_24276_2_236 |  |  |
| GCF_900495545.1_24276_2_218 |  |  |
| GCF_900495605.1_24276_2_244 |  |  |
| GCF_900496075.1_24276_2_291 |  |  |
| GCF_900496415.1_24276_3_19 |  |  |
| GCF_900496465.1_24276_3_24 |  |  |
| GCF_900496545.1_24276_3_32 |  |  |
| GCF_900496615.1_24276_3_31 |  |  |
| GCF_900519195.1_Acinetobacter_baumannii_12918 |  |  |
| GCF_900608095.1_abaum007 |  |  |
| GCF_901669785.1_aba147518_assembly |  |  |
| GCF_901669935.1_aba196618_assembly |  |  |
| GCF_901669965.1_aba130618_assembly |  |  |
| GCF_901669975.1_aba44921_assembly |  |  |
|  |  |  |
|  |  |  |
|  |  |  |
|  |  |  |
|  |  |  |
|  |  |  |
|  |  |  |
|  |  |  |
|  |  |  |
|  |  |  |
|  |  |  |
|  |  |  |
|  |  |  |
|  |  |  |
|  |  |  |
|  |  |  |
|  |  |  |
