# Supplementary material for: Crucial Role of the Accessory Genome in the Evolutionary Trajectory of Acinetobacter baumannii Global Clone 1
Source: Front Microbiol. 2020 Mar 18;11:342. doi: 10.3389/fmicb.2020.00342 (PMC7093585; doi:10.3389/fmicb.2020.00342)
Supplement: Supplementary file 4 [file Table_2.docx]

**Supplementary Table 2. Unique genes found in A144, A155 and AYE genomes compared to GC1 Group 1, respectively.** Columns 1, 2 and 3 detail unique genes found in A144, A155 and AYE genomes when compared each one to the other GC1 Group 1, respectively**.** GC1 Group 1 in column 4 refers to GC1 Group 1 unique genes compared to Outgroup Group 3.

| **A144** | A155 | **AYE** | **GC1 Group 1** |
| --- | --- | --- | --- |
| 3720_hypothetical_  protein, ATP-binding protein | 7722_phage tail tape measure protein, partial | 22234_hypothetical_protein | 245_hypothetical_protein |
| 4302_hypothetical_  protein, DUF551 domain-containing protein | 7723_hypothetical_protein | 22235_hypothetical_protein | 318_pilin |
| 4304_hypothetical_  protein | 8110_ubiA_1 | 22364_hypothetical_protein | 377_DUF262 domain-containing protein |
| 4305_hypothetical_  protein, prophage LambdaSa2, HNH endonuclease family protein | 8111_apaH | 22661_hypothetical_protein | 378_hypothetical_protein |
| 4306_hypothetical_  protein | 8120_Cyclic di-GMP phosphodiesterase gmr | 22665_hypothetical_protein | 921_hypothetical_protein |
| 4308_hypothetical_  protein | 8354_hypothetical_protein | 22670_hypothetical_protein | 924_hypothetical_protein |
| 4309_hypothetical_  protein | 8356_hypothetical_protein | 22806_hypothetical_protein | 925_hypothetical_protein |
| 4310_hypothetical_  protein | 8359_hypothetical_protein | 22901_conserved_hypothetic | 1069_cas1f |
| 4318_hypothetical_  protein | 8360_hypothetical_protein | 22950_hypothetical_protein | 1070_cas3f |
| 4320_hypothetical_  protein | 8968_hypothetical_protein | 23212_conserved_  hypothetical protein | 1071_csy1 |
| 4359_hypothetical_  protein | 9756_hypothetical_protein | 23267_transposase_  of_IS10A | 1072_csy2 |
| 4437_hypothetical_  protein | 9762_hypothetical_protein | 23302_hypothetical_protein | 1073_csy3 |
| 4466_hypothetical_  protein | 10179_hypothetical_protein | 23304_hypothetical_protein | 1074_cas6f |
| 4820_qseB_2 | 10184_hypothetical_protein | 23307_hypothetical_protein | 1481_heme-binding_protein |
| 4821_qseB_3 | 10634_hypothetical_protein | 23308_hypothetical_protein | 1484_lipocalin_  family_protein |
| 5076_hypothetical_  protein | 11030_hypothetical_protein | 23310_hypothetical_protein | 1485_acyl-CoA_desaturase |
| 5116_hypothetical_  protein |  | 23312_hypothetical_protein | 1487_DUF1365_  domain-containing protein |
| 5571_cyaA |  | 23314_hypothetical_protein | 1488_class_I_SAM-dependent methyltransferase |
| 5573_hypothetical_  protein, phage tail protein |  | 23316_hypothetical_protein | 1489_class_I_SAM-dependent methyltransferase |
| 5590_hypothetical_  protein |  | 23317_hypothetical_protein | 1490_HAMP_domain-containing protein |
| 6323_hypothetical_  protein |  | 23327_hypothetical_protein | 1491_response_regulator |
| 6325_hypothetical_  protein |  | 23336_hypothetical_protein | 1492_hypothetical_protein |
| 6352_hypothetical_  protein |  | 23341_hypothetical_protein | 1493_nuclear_transport_  family |
| 6381_hypothetical_  protein |  | 23343_hypothetical_protein | 1616_AAA_family_ATPase |
| 6382_hypothetical_  protein |  | 23346_hypothetical_protein | 1617_hypothetical_protein |
| 6384_hypothetical_  protein |  | 23670_conserved_  hypothetical protein | 1861_SDR_family_  oxidoreductase |
| 6385_hypothetical_  protein |  | 23863_hypothetical_protein | 1862_acetoacetate_  decarboxylase |
| 6403_hypothetical_  protein |  | 23870_hypothetical_protein | 1863_metal-dependent_hydrolase |
| 6413_hypothetical_  protein |  | 23875_hypothetical_protein | 1924_alpha-beta_hydrolase |
| 6427_hypothetical_  protein |  | 23877_hypothetical_protein | 2440_hydroxymethylglutaryl-coa reductase |
| 6443_hypothetical_  protein |  | 23898_hypothetical_protein | 2441_CoA_transferase |
| 6453_hypothetical_  protein |  | 23904_hypothetical_protein | 2442_LysR_family_transcription protein |
| 6878_copA_2, copper resistance system multicopper oxidase |  | 23905_hypothetical_protein | 2443_hypothetical_  protein |
| 6991_hypothetical_  protein |  | 23916_hypothetical_protein | 2447_hypothetical_  protein |
| 7112_hypothetical_  protein |  | 24004_conserved_  hypothetical protein | 2524_hypothetical_  protein |
| 7633_zitB_3, zinc transporter ZitB |  | 24267_hypothetical_protein | 2525_hypothetical_  protein |
| 7634_czcD_3, cobalt transporter |  | 24409_putative_  Biotin_carboxyl carrier protein | 2741_RHS_repeat_  protein |
|  |  | 24565_conserved  _hypothetical protein | 3167_hypothetical_  protein |
|  |  | 24569_conserved_  hypothetical protein | 3789_DUF4844__  domain-containing protein |
|  |  | 24604_hypothetical_protein |  |
|  |  | 24692_hypothetical_protein |  |
|  |  | 24695_hypothetical_protein |  |
|  |  | 24703_ conserved  hypothetical protein |  |
|  |  | 24704_ conserved  hypothetical protein |  |
|  |  | 24710_hypothetical_protein |  |
|  |  | 24712_hypothetical_protein |  |
|  |  | 24847_hypothetical_protein |  |
|  |  | 25090_ conserved  hypothetical protein |  |
|  |  | 25467_hypothetical_protein |  |
|  |  | 25469_hypothetical_protein |  |
|  |  | 25470_ conserved  hypothetical protein |  |
|  |  | 25487_hypothetical_protein |  |
|  |  | 25488_hypothetical_protein |  |
|  |  | 25489_ conserved  hypothetical protein |  |
|  |  | 25490_hypothetical_protein |  |
|  |  | 25491_ conserved  hypothetical protein |  |
|  |  | 25495_putative_Relaxase-he |  |
|  |  | 25501_ conserved  hypothetical protein |  |
|  |  | 25505_dfrA10 |  |
|  |  | 25506_putative_  transposase |  |
|  |  | 25510_oxa-10 |  |
|  |  | 25511_cmlA |  |
|  |  | 25512_arr-2 |  |
|  |  | 25516_GroEL-integrase_fusion |  |
|  |  | 25517_putative_ATP-dependendent DNA helicase |  |
|  |  | 25518_N-acetyltransferase AAC(6')-Ian |  |
|  |  | 25520_putative_  transcriptional LysR family regulator |  |
|  |  | 25525_dhfrI |  |
|  |  | 25527_strB |  |
|  |  | 25528_aphE |  |
|  |  | 25571_conserved hypothetical protein |  |
|  |  | 25652_hypothetical_protein |  |
|  |  | 25656_hypothetical_protein |  |
|  |  | 25660_conserved hypothetical protein |  |
