# Supplementary material for: Crucial Role of the Accessory Genome in the Evolutionary Trajectory of Acinetobacter baumannii Global Clone 1
Source: Front Microbiol. 2020 Mar 18;11:342. doi: 10.3389/fmicb.2020.00342 (PMC7093585; doi:10.3389/fmicb.2020.00342)
Supplement: Supplementary file 6 [file Table_4.docx]

**Supplementary Table 4.** **Characteristics of plasmids found in GC1 Group 1 and Outgroup Group 3 genomes.** The pA144 and pA155 are DNA sequences excluded from the chromosomal pseudomolecules of A144 and A155 genomes, respectively. A Rep_3 predicted protein with 100% query cover and 100% identity with WP_005133531.1 was identified in each putative plasmid.

| **Strain** | **Plasmids** | **Accession** | **Reference** | **Predicted Rep_3 Proteins** | **Resistance in** | **Tn/IS in plasmids** |
| --- | --- | --- | --- | --- | --- | --- |
|  |  | **Number** |  |  | **plasmids** |  |
| **A144** | pA144 (28528 bp) |  | This study | RepB (Rep_3 Superfamily) WP_005133531.1 | *aac(3)-IIa*, *bla*_TEM-1B_ | Tn*3* |
| **A155** | pA155 (20062 pb) |  | This study | RepB (Rep_3 Superfamily) WP_005133531.1 | *aac(3)-IIa*, *bla*_TEM-1B_ | Tn*3* |
| **A1** | pA1-1 (8731 bp) | NZ_CP010782.1 | Holt et al., 2015 | RepAci1 (Rep_3 Superfamily) (ALJ89812.1) | -^1^ | - |
| **AB307-0294** | - | ND^2^ | Adams et al., 2008 | ND | ND | ND |
| **AYE** | p1ABAYE (5644 bp) | CU459137.1 | Vallenet et al., 2008 | Rep_3-like^3^  WP_065996348.1 | - | - |
|  | p2ABAYE (9661 bp) | CU459138.1 | Vallenet et al., 2008 | RepAci1 (Rep_3 Superfamily) (ALJ89812.1) | - | - |
|  | p3ABAYE (94413 bp) | CU459140.1 | Vallenet et al., 2008 | RepA1 (Rep_3 Superfamily)  (ALJ89824.1) | - | IS*Aba5* |
|  | p4ABAYE (2726 bp) | CU459139.1 | Vallenet et al., 2008 | - | - | - |
| **AB0057** | pAB0057 (8729 bp) | NC_011585.2 | Adams et al., 2008 | RepAci1 (Rep_3 Superfamily) (ALJ89812.1) | - | - |
| **AB5075-UW** | p1AB5075 (83610 bp) | NZ_CP008707.1 | Gallagher et al., 2015 | PriCT_1 (ANC38839.1) | *strA,strB,aadA2,aadB,aphA6,*  *bla_GES-14,_aac(6')Ib,cmlA* | IS*Aba125* |
|  | p2AB5075 (8731 bp) | NZ_CP008708.1 | Gallagher et al., 2015 | RepAci1 (Rep_3 Superfamily) (ALJ89812.1) | - | - |
|  | p3AB5075 (1967 bp) | NZ_CP008709.1 | Gallagher et al., 2015 | - | - | - |
| **D36** | pD36-1 (4754 bp) | NZ_CP012953.1 | Hamidian et al., 2015, 2018 | - | - | - |
|  | pD36-2 (6078 bp) | NZ_CP012954.1 | Hamidian et al., 2015, 2018 | - | *aadB* | - |
|  | pD36-3 (9276 bp) | NZ_CP012955.1 | Hamidian et al., 2015, 2018 | RepAci1 (Rep_3 Superfamily) (ALJ89812.1) | - | - |
|  | pD36-4 (47457 bp) | NZ_CP012956.1 | Hamidian et al., 2015, 2018 | RepA1 (Rep_3 Superfamily) (ALJ89824.1) | *sul2, aphA1, mer* | Tn4*352*::IS*Aba1*, Tn*501*/Tn*1696*, IS*Aba32* |
| **USA15** | pUSA15_1  (98301 bp) | NZ_CP020594.1 | Hamidian et al., 2019 | Rep_3-like  WP_005133531.1 | *aphA6, bla*_OXA-23_ | IS*Aba1, ISAba125, ISAba13* |
| **A85** | pA85-1  (2726 bp) | NZ_CP021783.1 | Hamidian et al., 2014, 2019 | - | - | - |
|  | pA85-1a  (2343 bp) | NZ_CP021784.1 | Hamidian et al., 2019 | - | - | - |
|  | pA85-1b (4484 bp) | NZ_CP021785.1 | Hamidian et al., 2019 | - | - | - |
|  | pA85-2  (8731 bp) | NZ_CP021786.1 | Hamidian et al., 2014, 2019 | RepAci1 (Rep_3 Superfamily) (ALJ89812.1) | - | - |
|  | pA85-3  (86334 bp) | NZ_CP021787.1 | Hamidian et al., 2014, 2019 | Rep_3-like  WP_005133531.1 | *bla*_OXA-23_ | Tn*2006*::IS*Aba1,* Tn*6022*::IS*Aba1* |
| **A388** | pA388  (33036 bp) | NZ_CP024419.1 | Hamidian et al., 2019b | RepAci1 (Rep_3 Superfamily) (ALJ89812.1) | *bla*_OXA-58_, *aphA1b* | ISA*ba2* |
| **AR_0083** | Unnamed  (8731 bp) | NZ_CP027529.1 | Unpublished | RepAci1 (Rep_3 Superfamily) (ALJ89812.1) | - | - |
| **DA33382** | pDA33382-85  (84678 bp) | NZ_CP030109.1 | Nicoloff et al., 2019 | Rep_3-like  WP_005133531.1 | *aph(3')-VI, bla*_OXA-164_ | IS*Aba125,* IS*Aba3*, IS*6,* IS*30,* IS*1* |
|  | pDA33382-2  (2359 bp) | NZ_CP030108.1 | Nicoloff et al., 2019 | - | - | - |
|  | pDA33382-2-2  (2372 bp) | NZ_CP030107.1 | Nicoloff et al., 2019 | 67% identity RepAci1(Rep_3 Superfamily) (ALJ89812.1) | - | - |
| **9102** | pAba9102a  (95206 bp) | NZ_CP023030.1 | Unpublished | PriCT-1 HTH-like (WP_010591570.1) | *aph(3')-VI* | IS*Aba125,* IS*L3* |
| **11W359501** | - | ND | ND | ND | ND | ND |
| **NCTC13421** | - | ND | ND | ND | ND | ND |
| **NIPH 527** | - | ND | ND | ND | ND | ND |
| **NIPH 290** | - | ND | ND | ND | ND | ND |
| **ACICU** | pACICU1 (28279 bp) | NC_010605.1 | Iacono et al., 2008 | RepAci1 (Rep_3 Superfamily) (ALJ89812.1) | *bla*_OXA-58_ | IS*Aba2* |
|  | pACICU2 (64366 bp) | NC_010606.1 | Iacono et al., 2008 | PriCT-1 HTH-like (WP_010591570.1) | *aphA6* | IS*Aba125*,Tn*aphA6* |
| **Naval-13** | pNaval-13 | AMDR01000015 | Chan et al., 2015 | - | *sul2, strA, strB* | IS*1008*, IS*Aha2∆* |
| **A118** | ND | ND | ND | ND | ND | ND |
| **AB33405** | ND | ND | ND | ND | ND | ND |
| **ATCC_17978** | pAB1 (13,408 bp) | CP000522.1 | Smith et al., 2007 | RepAci1-like (Rep_3 Superfamily) (ALJ89812.1) | - | - |
|  | pAB2 (11,302 bp) | CP000523.1 | Smith et al., 2007 | RepAci1 (Rep_3 Superfamily) (ALJ89812.1) | - | - |

^1^ The dash (-) corresponds to negative results for the search by bioinformatics tools.

^2^ “ND” corresponds to Non Determined because it was not pertinent to do the bioinformatics analysis.

^3^ XXX-like refers a DNA sequence that showed more than 40% identity with a XXX-replicase with more than 80% of query cover.
