# Supplementary material for: Crucial Role of the Accessory Genome in the Evolutionary Trajectory of Acinetobacter baumannii Global Clone 1
Source: Front Microbiol. 2020 Mar 18;11:342. doi: 10.3389/fmicb.2020.00342 (PMC7093585; doi:10.3389/fmicb.2020.00342)
Supplement: Supplementary file 12 [file Table_10.docx]

**Supplementary Table 10. Detail of RGP found in A144 genome.**

| **RGP#** | **Detail** |
| --- | --- |
| RGP1 | A144_00407 guaA_1 GMP synthase [glutamine-hydrolyzing] 435440:436168 forward |
| RGP1 | A144_00408 rpsJ 30S ribosomal protein S10 436420:436731 forward |
| RGP1 | A144_00409 rplC 50S ribosomal protein L3 436789:437427 forward |
| RGP1 | A144_00410 rplD 50S ribosomal protein L4 437439:438041 forward |
| RGP1 | A144_00411 rplW 50S ribosomal protein L23 438038:438358 forward |
| RGP1 | A144_00412 rplB 50S ribosomal protein L2 438370:439194 forward |
| RGP1 | A144_00413 rpsS 30S ribosomal protein S19 439208:439483 forward |
| RGP1 | A144_00414 rplV 50S ribosomal protein L22 439495:439824 forward |
| RGP1 | A144_00415 rpsC 30S ribosomal protein S3 439826:440578 forward |
| RGP1 | A144_00416 rplP 50S ribosomal protein L16 440582:440995 forward |
| RGP1 | A144_00417 rpmC 50S ribosomal protein L29 440995:441192 forward |
| RGP1 | A144_00418 rpsQ 30S ribosomal protein S17 441189:441446 forward |
| RGP1 | A144_00419 rplN 50S ribosomal protein L14 441539:441907 forward |
| RGP1 | A144_00420 rplX 50S ribosomal protein L24 441917:442234 forward |
| RGP1 | A144_00421 rplE 50S ribosomal protein L5 442250:442786 forward |
| RGP1 | A144_00422 rpsN 30S ribosomal protein S14 442796:443101 forward |
| RGP1 | A144_00423 rpsH 30S ribosomal protein S8 443113:443508 forward |
| RGP1 | A144_00424 rplF 50S ribosomal protein L6 443522:444055 forward |
| RGP1 | A144_00425 rplR 50S ribosomal protein L18 444070:444420 forward |
| RGP1 | A144_00426 rpsE 30S ribosomal protein S5 444423:444920 forward |
| RGP1 | A144_00427 rpmD 50S ribosomal protein L30 444927:445103 forward |
| RGP1 | A144_00428 rplO 50S ribosomal protein L15 445107:445547 forward |
| RGP1 | A144_00429 secY Protein translocase subunit SecY 445560:446912 forward |
| RGP1 | A144_00430 rpmJ 50S ribosomal protein L36 446933:447049 forward |
| RGP1 | A144_00431 rpsM 30S ribosomal protein S13 447157:447513 forward |
| RGP1 | A144_00432 rpsK 30S ribosomal protein S11 447533:447919 forward |
| RGP1 | A144_00433 rpsD 30S ribosomal protein S4 447932:448558 forward |
| RGP1 | A144_00434 rpoA DNA-directed RNA polymerase subunit alpha 448576:449583 forward |
| RGP1 | A144_00435 rplQ 50S ribosomal protein L17 449602:449979 forward |
| RGP2 | A144_01401 A144_01401 hypothetical protein 1467536:1468807 reverse |
| RGP2 | A144_01402 A144_01402 hypothetical protein 1468804:1469340 reverse |
| RGP2 | A144_01403 A144_01403 hypothetical protein 1469337:1470383 reverse |
| RGP2 | A144_01404 A144_01404 hypothetical protein 1470387:1470749 reverse |
| RGP2 | A144_01405 A144_01405 hypothetical protein 1470757:1471239 reverse |
| RGP2 | A144_01406 A144_01406 hypothetical protein 1471236:1472291 reverse |
| RGP2 | A144_01407 A144_01407 hypothetical protein 1472281:1473456 reverse |
| RGP2 | A144_01408 A144_01408 hypothetical protein 1473536:1475632 reverse |
| RGP2 | A144_01409 A144_01409 hypothetical protein 1475636:1475869 reverse |
| RGP2 | A144_01410 A144_01410 hypothetical protein 1475899:1476210 reverse |
| RGP2 | A144_01412 A144_01412 hypothetical protein 1476719:1477528 reverse |
| RGP2 | A144_01411 A144_01411 hypothetical protein 1476213:1476590 reverse |
| RGP2 | A144_01413 A144_01413 hypothetical protein 1477525:1480164 reverse |
| RGP2 | A144_01414 A144_01414 hypothetical protein 1480175:1480522 reverse |
| RGP2 | A144_01415 A144_01415 hypothetical protein 1480522:1481133 reverse |
| RGP2 | A144_01416 A144_01416 hypothetical protein 1481130:1481561 reverse |
| RGP2 | A144_01417 A144_01417 hypothetical protein 1481573:1482505 reverse |
| RGP2 | A144_01418 A144_01418 hypothetical protein 1482509:1482928 reverse |
| RGP2 | A144_01419 A144_01419 hypothetical protein 1482925:1484010 reverse |
| RGP2 | A144_01420 A144_01420 hypothetical protein 1484099:1484602 reverse |
| RGP2 | A144_01421 A144_01421 hypothetical protein 1484599:1485849 reverse |
| RGP2 | A144_01422 A144_01422 hypothetical protein 1485849:1487468 reverse |
| RGP2 | A144_01423 A144_01423 hypothetical protein 1487746:1488540 forward |
| RGP2 | A144_01424 A144_01424 hypothetical protein 1488537:1490177 reverse |
| RGP2 | A144_01425 A144_01425 hypothetical protein 1490174:1490743 reverse |
| RGP2 | A144_01426 A144_01426 hypothetical protein 1490759:1491052 reverse |
| RGP2 | A144_01427 A144_01427 hypothetical protein 1491055:1491357 reverse |
| RGP2 | A144_01428 A144_01428 hypothetical protein 1491357:1491701 reverse |
| RGP2 | A144_01429 A144_01429 hypothetical protein 1491698:1492198 reverse |
| RGP2 | A144_01430 A144_01430 hypothetical protein 1492277:1492831 reverse |
| RGP2 | A144_01431 A144_01431 hypothetical protein 1492828:1493532 reverse |
| RGP2 | A144_01432 A144_01432 hypothetical protein 1493558:1494034 reverse |
| RGP2 | A144_01433 A144_01433 hypothetical protein 1494127:1494354 forward |
| RGP2 | A144_01434 A144_01434 hypothetical protein 1494399:1494560 forward |
| RGP2 | A144_01435 A144_01435 hypothetical protein 1494560:1494856 forward |
| RGP2 | A144_01436 A144_01436 hypothetical protein 1494869:1495756 forward |
| RGP2 | A144_01437 A144_01437 hypothetical protein 1495759:1497510 forward |
| RGP2 | A144_01438 A144_01438 hypothetical protein 1497520:1498797 forward |
| RGP2 | A144_01439 traR Protein TraR 1498986:1499204 forward |
| RGP2 | A144_01440 A144_01440 hypothetical protein 1499201:1499431 forward |
| RGP2 | A144_01441 A144_01441 hypothetical protein 1499421:1500092 forward |
| RGP2 | A144_01442 A144_01442 hypothetical protein 1500092:1500595 forward |
| RGP2 | A144_01443 A144_01443 hypothetical protein 1500618:1500785 forward |
| RGP2 | A144_01444 hupB_1 DNA-binding protein HU-beta 1500790:1501077 forward |
| RGP2 | A144_01445 A144_01445 hypothetical protein 1501401:1502114 forward |
| RGP2 | A144_01446 A144_01446 hypothetical protein 1502111:1502398 forward |
| RGP2 | A144_01447 A144_01447 hypothetical protein 1502392:1502604 forward |
| RGP2 | A144_01448 A144_01448 hypothetical protein 1502601:1503038 forward |
| RGP2 | A144_01449 A144_01449 hypothetical protein 1503158:1503607 forward |
| RGP3 | A144_01998 A144_01998 hypothetical protein 2085099:2085293 reverse |
| RGP3 | A144_01999 A144_01999 hypothetical protein 2085430:2085972 reverse |
| RGP3 | A144_02000 A144_02000 hypothetical protein 2085983:2086354 reverse |
| RGP3 | A144_02001 A144_02001 O-acetyltransferase WecH 2086549..2087010 |
| RGP3 | A144_02002 A144_02002 hypothetical protein 2087001:2087195 reverse |
| RGP3 | A144_02003 A144_02003 hypothetical protein 2088148:2089980 reverse |
| RGP3 | A144_02004 A144_02004 hypothetical protein 2090231:2091109 reverse |
| RGP3 | A144_02005 A144_02005 hypothetical protein 2091425:2091892 reverse |
| RGP3 | A144_02006 A144_02006 hypothetical protein 2091906:2092373 reverse |
| RGP3 | A144_02007 A144_02007 hypothetical protein 2092370:2094880 reverse |
| RGP3 | A144_02009 A144_02009 hypothetical protein 2097460:2099670 reverse |
| RGP3 | A144_02008 A144_02008 hypothetical protein 2094877:2097453 reverse |
| RGP3 | A144_02010 A144_02010 hypothetical protein 2099670:2100299 reverse |
| RGP3 | A144_02011 A144_02011 hypothetical protein 2100296:2100766 reverse |
| RGP3 | A144_02012 A144_02012 hypothetical protein 2100763:2102799 reverse |
| RGP3 | A144_02013 A144_02013 hypothetical protein 2102800:2103354 reverse |
| RGP3 | A144_02014 A144_02014 hypothetical protein 2103360:2103545 reverse |
| RGP3 | A144_02015 A144_02015 hypothetical protein 2103560:2103964 reverse |
| RGP3 | A144_02016 A144_02016 hypothetical protein 2103978:2105000 reverse |
| RGP3 | A144_02017 A144_02017 hypothetical protein 2105012:2105674 reverse |
| RGP3 | A144_02018 A144_02018 hypothetical protein 2105640:2105972 reverse |
| RGP3 | A144_02019 A144_02019 hypothetical protein 2105969:2107660 reverse |
| RGP3 | A144_02020 A144_02020 hypothetical protein 2107662:2107916 reverse |
| RGP3 | A144_02021 A144_02021 hypothetical protein 2107994:2109616 reverse |
| RGP3 | A144_02022 A144_02022 hypothetical protein 2109949:2110158 reverse |
| RGP3 | A144_02023 A144_02023 hypothetical protein 2110151:2110882 reverse |
| RGP3 | A144_02024 A144_02024 hypothetical protein 2110882:2111091 reverse |
| RGP3 | A144_02025 A144_02025 hypothetical protein 2111306:2113915 reverse |
| RGP3 | A144_02026 A144_02026 hypothetical protein 2114180:2114584 reverse |
| RGP3 | A144_02027 A144_02027 hypothetical protein 2114581:2114832 reverse |
| RGP3 | A144_02028 A144_02028 putative HTH-type transcriptional regulator 2114986:2115612 forward |
| RGP3 | A144_02029 A144_02029 hypothetical protein 2115758:2116216 forward |
| RGP3 | A144_02030 A144_02030 hypothetical protein 2116227:2116361 forward |
| RGP3 | A144_02031 A144_02031 hypothetical protein 2116372:2117664 forward |
| RGP3 | A144_02032 A144_02032 hypothetical protein 2117670:2118233 forward |
| RGP3 | A144_02033 A144_02033 hypothetical protein 2118408:2118734 forward |
| RGP3 | A144_02034 A144_02034 hypothetical protein 2118771:2119619 forward |
| RGP3 | A144_02035 A144_02035 hypothetical protein 2119718:2121784 forward |
| RGP3 | A144_02036 A144_02036 hypothetical protein 2121796:2122278 forward |
| RGP3 | A144_02037 A144_02037 hypothetical protein 2122294:2122461 forward |
| RGP3 | A144_02038 A144_02038 hypothetical protein 2122442:2122711 forward |
| RGP3 | A144_02039 A144_02039 hypothetical protein 2122704:2122961 forward |
| RGP3 | A144_02040 A144_02040 hypothetical protein 2122958:2124352 forward |
| RGP3 | A144_02041 A144_02041 hypothetical protein 2124411:2124662 forward |
| RGP3 | A144_02042 xerC_2 Tyrosine recombinase XerC 2124628:2125587 reverse |
| RGP4 | A144_02125 A144_02125 hypothetical protein 2205766:2206203 reverse |
| RGP4 | A144_02126 allS HTH-type transcriptional activator AllS 2206293:2207192 forward |
| RGP4 | A144_02127 A144_02127 hypothetical protein 2207223:2207321 reverse |
| RGP4 | A144_02128 A144_02128 hypothetical protein 2207775:2209007 reverse |
| RGP4 | A144_02129 A144_02129 tRNA-Met 2209165..2209241 |
| RGP4 | A144_02130 A144_02130 hypothetical protein 2209438:2210301 reverse |
| RGP4 | A144_02131 yxaF_2 putative HTH-type transcriptional regulator YxaF 2210577:2211137 forward |
| RGP4 | A144_02132 A144_02132 hypothetical protein 2211441:2211986 forward |
| RGP5 | A144_02806 A144_02806 hypothetical protein 2889788:2890243 reverse |
| RGP5 | A144_02807 A144_02807 hypothetical protein 2890908:2891144 reverse |
| RGP5 | A144_02808 A144_02808 hypothetical protein 2891353:2891697 reverse |
| RGP5 | A144_02809 A144_02809 hypothetical protein 2892340:2892573 forward |
| RGP5 | A144_02810 A144_02810 hypothetical protein 2892747:2893298 reverse |
| RGP5 | A144_02811 A144_02811 hypothetical protein 2893512:2893730 reverse |
| RGP5 | A144_02812 A144_02812 hypothetical protein 2893976:2894230 reverse |
| RGP5 | A144_02813 A144_02813 hypothetical protein 2894202:2894678 reverse |
| RGP5 | A144_02814 A144_02814 hypothetical protein 2894675:2895076 reverse |
| RGP5 | A144_02815 A144_02815 hypothetical protein 2895076:2895468 reverse |
| RGP5 | A144_02816 A144_02816 hypothetical protein 2895461:2895799 reverse |
| RGP6 | A144_02920 A144_02920 hypothetical protein 2986066:2986344 forward |
| RGP6 | A144_02921 tatD_1 3'-5' ssDNA/RNA exonuclease TatD 2986364:2987104 reverse |
| RGP6 | A144_02922 queC_2 7-cyano-7-deazaguanine synthase 2987104:2988432 reverse |
| RGP6 | A144_02923 A144_02923 hypothetical protein 2988425:2989189 reverse |
| RGP6 | A144_02924 A144_02924 hypothetical protein 2989217:2991010 reverse |
| RGP6 | A144_02925 A144_02925 hypothetical protein 2991121:2992452 reverse |
| RGP7 | A144_03814 comM_1 Competence protein ComM 3954360:3955217 forward |
| RGP7 | A144_03815 A144_03815 hypothetical protein 3955300:3957096 forward |
| RGP7 | A144_03816 bicA_4 Bicarbonate transporter BicA 3957391:3958878 forward |
| RGP7 | A144_03817 A144_03817 hypothetical protein 3958893:3959243 forward |
| RGP7 | A144_03818 A144_03818 hypothetical protein 3959279:3960568 reverse |
| RGP7 | A144_03819 lspA_1 Lipoprotein signal peptidase 3960590:3961102 reverse |
| RGP7 | A144_03820 zitB_2 Zinc transporter ZitB 3961106:3962002 reverse |
| RGP7 | A144_03821 merR1_1 Mercuric resistance operon regulatory protein 3962574:3962981 forward |
| RGP7 | A144_03822 A144_03822 hypothetical protein 3963103:3963366 forward |
| RGP7 | A144_03823 hin_1 DNA-invertase hin 3964012:3964497 reverse |
| RGP7 | A144_03824 A144_03824 hypothetical protein 3964512:3964856 reverse |
| RGP7 | A144_03825 A144_03825 hypothetical protein 3965192:3965686 forward |
| RGP7 | A144_03826 neo Aminoglycoside 3'-phosphotransferase 3966178:3966993 forward |
| RGP7 | A144_03827 hin_2 DNA-invertase hin 3968025:3968684 reverse |
| RGP7 | A144_03828 A144_03828 hypothetical protein 3968795:3970129 reverse |
| RGP7 | A144_03829 A144_03829 hypothetical protein 3970930:3973479 forward |
| RGP7 | A144_03830 A144_03830 hypothetical protein 3973476:3973994 reverse |
| RGP7 | A144_03831 cat_3 Chloramphenicol acetyltransferase 3974467:3975126 forward |
| RGP7 | A144_03832 A144_03832 hypothetical protein 3975327:3975704 reverse |
| RGP7 | A144_03833 A144_03833 hypothetical protein 3975771:3977567 reverse |
| RGP7 | A144_03834 yedA_2 putative inner membrane transporter YedA 3978056:3978940 forward |
| RGP7 | A144_03835 tetA_2 Tetracycline resistance protein, class C 3978972:3980246 reverse |
| RGP7 | A144_03836 tetR_1 Tetracycline repressor protein class A from transposon 1721 3980250:3980927 forward |
| RGP7 | A144_03837 merR_1 Mercuric resistance operon regulatory protein 3981339:3981794 reverse |
| RGP7 | A144_03838 A144_03838 hypothetical protein 3982232:3982549 reverse |
| RGP7 | A144_03839 merC_1 Mercuric transport protein MerC 3982550:3982975 forward |
| RGP7 | A144_03840 merA_1 Mercuric reductase 3983014:3984699 forward |
| RGP7 | A144_03841 A144_03841 hypothetical protein 3984696:3985082 forward |
| RGP7 | A144_03842 A144_03842 hypothetical protein 3985381:3985593 forward |
| RGP7 | A144_03843 A144_03843 hypothetical protein 3985734:3986555 reverse |
| RGP7 | A144_03844 A144_03844 hypothetical protein 3986811:3987023 reverse |
| RGP7 | A144_03845 A144_03845 hypothetical protein 3987322:3987708 reverse |
| RGP7 | A144_03846 merA_2 Mercuric reductase 3987705:3989390 reverse |
| RGP7 | A144_03847 merC_2 Mercuric transport protein MerC 3989429:3989854 reverse |
| RGP7 | A144_03848 A144_03848 hypothetical protein 3989855:3990172 forward |
| RGP7 | A144_03849 merR_2 Mercuric resistance operon regulatory protein 3990610:3991065 forward |
| RGP7 | A144_03850 tetR_2 Tetracycline repressor protein class A from transposon 1721 3991477:3992154 reverse |
| RGP7 | A144_03851 merR1_2 Mercuric resistance operon regulatory protein 3993489:3993896 forward |
| RGP7 | A144_03852 A144_03852 hypothetical protein 3993989:3994516 forward |
| RGP7 | A144_03853 arsC_2 Arsenate reductase 3994719:3995153 forward |
| RGP7 | A144_03855 arsC_3 Arsenate reductase 3995538:3996011 forward |
| RGP7 | A144_03854 arsR1_2 Arsenic resistance transcriptional regulator ArsR1 3995211:3995531 forward |
| RGP7 | A144_03856 acr3_2 Arsenical-resistance protein Acr3 3996019:3997062 forward |
| RGP7 | A144_03857 arsH NADPH-dependent FMN reductase ArsH 3997068:3997772 forward |
| RGP7 | A144_03858 A144_03858 Glucosaminate ammonia-lyase 3997790:3998743 forward |
| RGP7 | A144_03859 czcO putative oxidoreductase CzcO 3998845:3999912 forward |
| RGP7 | A144_03860 A144_03860 hypothetical protein 4000016:4001464 reverse |
| RGP7 | A144_03861 A144_03861 hypothetical protein 4001457:4002572 reverse |
| RGP7 | A144_03862 A144_03862 hypothetical protein 4002602:4003522 reverse |
| RGP7 | A144_03863 tnsB Transposon Tn7 transposition protein TnsB 4003527:4005437 reverse |
| RGP7 | A144_03864 A144_03864 hypothetical protein 4005438:4006148 reverse |
| RGP7 | A144_03865 comM_2 Competence protein ComM 4006273:4006956 forward |
