# Supplementary material for: Crucial Role of the Accessory Genome in the Evolutionary Trajectory of Acinetobacter baumannii Global Clone 1
Source: Front Microbiol. 2020 Mar 18;11:342. doi: 10.3389/fmicb.2020.00342 (PMC7093585; doi:10.3389/fmicb.2020.00342)
Supplement: Supplementary file 14 [file Table_12.docx]

**Supplementary Table 12. Analysis of the spacers from A144 CRISPR-Cas system.**

| Spacer | **Best Hit** | **Identities** | **Identity %** |
| --- | --- | --- | --- |
| 1 | KJ740398.1 *Acinetobacter* phage RL-2015 clone PlyF311 endolysin gene | 21/32 | 91 |
| 2 | MG592623.1 *Vibrio* phage 1.257.O._10N.286.46.A4 | 18/32 | 100 |
| 3 | KX130862.1 *Shigella* phage SHFML-26, complete genome | 19/32 | 95 |
| 4 | CP000029.1 *Staphylococcus* epidermidis RP62A phage SP-beta | 23/32 | 88 |
| 5 | MG592456.1 *Vibrio* phage 1.081.O._10N.286.52.C2 | 16/32 | 100 |
| 6 | MH333064.1 *Klebsiella* phage Mineola | 20/32 | 95 |
| 7 | MF428481.1 *Staphylococcus* phage SN8 | 29/32 | 86 |
| 8 | LC371242.1 *Escherichia* phage EcS1 | 24/32 | 92 |
| 9 | GU071094.1 *Synechococcus* phage S-SM1 | 23/32 | 96 |
| 10 | KM236245.1 *Bacillus* phage Mater, | 24/32 | 92 |
| 11 | MG592425.1 *Vibrio* phage 1.042.O._10N.286.45.B8 | 23/32 | 91 |
| 12 | CP002121.1 *Streptococcus* phage PhiSpn_200 | 24/32 | 92 |
| 13 | CP000029.1 *Staphylococcus epidermidis* RP62A phage SP-beta | 18/32 | 100 |
| 14 | KR060090.1 *Pseudoalteromonas* phage Pq0 | 25/32 | 96 |
| 15 | MK250029.1 *Prevotella* phage Lak-C1 | 29/32 | 86 |
| 16 | MH937489.1 *Streptococcus* phage CHPC1034 | 25/32 | 92 |
| 17 | MH333064.1 *Klebsiella* phage Mineola | 19/32 | 95 |
| 18 | MG766219.2 *Staphylococcus* phage vB_SauP_phiAGO1.9 | 19/32 | 95 |
| 19 | KR296694.1 *Salmonella* phage 40 | 21/32 | 90 |
| 20 | MG878892.2 *Salmonella* phage vB_SpuP_Spp16 | 19/32 | 95 |
| 21 | MF140401.1 *Arthrobacter* phage Caterpillar | 19/32 | 95 |
| 22 | CP000233.1 *Lactobacillus* phage Sal2 | 27/32 | 85 |
| 23 | MK047718.1 *Escherichia* phage p000y | 21/32 | 95 |
| 24 | CP029707.1 *Pseudomonas* phage AK-2018a | 21/32 | 90 |
| 25 | EU719189.1 *Clostridium* phage phiCD27 | 22/32 | 95 |
| 26 | KY349816.1 *Streptococcus* phage Str01 | 21/32 | 95 |
| 27 | HQ316584.1 *Cyanophage* SS120-1 | 25/32 | 92 |
| 28 | HQ630627.1 *Pseudomonas* phage PhiPA3 | 29/32 | 86 |
| 29 | AE017333.1 *Bacillus* phage BLi_Pp2 | 25/32 | 92 |
| 30 | LC121084.1 *Ralstonia* phage RSP15 | 25/32 | 88 |
| 31 | MG459987.1 *Klebsiella* phage Sugarland | 21/32 | 95 |
| 32 | LC371242.1 *Escherichia* phage EcS1 | 17/32 | 100 |
| 33 | KP793133.1 *Lactococcus* phage 936 group phage PhiLj | 25/32 | 92 |
| 34 | NC_028777.1 *Bacillus* phage Stills | 23/32 | 92 |
| 35 | AY682195.1 *Lactobacillus plantarum* bacteriophage LP65 | 20/32 | 95 |
| 36 | AY539836.1 *Burkholderia cenocepacia* phage BcepMu | 16/32 | 100 |
| 37 | MG746602.1 *Klebsiella* phage vB_Kpn_F48 | 19/32 | 95 |
| 38 | JX181829.1 *Salmonella* phage SKML-39 | 20/32 | 95 |
| 39 | MH155872.1 *Streptomyces* phage Moozy | 20/32 | 95 |
| 40 | MK165087.1 *Escherichia* phage BRET | 21/32 | 90 |
| 41 | KY971610.1 *Pseudomonas* phage PspYZU05 | 20/32 | 95 |
| 42 | KY065489.1 *Streptococcus* phage IPP51 | 27/32 | 85 |
| 43 | MH517022.1 *Acinetobacter* phage SH-Ab 15599 | 19/32 | 95 |
| 44 | CP002121.1 *Streptococcus* pneumoniae phage PhiSpn_200 | 20/32 | 95 |
| 45 | MF285618.1 *Serratia* phage 2050HW | 21/32 | 90 |
| 46 | MK308638.1 *Microbacterium* phage ArMaWen | 19/32 | 95 |
| 47 | CP001844.2 *Staphylococcus aureus* phage phiSaST5K | 26/32 | 88 |
| 48 | MG757157.1 *Gordonia* phage Flapper | 19/32 | 95 |
| 49 | MF036692.1 *Serratia* phage X20 | 26/32 | 92 |
| 50 | MF063068.1 *Pseudomonas* phage Noxifer | 22/32 | 91 |
| 51 | MG592441.1 *Vibrio* phage 1.063.O._10N.261.45.C7 | 17/32 | 100 |
| 52 | KX268652.1 *Acinetobacter* phage vB_AbaS_TRS1 | 29/32 | 93 |
